# Evolution and Design Governing Signal Precision and Amplification in a Bacterial Chemosensory Pathway

**DOI:** 10.1371/journal.pgen.1005460

**Published:** 2015-08-20

**Authors:** Mathilde Guzzo, Rym Agrebi, Leon Espinosa, Grégory Baronian, Virginie Molle, Emilia M. F. Mauriello, Céline Brochier-Armanet, Tâm Mignot

**Affiliations:** 1 Laboratoire de Chimie Bactérienne, Institut de Microbiologie de la Méditerranée, CNRS Aix-Marseille University UMR 7283, Marseille, France; 2 Laboratoire de Dynamique des Interactions Membranaires Normales et Pathologiques, CNRS Universités de Montpellier II et I, UMR 5235, case 107, Montpellier, France; 3 Université de Lyon, Université Lyon 1, CNRS, UMR5558, Laboratoire de Biométrie et Biologie Evolutive, Villeurbanne, France; University of Geneva Medical School, SWITZERLAND

## Abstract

Understanding the principles underlying the plasticity of signal transduction networks is fundamental to decipher the functioning of living cells. In *Myxococcus xanthus*, a particular chemosensory system (Frz) coordinates the activity of two separate motility systems (the A- and S-motility systems), promoting multicellular development. This unusual structure asks how signal is transduced in a branched signal transduction pathway. Using combined evolution-guided and single cell approaches, we successfully uncoupled the regulations and showed that the A-motility regulation system branched-off an existing signaling system that initially only controlled S-motility. Pathway branching emerged in part following a gene duplication event and changes in the circuit structure increasing the signaling efficiency. In the evolved pathway, the Frz histidine kinase generates a steep biphasic response to increasing external stimulations, which is essential for signal partitioning to the motility systems. We further show that this behavior results from the action of two accessory response regulator proteins that act independently to filter and amplify signals from the upstream kinase. Thus, signal amplification loops may underlie the emergence of new connectivity in signal transduction pathways.

## Author Summary

Deciphering the circuit design of signal transduction networks is a fundamental question in cell biology. This task is challenging because many pathways are branched and control multiple cellular processes in response to one or several environmental signals. Studying pathway diversification in bacteria could be a powerful approach because these organisms contain so-called chemosensory systems, modular signaling units that have been adapted multiple times independently to regulate a large number of physiological processes. Here, we studied one such system, the *Myxococcus xanthus* chemosensory pathway (Frz) that controls the directionality of two distinct motility systems (A- and S-motility). By experimentally uncoupling the regulations, we found that the Frz pathway evolved from a simpler ancestral system that only controlled S-motility originally. Two major pathway remodeling events allowed the recruitment of A-motility to the regulation, (i) the duplication of a connector protein which created the branch point and (ii), the acquisition of a signal amplification mechanism to allow signal partitioning at the branch point. These results reveal the core structure of a complex chemosensory system and generally suggest that gene duplication and signal amplification underlie the diversification of signal transduction pathways.

## Introduction

In living cells, adaptation to rapid changes in environmental conditions requires coordinated rearrangements of basic cellular processes to adjust the cellular homeostasis to the new conditions. In general, receptor molecules sense environmental changes and translate them into a cellular response by phosphorylation of a downstream regulator. Because the cellular response must be integrated to various cellular processes, the phosphorylation cascade frequently involves a number of intermediates, allowing multiple regulation layers and branch points (nodes) in the regulatory circuit [1,2]. Thus, for a given pathway, identifying nodes and understanding how they participate in the regulation is of fundamental importance to elucidate how signals are integrated toward a cellular response. One possible approach to elucidate the underlying structure of a multi-component signaling pathway is to study its evolution because the diversification of signaling pathways is under strong selection pressure and signaling intermediates may have been selected in some organisms [3]. Consequently, some proteins that appear central to the regulation in a given genetic context may in fact be dispensable in a different context where their function is not required. For example, a protein that insulates a pathway from another will become dispensable if the secondary pathway is removed. Thus, tracking back the evolutionary history of signaling pathways can reveal core regulatory motifs and principles underlying the acquisition of additional regulations [4,5]. Bacteria are exceptional model systems for such studies because they are highly tractable experimentally and thousands of genome have been sequenced.

In bacteria, signal transduction networks are frequently formed by so-called two-component systems. The core motif of a two-component system generally consists of a receptor, generally a membrane localized sensor histidine kinase (HK) and its cognate response regulator (RR). Following activation by environmental signals, the HK uses ATP to autophosphorylate on a conserved histidine residue and the phosphoryl group is transferred to a conserved Asp residue of the RR protein, regulating a number of downstream processes, gene expression, secondary messenger synthesis or protein-protein interaction [6]. HK/RR pairs often form autonomous signal transduction systems [7], but they are also modular and can be incorporated in more complex circuits, multiple phosphorelay systems and chemosensory-type systems [1,8]. In the enteric chemotaxis (Che) system, the HK (CheA) does not act directly as a sensor but resides in the cytosol where it is activated by a transmembrane Methyl-accepting protein (Mcp) via a coupling protein (CheW). Following activation, CheA transfers a phosphoryl group to two RR domains; one of them, the CheY protein constitutes the system output and interacts with a protein of the flagellum (FliM) to switch the direction of its rotation. The second RR domain is carried by the CheB methyl esterase, and its phosphorylation activates the de-methylation of the Mcp to reset the system to a pre-signaling state (adaptation, for a review, see [9]). In many bacteria, the core signaling apparatus of the enteric Che system has been coopted to the regulation of processes other than chemotaxis, such as surface motility, gene regulation and even cellular differentiation [8,10,11]. The genetic structure of these non-canonical chemosensory systems is quite diverse and their circuit architecture is generally poorly understood [8,10,11]. In this study, we investigate the evolution and genetic structure of a chemosensory-type system that controls two distinct motility machineries in *Myxococcus xanthus*.

**Fig 1:**
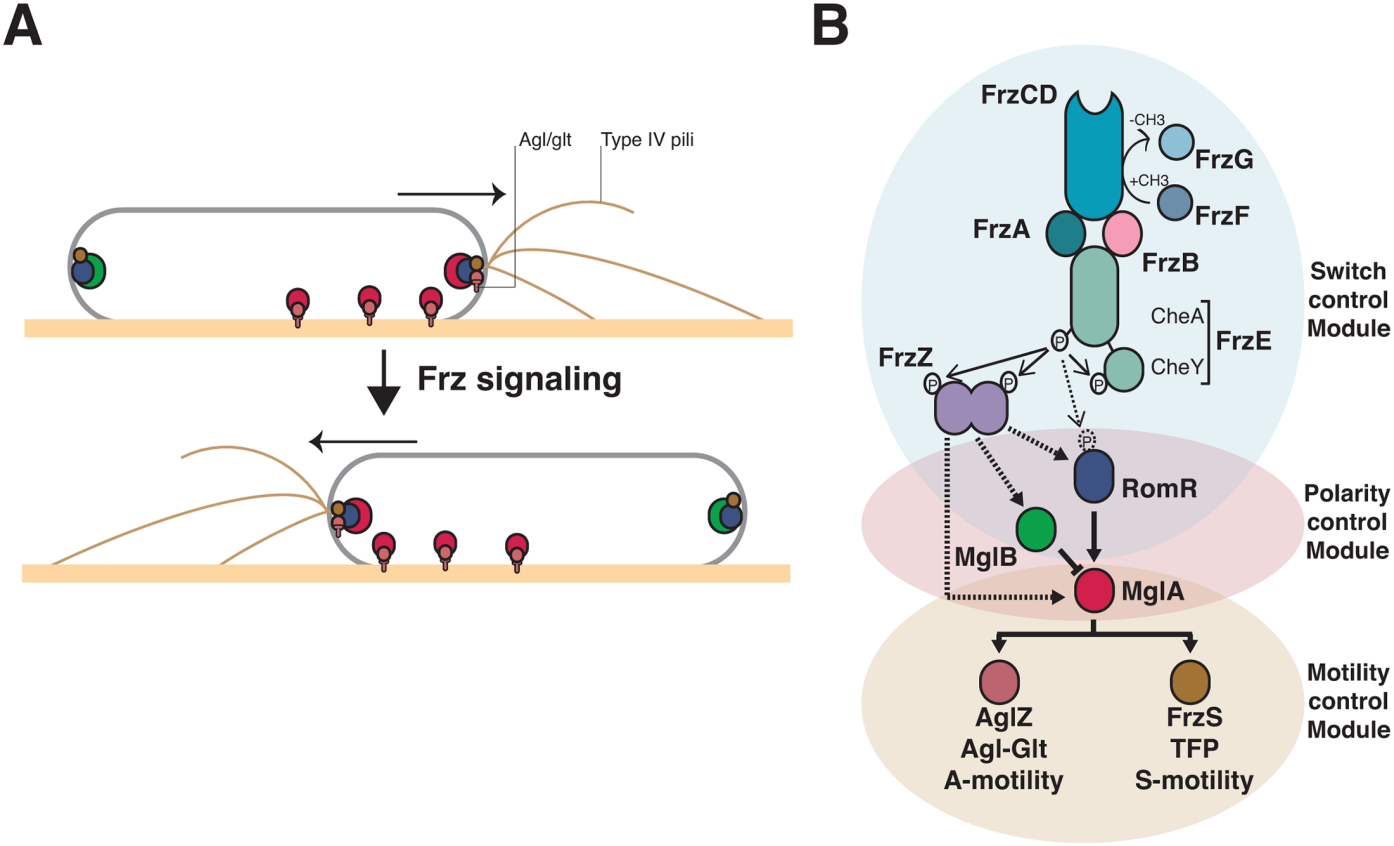
Genetic control of the *Myxococcus* A- and S-motility machineries. **(A)** Dynamics of the motility machineries and their associated regulators during the reversal cycle. MglA (Red circle) is recruited by RomR (Blue circle) to the leading cell pole where it activates A- motility (Agl/Glt, T-shapes) and S-motility (Type-IV pili) at least partially through AglZ and FrzS, respectively (Crimson and Brown circles). At the lagging cell pole, MglB (green circle) co-localizes with RomR and FrzS where it prevents MglA accumulation by activating GTP hydrolysis by MglA. Frz signaling provokes the concerted polarity switching of MglA and MglB to opposite poles, allowing A- and S-motility activation at the new leading and movement in the opposite direction. **(B)** Genetic control of the reversal cycle. Schematic of the regulation cascade compiled from previous works. Frz signaling is thought to promote a phosphorylation cascade that activates reversals. In more details, activation of the FrzCD receptor by unknown signals activates the auto-phosphorylation of the FrzE kinase through the FrzA CheW-like protein. The exact function of FrzB, another CheW-like protein, is unknown, contrarily to FrzA it is not absolutely essential for the activation of FrzE. FrzE then transfers a phosphoryl group to up to three response regulator proteins, its cognate receiver domain (FrzE^RR^), the FrzZ protein, a fusion of two RR domains, and the N-terminal RomR receiver domain. The outcome of these phosphorylation events is unknown but the phosphorylated output(s) is thought to interact directly with the polarity proteins and provoke their re-localization to opposite poles. Plain arrows indicate established interactions and dotted arrows indicate suspected interactions. The protein color code applies throughout the manuscript.

*Myxococcus xanthus*, a gram negative deltaproteobacterium, uses surface motility to form multicellular spore-filled fruiting bodies when nutrient sources become scarce. During this process, the *Myxococcus* cells can move as single cells or within large coordinated cell groups and reverse their direction of movement in a process where the cell poles rapidly exchange roles [12]. In the cell groups, a Type-IV pilus (Tfp) assembled at the cell pole (the leading pole) acts as grappling hooks and pulls the cell forward by retraction (Fig 1A, [13]). Tfps retract when they are in contact with a cell surface-exposed exopolysaccharide (fibrils), giving rise to a cooperative form of group movements called S (Social)-motility [14]. At the colony edges, the single cells are propelled by the recently characterized Agl-Glt apparatus, otherwise called the A (Adventurous)-motility system [15,16]. The Agl-Glt complex is assembled at the leading cell pole and moves directionally towards the lagging cell pole, promoting movement when it contacts the underlying surface (Fig 1A, [12,16,17]). Thus, both motility systems are activated at the leading cell pole and their activation is switched coordinately to the opposite cell pole when cells reverse (Fig 1A, [18–20]). The frequency of the reversal events is under the genetic control of a chemosensory-like system called Frz [21]. This control is essential for *Myxococcus* multicellular behaviors because *frz* mutants that reverse at low frequencies do not form fruiting bodies and form characteristic “frizzy” filament structures [22].

The molecular link between Frz and the motility systems requires three intermediate polarity proteins, MglA, MglB and RomR (Fig 1A). MglA, a bacterial Ras-like G-protein, activates the motility systems at the leading cell pole (Fig 1A, [23,24]). As all members of this family of molecular switches, MglA is active in association with GTP, a form that interacts with two motility system-specific proteins, AglZ (A-motility) and FrzS (S-motility) [19,20,25]. The polar localization of MglA results from combined regulations: (i), by RomR, which recruits MglA-GTP to the cell poles [26,27]; and (ii), by MglB, a MglA GTPase Activating Protein (GAP), which prevents MglA access to the lagging cell pole (Fig 1A, [23,24,28]). The polarity axis formed by MglA, MglB and RomR is stable unless it is contacted by upstream Frz signals, which provokes its dynamic switching and a reversal (Fig 1A, [29]).

*In vivo*, unknown signals are sensed by the Mcp homologue (FrzCD) and lead to the autophosphorylation of the kinase of the system (FrzE) from ATP via FrzA, the major CheW-like coupling protein (Fig 1B, [30]. FrzB, another CheW-like homolog may also participate in the activation of the pathway, although contrarily to FrzA, it is not required for all Frz-dependent responses (Fig 1B, [31]). Following activation, FrzE may then activate a handful of RR domains, including the C-terminal RR domain of FrzE (FrzE^RR^), the FrzZ protein and the N-terminal RR domain of RomR itself, to contact the polarity proteins and activate the polarity switch (Fig 1B, [30,32–34]). Thus, the presence of multiple RR domains and a branch point at a circuit node formed by MglA asks how signaling from the FrzE kinase is channeled to the downstream motility systems (Fig 1B).

Overall, the motility regulation circuit is assembled from four interconnected modules with genetically separable functions: the control of the reversal frequency (switch control module), the coordination of the A- and S-motility systems (polarity control module) and the function of A- and S-motility (A- and S-motility modules, Fig 1B). By investigating the evolutionary origin of each module, we identified the core structure of the Frz pathway, genetically uncoupled A- and S-motility regulations and thus identified key regulations that led to the emergence of the evolved pathway. Remarkably, we find that adaptation of the ancestral circuit to A- and S-motility regulation required amplification of the signaling efficiency, suggesting that the evolution of signal reinforcement mechanisms may be linked to signal transduction pathway diversification in cells.

## Results

### S-motility regulation could be ancestral to the acquisition of A-motility in *Myxococcus*

**Fig 2:**
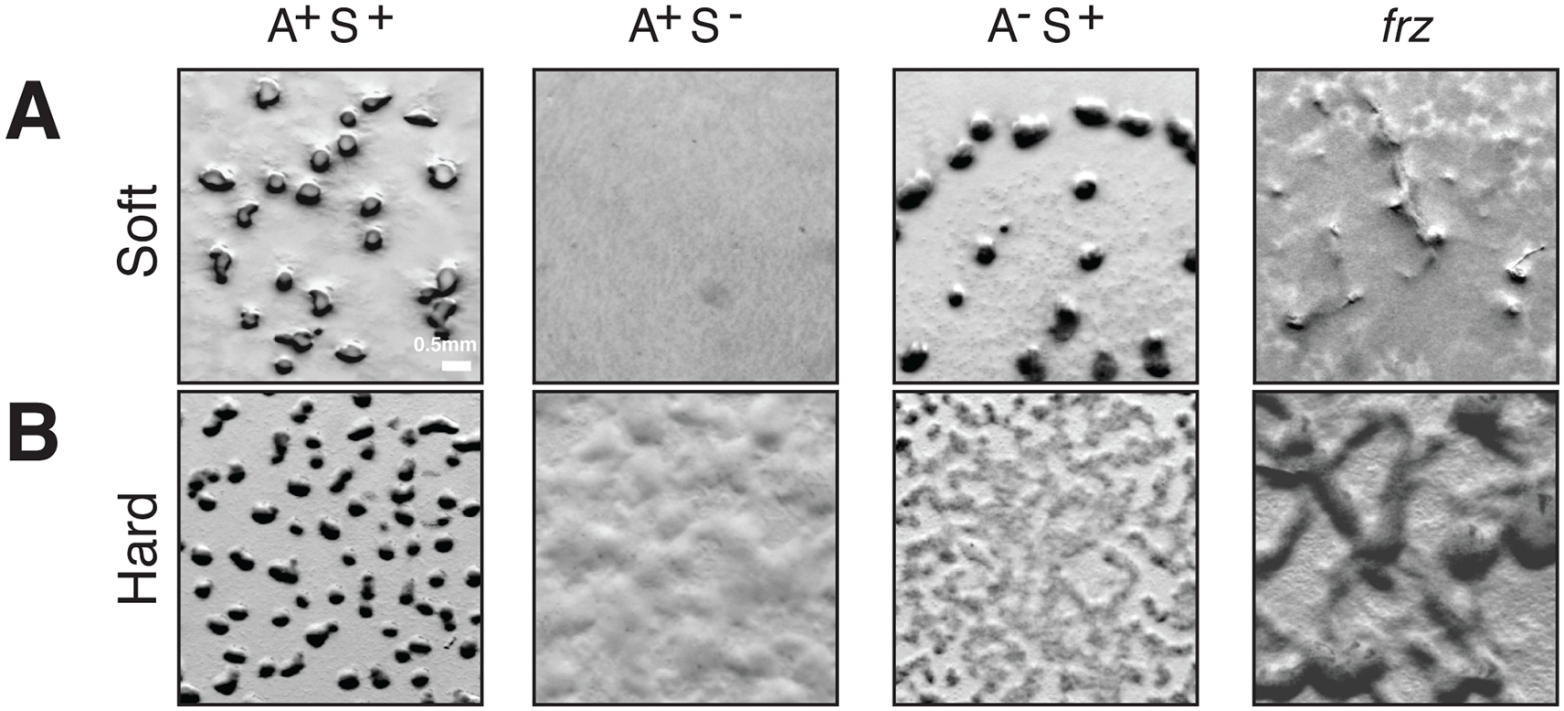
A- and S-motility diversify the repertoire of *Myxococcus* multicellular behaviors. **(A)** Fruiting body formation on a soft agar surface in WT and mutants. WT, *pilA* (A^+^S^−^), *aglZ* (A^−^S^+^) and *frzE* (*frz*) null mutants were tested for their ability to form fruiting bodies on 0.5% CF starvation medium agar plates (Soft). Fruiting bodies are apparent as dense black aggregates. **(B)** Fruiting body formation on a hard agar surface in WT and mutants. The same strains as in (A) were tested for their ability to form fruiting bodies on 1.5% CF starvation medium agar plates (Hard). Fruiting bodies are apparent as dense black aggregates.

From an evolutionary perspective, the co-regulation of the A- and S-motility complexes by the Frz system implied the connection of two machineries of different origins. While Tfp systems are found in all deltaproteobacterial genomes [26,35], the A-motility Agl-Glt machinery is only present in *Cystobacterineae*, a Deltaproteobacteria family [15,16,36]. Thus, S-motility may be more ancient than A-motility and the emergence of A-motility in *Cystobacterineae* could have expanded the phenotypic repertoire and adaptive capabilities of this family of bacteria. Accordingly in *Myxococcus*, S-motility is required for fruiting body formation on soft and hard surfaces (0.5% *vs* 1.5% agar) but A-motility is only required on hard surfaces (Fig 2A and 2B). Importantly however, Frz regulation is required on both types of surfaces (Fig 2A and 2B). To further understand how co-regulation of A- and S-motility emerged in *Cystobacterineae*, we performed in-depth phylogenetic analyses of the components of the switch, polarity and motility control modules.

#### Polarity control module

**Fig 3:**
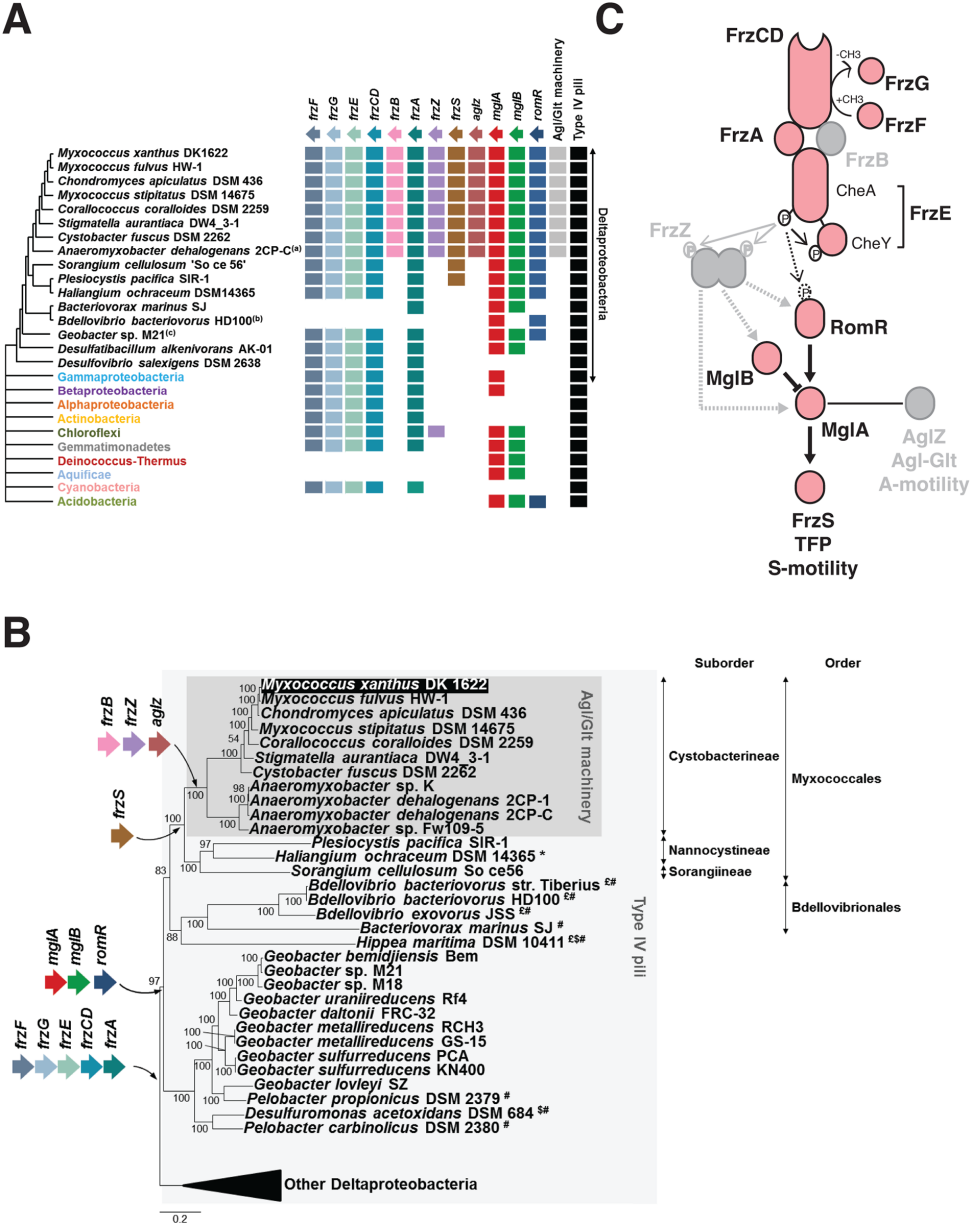
The taxonomic distribution of the *Myxococcus* motility suggests a new structure of the regulation pathway. **(A)** Taxonomic distribution of the motility genes. The occurrence of each gene is inferred from the individual phylogenies (S1, S3 and S6 Figs). The phylogenetic relationships between the deltaproteobacterial species shown are inferred from the analysis of 17 genes in total, which are present in a single copy in the deltaproteobacterial genomes (S5 Table). The presence of the Agl-Glt machinery in genomes is inferred from work published in [16]. The presence of Type-IV pili is based on the occurrence of PilM, N, O, P, Q homologues that form a core Tfp-structure, identified from the literature or by BLAST analysis of genomes of interest. **(B)** Acquisition of the motility genes in the Deltaproteobacteria. $: possible loss of RomR; £: possible loss of MglB; *: possible loss of FrzS; #: possible loss of FrzF, G, E, CD and A. **(C)** Modular structure of the regulatory pathway. The taxonomic distribution of the motility genes (A,B) regulation pathway may consist of a core signaling apparatus (pink) that regulates S-motility following the direct phosphorylation of RomR by the FrzE kinase. The acquisition of FrzZ, FrzB and AgZ coincides with the emergence of the A-motility system in the *Cystobacterineae* and could have adapted the regulatory circuit to the control of two outputs. This scenario predicts that FrzZ, thought to be central to the regulation is rather an accessory that adapted the phosphate flow upstream from MglA.

The *Myxococcus xanthus* MglA protein belongs to a large protein family of MglA-like proteins, and more precisely to the group 1 as defined by Wuichet and Sogaard Andersen ([37], S1A Fig). A total of 146 MglA group 1 homologues were found in 88 bacterial genomes, mainly in the Deltaproteobacteria (80 sequences in 38 genomes) (Figs 3A and S1A). MglB presents a similar taxonomic distribution, 90 sequences were detected in 90 genomes, 36 of which belonging to *Cystobacterineae* (for a total of 36 genomes, Figs 3A and S1B). When MglA and MglB were found together in a genome, they were encoded by tandem-genes, underscoring the functional link between these two proteins (S2 Fig). Because MglB is an MglA regulator, the absence of *mglB* in genomes where *mglA* is present could be linked to the loss of MglA regulation in these bacteria [38]. RomR paralogues showed a more narrow distribution, with the exception of *Acidobacteria*, they were only identified in 27 deltaproteobacterial genomes, again mainly in *Cystobacterineae* (Figs 3A and S1C). Interestingly, nearly all these genomes also contain MglA and MglB homologues, further supporting that MglA, MglB and RomR form a functional module. These results are for the most part largely consistent with results by Keilberg et al., except for minor differences in the RomR phylogenies, likely due to different strategies in RomR sequence identifications ([26], see Experimental procedures). All together these results suggest that the *Myxococcus* polarity module was acquired early during the diversification of the Deltaproteobacteria (Fig 3B). Given that MglA and to a lesser extent MglB and RomR, are required for the regulation and the function of S-motility [23,24,26,27], S-motility could have evolved following their acquisition and the cooptation of a deltaproteobacterial Tfp ancestor.

#### Switch control module

The Frz system deviates from the enteric Che system and contains an Mcp (FrzCD), two CheW homologues (FrzA and FrzB), a hybrid kinase (FrzE), a methyl-transferase (FrzF), a methyl-esterase (FrzG) and an unusual tandem response regulator domains containing protein, FrzZ [31]. Based on our analysis, a first group of Frz homologues, composed of the core signaling proteins Mcp (FrzCD), a CheW-like protein (FrzA), a hybrid CheA-type kinase (FrzE) and the methyl transferase/esterase system (FrzFG), presents similar taxonomic distributions. More precisely, these genes are found in a number of deltaproteobacterial genomes, but also in a few representatives of various bacterial phyla and classes (e.g. *Gammaproteobacteria*, *Betaproteobacteria*, *Chloroflexi*, *Cyanobacteria*) (Figs 3A and S3A-S3E). The genes are always clustered together in bacterial genomes and their phylogenies are globally consistent, indicating similar evolutionary histories (including horizontal gene transfer of single blocks, S3, S4 and S5 Figs). In contrast, the second group including FrzB (a CheW-homolog) and remarkably, FrzZ (the RR-RR fusion protein) appears limited to *Cystobacterineae* (Figs 3A, S3F and S3G), pointing to a more recent evolutionary origin. In *Cystobacterineae*, two important Frz protein domain modifications suggest major changes in the sensing capability of the system: (i), in the FrzCD protein, the transmembrane segments are replaced by a soluble N-terminal domain of unknown function [31], suggesting that FrzCD senses cytosolic signals in this group of bacteria (S3A Fig and S4 Table); (ii), the RR domain of the FrzG methylesterase is largely degenerate (more specifically in the *Cystobacteraceae* and *Myxococcaceae* but not in the *Anaeromyxobacteraceae*, S3E Fig and S4 Table). The recent loss of a functional RR domain in FrzG suggests important changes in the adaptation mechanism; in fact and contrarily to the proteins of the first group, FrzG is not absolutely required for Frz signaling, suggesting that its core function evolved in *Cystobacterineae* [31].

#### Connection to the A- and S-motility modules

How MglA contacts the motility systems is not completely understood but it may occur, at least partially, through an interaction with AglZ and FrzS, two homologous proteins that co-localize with the A- and S-motility systems respectively [25,39]. Remarkably, AglZ and FrzS contain similar protein domains: an N-terminal receiver domain and an extended coiled-coil C-terminal domain (S4 Table). In AglZ, the C-terminal coiled-coil is 1076 residues long while it is 235 residues long in FrzS (S4 Table). In FrzS, the N-terminal domain is essential for the localization of this protein at the leading cell pole [40]. Although this domain folds like a typical receiver domain, it cannot accept a phosphoryl group because an Alanine residue replaces the critical Asp in the catalytic pocket [41]. Because the exact FrzS and AglZ MglA-binding sites have not been mapped, it is not known if these proteins use similar binding motifs to interact with MglA. Our phylogenetic analysis reveals that FrzS and AglZ are paralogues and indicates that they emerged following a gene duplication event that occurred in an ancestor of *Cystobacterineae* (Figs 3A and S6). FrzS may be the ancestral form and AglZ a derived form because the unique copy present in *Cystobacterineae* relatives is more similar to FrzS (Figs 3A, 3B, and S6). None of the FrzS and AglZ paralogues contain a functional phosphorylation site, suggesting that the receiver function of the response regulator domain is generally decayed in this protein group (S7 Fig). Worth noticing, the duplication event and the emergence of A-motility occurred in the same branch of the Deltaproteobacteria tree, namely in the ancestor of *Cystobacterineae* (Fig 3B). Thus, co-option of the new derivative (*ie* AglZ) by the Agl-Glt machinery could have provided an effective solution to branch the two motility systems to the same regulation pathway downstream from MglA.

In summary, the *Myxococcus* motility control proteins can be divided in two classes based on their phylogenetic distribution. Remarkably, the acquisition of FrzB, FrzZ and AglZ correlates with the emergence of the Agl-Glt machinery in an ancestor of the *Cystobacterineae* (Fig 3A and 3B), suggesting that these modifications integrated A-motility regulation to a previously existing S-motility regulation pathway (Fig 3C). This hypothesis predicts that a simpler pathway lacking FrzZ, FrzB and AglZ should be sufficient to regulate S-motility- but not both A- and S-motility-dependent behaviors (Fig 3C), which we decided to test experimentally.

### FrzZ, FrzB and AglZ are not required for S-motility dependent reversals

**Fig 4:**
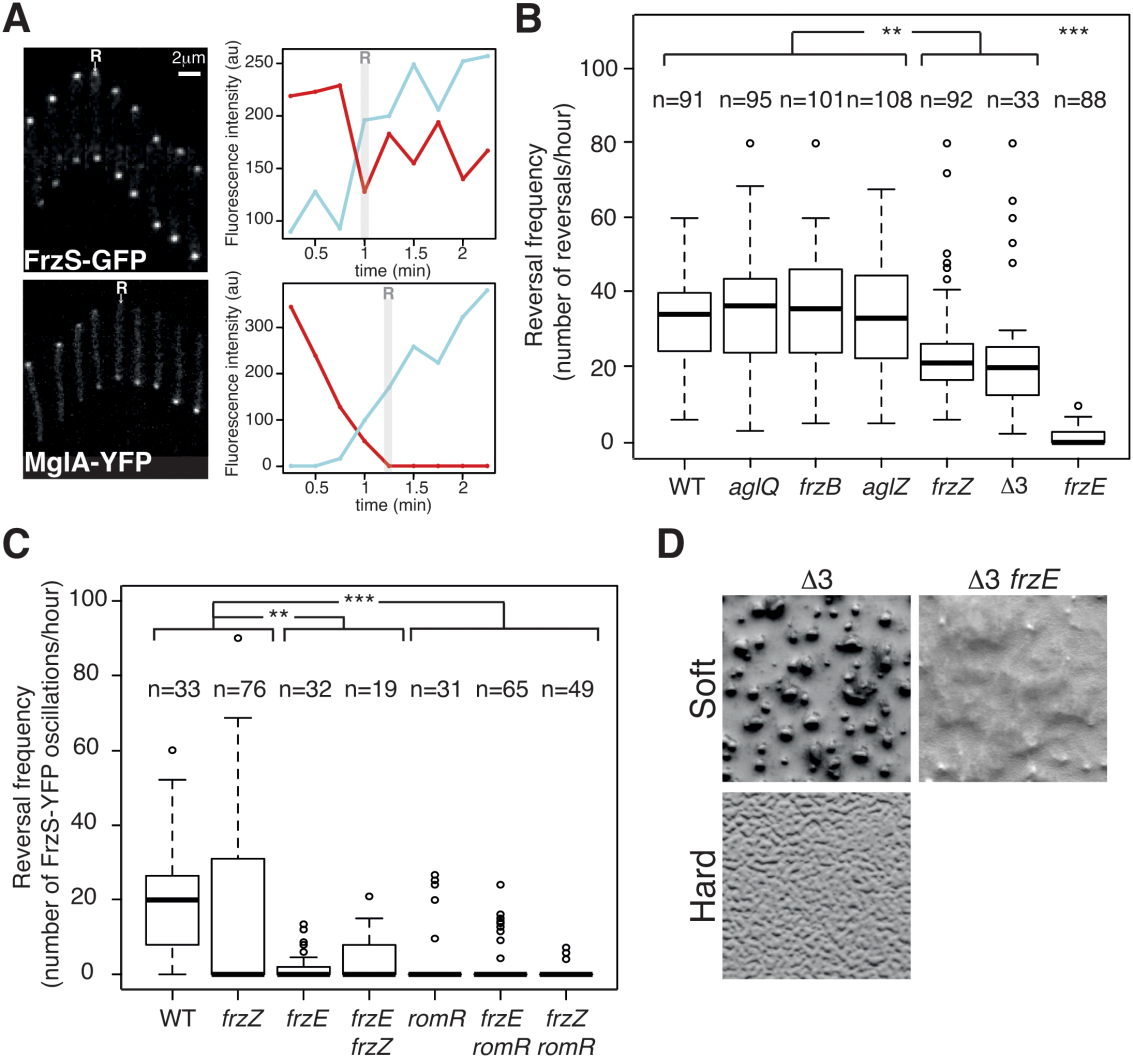
The core Frz-apparatus can promote S-motility-dependent behaviors. **(A)** Single cell Tfp-dependent reversals are observed in carboxymethylcellulose-coated glass microfluidic chambers. FrzS-YFP and MglA-YFP dynamic localization are observed during single cell Tfp-dependent reversals. Fluorescence micrographs (left) and quantification of the corresponding fluorescence intensities (arbitrary units, au) of FrzS-YFP and MglA-YFP at both poles over time are shown (time resolution: 15s). Red line: initial leading pole, blue line: initial lagging pole (Frame 1). R: reversals. **(B)** Reversal frequency of WT and selected mutants on carboxymethylcellulose. The assays are run in the presence of 0.15% of Isoamyl alcohol (IAA) to allow reversal counts (see Methods). Shown are boxplots of the measured reversal frequency of isolated cells (n, tracked for at least 10 min) for each strain. The lower and upper boundaries of the boxes correspond to the 25% and 75% percentiles, respectively. The median is shown as a thick black line and the whiskers represent the 10% and 90% percentiles. Statistics: Wilcox test (n<40) for Δ3 (*frzB frzZ aglZ*) and student test (t-test, n>40) for all the other strains. ** p value<0.01, *** p value<0.0001. **(C)** RomR but not FrzZ is essential for S-motility-dependent reversals. FrzS-YFP pole-to-pole oscillations were counted in the presence of 0.10% of IAA to score reversals (the exact procedure is described in Methods and S9A and S9B Fig). Briefly, for each pole, fluorescence intensity was measured at any given time point as described in Methods and S9A and S9B Fig. The boxplots read as in (B). Statistics: Wilcox test (n<40) for WT, *frzE*, *romR*, *frzE frzZ* or student test (t-test, n>40) for *frzZ*, *frzE romR* and *frzZ romR*. ** p value<0.01, *** p value<0.0001. **(D)** Multicellular development is restored in a triple mutant *frzB frzZ aglZ* (Δ3) on soft (0.5%) CF starvation medium agar in a Frz-dependent manner (compare with the Δ3 *frzE* mutant). On the contrary the *frzB frzZ aglZ* cannot aggregate on a hard (1.5%) CF agar, where A-motility is also required.

Measuring reversals of the S-motility system is difficult because they occur in large cell groups where single cells cannot be easily tracked. As mentioned in the introduction, S-motility results from the action of an extracellular EPS that provokes Tfp retraction [14]. In fact, the requirement for EPS can be bypassed, in single cell assays where the *Myxococcus* cells are allowed to move in a carboxymethylcellulose-coated microfluidic chamber (See Experimental procedures). In this system, cells move by Tfp-dependent motility only with an average speed of 1.7 ± 0.8 μm.min^-1^ (measured for 63 cells) and Frz-dependent cellular reversals are observed and coincide with pole-to-pole oscillations of MglA-YFP and FrzS-YFP, similar to agar (Figs 4A, 4B, and S8A) [19,24]. A-motility is not active on the cellulose surface because the cell velocity and the reversal frequency of an A-motility A^−^ (*aglQ*) mutant are unchanged compared to WT cells (1.6 ± 0.9 μm.min^-1^, for 61 cells, S1 Movie and Fig 4B). Therefore, we used A^+^ strains for the rest of this study.

We used the cellulose assay and high-throughput automated tracking (S8B Fig and S2 Movie) to test whether the core pathway defined by the phylogenetic analysis is sufficient to regulate Tfp-dependent reversals. Remarkably, Tfp-dependent reversals were still observed in the absence of each of the predicted accessory components, FrzZ, FrzB and AglZ (Fig 4B). The frequency of Tfp-dependent reversals was equivalent to WT levels in the *frzB* and *aglZ* mutants, showing that these proteins are dispensable for the control of Tfp-dependent reversals (Fig 4B). In the case of the *frzZ* mutant, the situation was intermediate, the reversal frequency was affected compared to WT cells but it was still significantly higher than in the *frzE* mutant (Fig 4B).

Because the reversal frequency of the *frzZ* mutant was intermediate, we decided to use a more precise reversal-scoring test to determine unambiguously if *frzZ* mutant cells can still reverse in a Frz-dependent way. Our interpretation could be biased by so-called Tfp-dependent “stick-slip” motions, a Frz-independent Tfp-driven movement that could be mistakenly counted as reversals (S9A and S9B Fig, stick-slip motions are short range and generally appear distinct from *bona fide* reversals, [42]). Therefore, to score reversals with high accuracy, we monitored FrzS-YFP oscillations as a proxy for Frz-dependent reversals (Figs 4A, S9A and S9B). As expected from the reversal measurements, pole-to-pole switching of FrzS-YFP coincident with directional changes was reduced on average in the *frzZ* mutant, but they were still observed and sometimes up to WT levels (Fig 4C). Confirming this, pole-to-pole switching of FrzS-YFP was completely abolished in the *frzE* mutant and a *frzE frzZ* double mutant behaved like the *frzE* mutant (Fig 4C). Therefore, although the reduction in the reversal frequency of the *frzZ* mutant is significant (Fig 4B and 4C), Frz-dependent Tfp-reversals can occur in the absence of FrzZ (but not in the absence of FrzE) suggesting that FrzZ acts positively on Tfp-dependent reversals but is not strictly required for their activation.

To test the properties of the predicted core Frz pathway, we further constructed a *frzB*, *frzZ*, *aglZ* triple mutant (Δ3). The Δ3 mutant still showed Frz-dependent reversals and its reversal frequency was again lower, similar to that of the single *frzZ* mutant (Fig 4B). However, this lower reversal frequency did not translate into obvious S-motility developmental phenotypes because the Δ3 mutant formed fruiting bodies on soft agar, which is strictly S-motility dependent (Fig 4D). This multicellular development was Frz dependent because a Δ3 *frzE* quadruple mutant did not form aggregates in similar conditions (Fig 4D). On the contrary and as expected, the Δ3 mutant did not form aggregates on hard agar, a condition where A-motility is required (Fig 4D). We conclude that although the predicted core Frz pathway has a lower activity than the evolved pathway containing FrzZ, this activity could be sufficient to allow strictly S-motility-dependent behaviors, *ie* the ability to make fruiting bodies on soft 0.5% agar surfaces. Thus, the acquisition of FrzB, FrzZ and AglZ could have further adapted this primary circuit to the regulation of two motility machineries, at least in part by boosting the signaling activity (see below).

### RomR is the central output protein of the Frz pathway

The results above show that FrzE signaling is still efficient in the *frzZ* mutant (albeit at lower efficiency), suggesting that another response regulator delivers FrzE signals to the polarity complex. RomR is a possible candidate because it contains a phosphorylatable Nt-RR domain and because it interacts directly with MglA. Thus, phosphorylation of RomR could link Frz signaling to the polarity switch [34]. The regulatory function of the RomR protein could not be tested in the past because RomR is essential for A-motility (likely because MglA is delocalized in a *romR* mutant, [26,27,34]), and reversal frequencies are traditionally measured on A-motile cells. In the cellulose system, a *romR* deletion mutant showed WT Tfp-dependent motility (1.4 ± 0.8 μm.min^-1^, for 65 cells, S3 Movie) but it was dramatically affected in its ability to reverse in the FrzS-YFP oscillation assay (Fig 4C). A *frzZ romR* double mutant also had abolished reversals showing that RomR acts downstream from FrzZ in the regulation (Fig 4C). Importantly, while occasional reversals could be observed in *frzE* mutants, reversals were very rarely observed in *romR* and *frzE romR* mutants (Fig 4C). We conclude that RomR acts downstream from FrzE and FrzZ in the reversal pathway and could thus be a central output protein of the Frz pathway.

### The coordinate regulation of A- and S-motility requires an increase in steady-state Frz signaling activity, which depends on FrzZ

**Fig 5:**
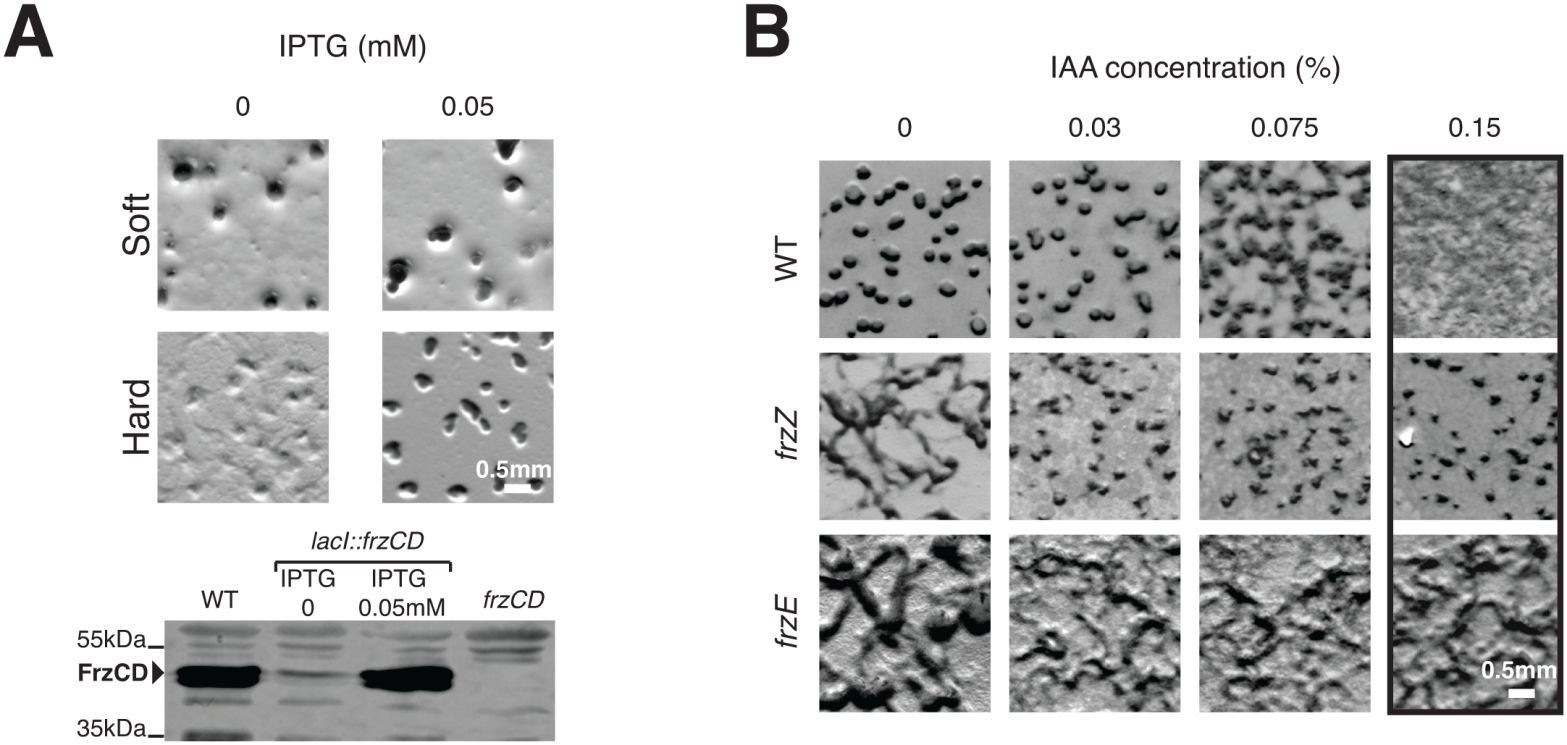
Increased Frz signaling activity is required for the regulation of A- and S-motility, which depends on FrzZ. **(A)** Weak Frz protein expression supports S-motility-dependent aggregation but not A- and S-motility-dependent aggregation. The ability of a strain in which the *frz* promoter has been replaced by an IPTG-inducible promoter to form aggregates is tested on soft (0.5%) or hard (1.5%) CF agar plates in the presence or in the absence of IPTG. A western blot analysis (bottom) of FrzCD expression in this strain reveals that promoter leakage is sufficient to support aggregation on the soft surface. **(B)** Extracellular complementation of the *frzZ* mutant developmental aggregation phenotype by IAA. Multicellular development on hard (1.5% agar) starvation CF medium containing increasing IAA concentrations by the WT strain and the *frzE* and *frzZ* mutants. Note that the *frzE* and *frzZ* mutants are indistinguishable in absence of IAA but that aggregation is only restored for the *frzZ* mutant in the presence of IAA

Both the *frzZ* and the *frzB frzZ aglZ* Δ3 mutants have similar and lower reversal frequencies than WT cells (Fig 4B), suggesting that the presence of FrzZ increases the steady-state signaling activity. Because the Δ3 mutant still forms fruiting bodies on 0.5% agar (Fig 4D), lower Frz activity may only translate in a developmental defect when A-motility is required. If so, differential Frz signaling activities may be required to regulate S-motility-dependent behaviors (ie development on soft agar) and A- and S-motility dependent behaviors (ie development on hard agar). This hypothesis can be tested in a strain expressing the *frz* operon under the control of an IPTG-inducible promoter (Fig 5A) where Frz activity should be related to the level of Frz protein expression. In the absence of IPTG, such strain formed fruiting bodies on 0.5% agar but not on 1.5% agar, indicating that promoter leakage is even sufficient to restore S-motility-dependent aggregation (Fig 5A). As expected, the addition of IPTG also restored fruiting body formation on 1.5% agar (Fig 5A). Thus, developmental processes that require the S-motility system require lower Frz activity levels than developmental processes that require A- and S-motility.

The FrzZ protein was thought to be central to Frz regulation because a *frzZ* mutant displays a typical *frz* phenotype on development hard agar [30]. However, if this phenotype is linked to lower Frz activity, it could be bypassed if Frz signaling is artificially increased in a *frzZ* mutant. To test this possibility, we took advantage of a chemical, Isoamyl alcohol (IAA) known to activate *frz*-dependent reversals. Although the exact target of IAA is not known, it appears to act on and de-methylate the FrzCD receptor (directly or indirectly, [43]) and its action is strictly Frz-dependent [31]. When added to hard developmental agar, IAA did not affect development of the WT strain up to concentrations of 0.075%, after which IAA blocked fruiting body formation (Fig 5B). A *frzE* mutant showed the typical *frz* phenotype and as expected, this phenotype was neither rescued nor modified by IAA addition (Fig 5B). Consistent with previous observations, the *frzZ* phenotype was indistinguishable from the *frzE* phenotype in absence of IAA (Fig 5B). However, and contrarily to the *frzE* mutant, IAA rescued aggregation of the *frzZ* mutant up to 0.15% IAA, a high dose that disrupts aggregation in WT cells (Fig 5B). Therefore, artificial activation of Frz signaling rescues the signaling defect of the *frzZ* mutant, suggesting that FrzZ acts to elevate Frz activity, allowing regulation of the two motility systems.

### FrzZ is required for optimal signal transmission

**Fig 6:**
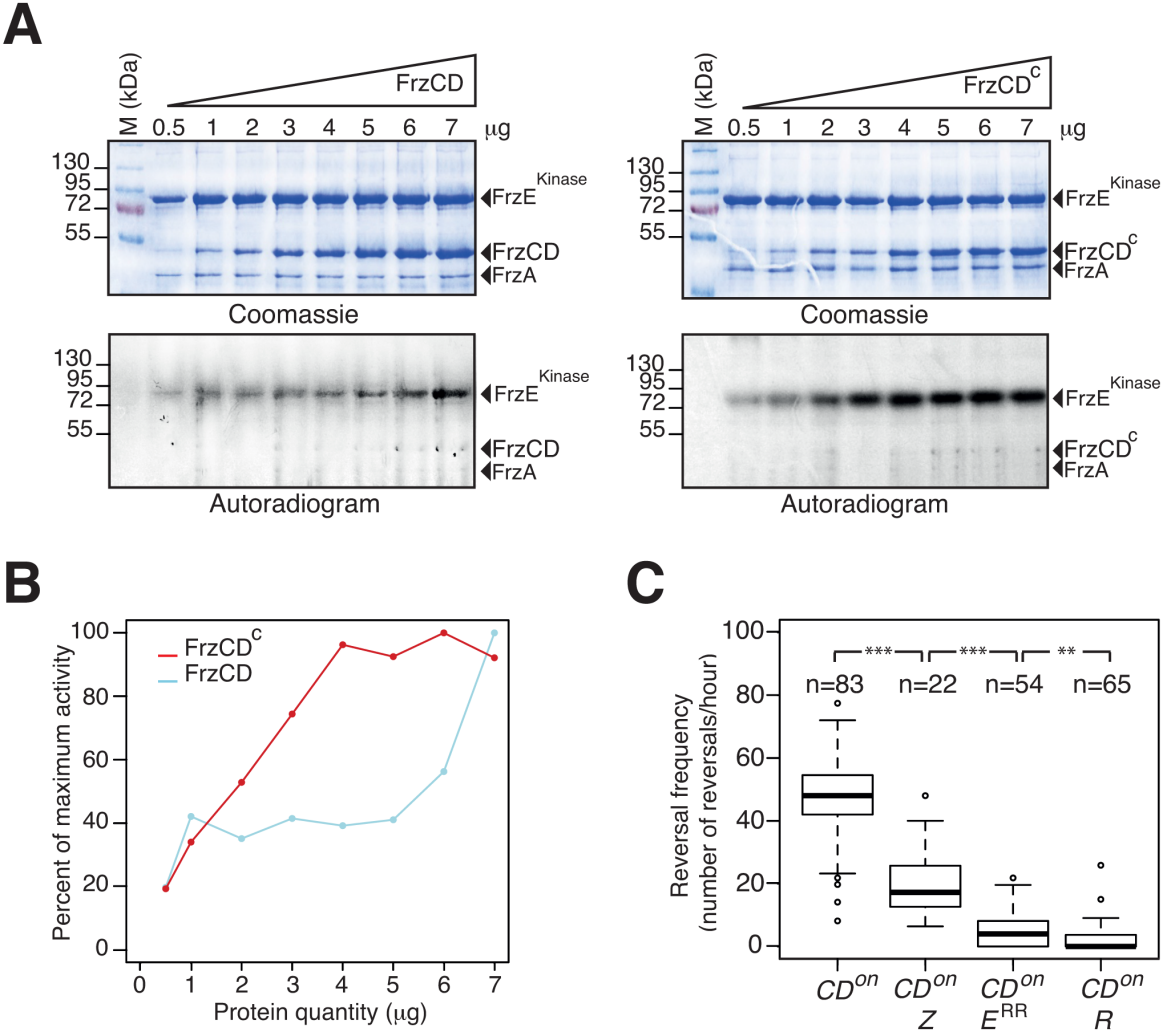
FrzZ is required for optimal transmission of Frz signals. **(A)** Auto-phosphorylation of the FrzE kinase domain (FrzE^kinase^) is stimulated by FrzCD^c^. The kinetics of FrzE^kinase^ auto-phosphorylation were tested *in vitro* by incubation of FrzE^kinase^ in the presence of FrzA, FrzCD or FrzCD^c^ and ATPγP^33^ as a phosphate donor. Note that FrzE^kinase^ auto-phosphorylation is stimulated at lower amounts of FrzCD^c^ than FrzCD. **(B)** Quantification of FrzE^kinase^ auto-phosphorylation by image densitometry of the autoradiograms shown in (A). **(C)** Signal transduction by FrzZ, FrzE^RR^ and RomR in FrzCD^c^-expressing strains (*frz* ^*on*^ mutant). Reversals were counted by automatic tracking in carboxymethylcellulose chambers in the absence of IAA (*frz* ^*on*^ mutants are blind to IAA). The boxplots read as in Fig 4B. CD^*on*^: *frz* ^*on*^; CD^*on*^ Z: *frz* ^*on*^ *frzZ*; CD^*on*^ E^RR^: *frz* ^*on*^ *frzE* ^*RR*^; CD^*on*^ R: *frz* ^*on*^ *romR*. Statistics: Wilcox test (n<40) for CD^*on*^ Z (*frz* ^*on*^ *frzZ*) and student test (t-test, n>40) for the other strains. ** p value<0.01, *** p value<0.0001.

To further investigate the function of FrzZ in Frz-signaling activity, we tested the contribution of FrzZ in a strain where the Frz receptor is hyper active. So-called *frz* ^*on*^ mutations map to the C-terminal domain of FrzCD and result in the expression of a truncated receptor protein (FrzCD^c^) [31]. Because the expression of FrzCD^c^ is trans-dominant to the expression of FrzCD, *frz* ^*on*^ mutations have been suggested to hyper activate Frz signaling [44]. To first test this assumption, we purified FrzCD, FrzCD^c^, FrzA and the kinase domain of FrzE (FrzE^kinase^, autophosphorylation of FrzE *in vitro* can only be detected if FrzE^RR^ is removed due to its phosphate sink activity, [32]) and compared the capacity of FrzCD and FrzCD^c^ to activate FrzE^kinase^ autophosphorylation from ATP *in vitro*. While both FrzCD and FrzCD^c^ were able to activate the autokinase activity of FrzE^kinase^ in a dose-dependent manner, FrzCD^c^ was a more potent activator (Fig 6A and 6B). Thus, *frz* ^*on*^ mutations induce a hyper signaling state of the FrzE kinase.

We then proceeded to test the contribution of FrzZ to Frz-signaling in a *frz* ^*on*^ background. In the cellulose chamber assay, *frz* ^*on*^ mutants reversed at high frequency, as expected (Fig 6C, compare with the WT reversal frequency in Fig 4B). Remarkably, a *frz* ^*on*^ *frzZ* mutant still reversed but at a reversal frequency similar to the reversal frequency of the *frzZ* mutant (Fig 6C, compare with Fig 4B). A *frz* ^*on*^ *romR* mutant did not reverse (Fig 6C), confirming that Frz signaling is disrupted in absence of RomR. Thus, FrzZ acts downstream from the FrzCD receptor and exerts a positive effect on the transduction of Frz signals to the polarity switch.

### FrzZ and FrzE^RR^ exert opposite effects on Frz signaling intensity

**Fig 7:**
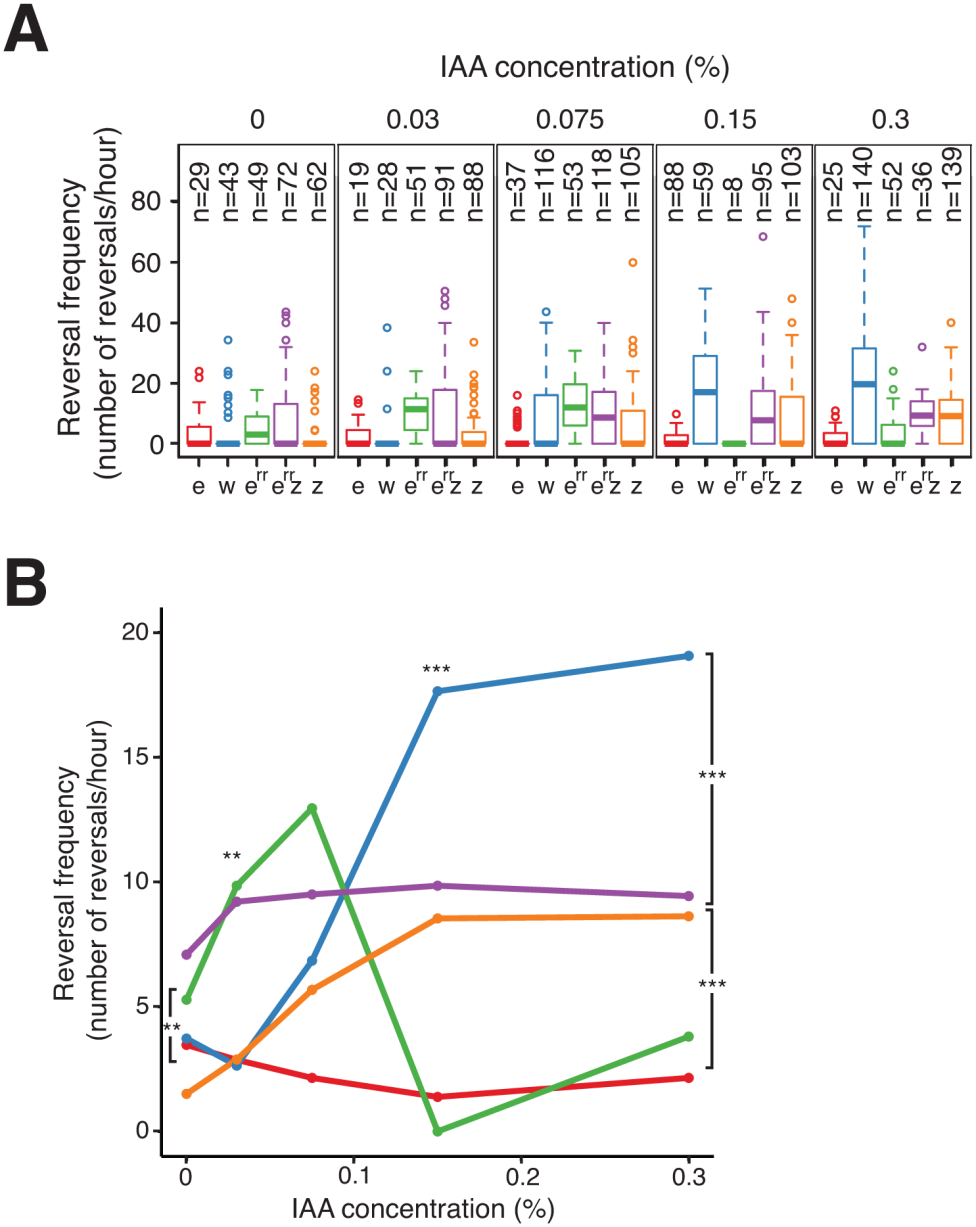
FrzE^RR^ inhibits and FrzZ amplifies Frz signaling independently. **(A)** S-motility-dependent response to increasing stimulation by WT and *frz* mutant cells. For each strain, the reversal frequency was measured in carboxymethylcellulose chambers by scoring FrzS-YFP pole-to-pole oscillations in the presence of increasing doses of IAA. The boxplots read as in Fig 4B. e: *frzE* (red); w: WT (blue); e^rr^: *frzE* ^*RR*^ (green); e^rr^ z: *frzE* ^*RR*^ *frzZ* (purple); z: *frzZ* (orange). **(B)** The average reversal frequencies compiled from (A) are shown as a function of IAA concentration for each tested strain. The same strain color code as in (A) applies. Statistics: Wilcox test. ** p value<0.01, *** p value<0.0001.

To investigate how FrzZ exerts its positive effect on Frz signaling activity, we took advantage of the cellulose system to develop a high-resolution single cell assay in which FrzS-YFP oscillations are measured directly as a function of stimulation levels; here, the addition of increasing doses of IAA. In this assay, we first established that reversals were induced by IAA in dose- and FrzE-dependent manners. In WT cells, IAA induced a sharp dose-dependent reversal response until a plateau was reached at an IAA concentration of 0.15% (Fig 7A and 7B). As expected, a *frzE* mutant only reversed occasionally whatever the IAA concentration, showing that the observed IAA effects are strictly Frz-dependent (Fig 7A and 7B). Consistent with previous results, a *frzZ* mutant still showed an IAA-dependent response but it was more gradual than the WT and showed lower amplitude at the higher IAA doses (Fig 7A and 7B).

We also used the IAA single cell assay to test the function of FrzE^RR^, the FrzE receiver domain. The FrzE^RR^ domain is not absolutely essential for Frz signaling and has been suggested to inhibit signaling because FrzE^RR^ inhibits FrzE autophosphorylation *in vitro* (Fig 1B, [32]). However, how such inhibition participates in Frz signaling is unclear. In the IAA assay, a *frzE* ^*RR*^ mutant showed a behavior opposite of that of the *frzZ* mutant: this mutant reversed more than WT cells which was apparent IAA doses ranging between 0–0.075% (Fig 7A and 7B). Thus, FrzE^RR^ prevents Frz-signaling at low stimulation levels. Remarkably, at concentrations higher than 0.075% the *frzE* ^*RR*^ mutant stopped reversing and no longer responded to IAA (Fig 7A and 7B). We hypothesize that this collapse is the result of an over-signaling state that disrupts Frz signaling function because (i), a *frzE* ^*RR*^ *frzZ* double mutant showed a composite phenotype: *frzE* ^*RR*^-type reversal frequencies at IAA doses ≤ 0.03% and *frzZ*-type reversal frequencies at the higher IAA concentrations (Fig 7A and 7B); and, (ii), a *frz* ^*on*^ *frzE* ^*RR*^ double mutant has a strongly reduced reversal frequency (Fig 6C). In the enteric Che pathway, high chemoreceptor stimulation also inhibits signaling suggesting that similar mechanisms are at work in the Frz system [45,46]

All together, the IAA experiments and the *frz* ^*on*^ mutant reversal frequencies suggest that FrzE^RR^ and FrzZ act independently in the pathway, FrzE^RR^ blocking activation at low signal levels and FrzZ amplifying signal transmission to allow a rapid switch-like response to stimulation (which is required for the regulation of A- and S-motility).

## Discussion

### Evolution of the *Myxococcus* motility apparatus

The *Myxococcus* motility apparatuses (A- and S-motility) allow this bacterium to perform an array of multicellular behaviors, which likely increases the competitiveness of this bacterium in the environment. Our results are consistent with an evolutionary scenario whereby these behaviors emerged following the stepwise assembly of four distinct functional modules, the Frz chemosensory apparatus, the polarity proteins MglAB, and the A- and S-motility systems, in a regulation pathway. Given that the studied genes likely form a minimal regulatory set and that not all players and interactions have been identified, a complete evolutionary scenario cannot be proposed. Nevertheless, we identify two major steps in the evolution of the pathway:

- In bacteria, the cooption of signaling modules formed by Mcp (FrzCD), CheW (FrzA), CheA (FrzE), CheR (FrzF) and CheB (FrzG) homologues underlies the emergence of a large number of chemosensory-type pathways [11]. Therefore, it is likely that the primary deltaproteobacterial S-motility regulation apparatus first evolved by recruitment of MglAB to one such chemosensory system. RomR is a possible candidate to link Frz signaling to polarity regulation because this protein shares a similar evolutionary history (Fig 3A and 3B), it interacts directly with MglA [26,27] and it is essential for reversals (this work). However, we have not demonstrated that FrzE is the RomR kinase and thus formally, other intermediate proteins may relay FrzE signals to RomR. Nevertheless, our genetic analysis suggests that RomR functions as a core protein downstream from FrzE in the regulation pathway. Downstream from MglA, it is also possible that FrzS does not constitute the only link to the S-motility apparatus [12,18,47], but because the interaction between MglA and FrzS is essential for S-motility [25], the acquisition of FrzS was probably a key step for the emergence of the primary pathway.
- Diversification of the primary pathway to the regulation of A- and S-motility occurred in the *Cystobacterinaea* family of bacteria and coincided with profound modifications of the regulation system. Additions of AglZ and FrzZ were probably key to adapt the primary circuit to the emergence of a branch point downstream from the FrzE kinase. First, the duplication of a *frzS* ancestor gene and connection of AglZ to the A-motility apparatus might have created the branch point itself, connecting A-motility to MglA regulation. Aside from numerous amino acid substitutions, the main difference between AglZ and FrzS resides in the lengths of their coiled-coil domains. Thus, AglZ might have evolved a new interaction with the Agl-Glt machinery (*ie* via the coiled-coil domain) while retaining its ability to interact with MglA. Consistent with this, AglZ also interacts with MglA and it co-localizes with the Agl-Glt machinery [16,25,39,48]. Second, FrzZ was incorporated into the upstream regulatory circuit, allowing signal partitioning to the two motility systems (see below). Other changes in the pathway not studied here may also participate in this regulation, including domain changes occurring in FrzCD and FrzG and the acquisition of FrzB (S4 Table and Figs 3A, 3B, S3A and S3E). The function of FrzB does not appear redundant to that of FrzA, the major Frz CheW protein, because Bustamante et al. [31] showed that a *frzA* mutant is indistinguishable from a *frzCD* or a *frzE* mutant, while a *frzB* mutant still responds to IAA stimulation in a bulk agar motility assay [31], which is consistent with our single cell experiments (Fig 4B). This lack of redundancy may not be surprising because FrzB is not phylogenetically related to FrzA (S3B and S3F Fig). It will be interesting to determine the exact function of FrzB and its potential connection to the branching of A-motility in the future.

### Architecture of the Frz pathway

**Fig 8:**
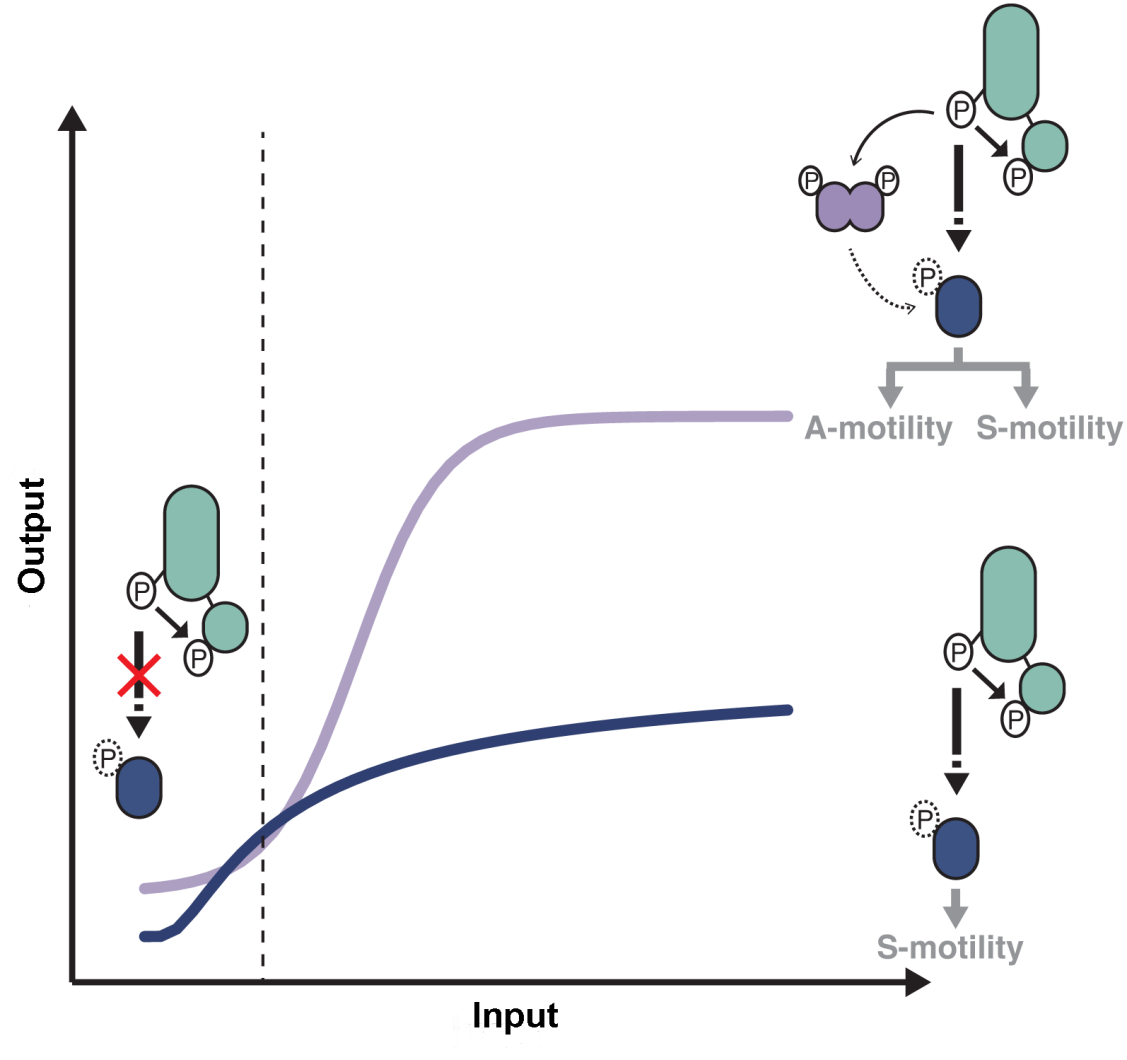
Antagonistic regulations by distinct RR domains shape Frz-dependent responses to stimulations. At low input levels, intramolecular phosphotransfer from the FrzE kinase domain (FrzE^kinase^) to the FrzE response regulator domain (FrzE^RR^) quenches the signal and prevents unwanted activation of reversals in absence of physiological signals. In the presence of activating signals (dotted line), the kinase activity of FrzE^kinase^ overcomes the phosphatase activity of FrzE^RR^ and the signal can be transduced downstream, presumably by the direct phosphorylation of RomR. In absence of FrzZ, signal transmission to the downstream motility machineries is not amplified (blue curve), only allowing S-motility-dependent regulations. In the presence of FrzZ, interactions between FrzE, FrzZ and RomR amplify the signaling activity allowing a steep and high amplitude response required to regulate both A- and S-motility (purple curve). Although the exact amplification mechanism remains to be determined the epistastic interactions between FrzE, FrzZ and RomR suggest that the FrzE kinase might activate reversals both by acting directly or indirectly on RomR and by direct phosphorylation of FrzZ, which would then interact with RomR to further activate it. The blue and purple curves are drawn from data shown in Fig 7B.

Using a high-resolution microfluidic single cell assay we were able to elucidate the individual contribution of the RR domain proteins of the pathway. The IAA stimulation Frz-dependent response curve showed a biphasic-type response with an overall sigmoidal shape (Fig 8). Because this response is entirely abolished in a *frzE* ^*RR*^ *frzZ* mutant but not in the respective individual mutants (Fig 7A and 7B), we conclude that FrzE^RR^ and FrzZ independently set distinct regimes of the signaling apparatus (Fig 8).

More precisely, FrzE^RR^ inhibits signaling at low stimulation levels, blocking noisy activation of the polarity switch (Fig 8). The inhibition mechanism is likely that of a phosphate sink because hybrid kinases phosphotransfer to their covalently-attached receiver domain at very high efficiency and the half-life of the Aspartate-phosphate bond on FrzE^RR^ is very short lived, a property of response regulators with phosphate sink functions [32,49,50]. Signal inhibition by the receiver domain of hybrid kinases is also emerging in other systems [51] and could be a widespread regulation of hybrid kinases.

At higher stimulation levels, the FrzE^RR^ inhibitory capacity becomes saturated and allows FrzE to phosphorylate the other receiver proteins of the pathway, FrzZ [30], possibly RomR or any other unidentified receiver domain of the pathway. The combined action of these phosphorylation events results in a steep response (Fig 8). Phosphorylation events downstream from FrzE and independent of FrzZ would transduce the signal to the MglAB proteins, this could occur through RomR or other uncharacterized regulators. In parallel, the phosphorylation of FrzZ amplifies the signal, impacting both the slope and amplitude of the response (Fig 8). Remarkably, processes that require S-motility alone can accommodate a graded response of moderate amplitude, while processes that require A- and S-motility require FrzZ amplification (Fig 8). It will be essential to determine how Frz signals are processed by MglA and each motility system to understand these signaling intensity requirements. At the molecular level, FrzZ must act between the FrzE kinase and RomR because (i), a hyper active FrzE kinase (*frz* ^*on*^ mutation) still requires FrzZ for maximal signal transmission (Fig 6C), suggesting that FrzZ does not exert its action by feedback stimulation of the autokinase activity of FrzE; and ii), a *frzZ romR* mutant behaves like a *romR* mutant (Fig 4C), showing clear epistatic relationships. FrzZ is a dual response regulator protein and it will be important to determine how the phosphorylation of each receiver domain contributes to signal amplification. The FrzZ phosphorylation sites appear partially redundant but only the phosphorylation of D52 and not D220 is important for the polar localization of FrzZ [33]. At the cell pole, the phosphorylated form of FrzZ could interact directly with RomR to facilitate the reversal switch.

Two-component cascades frequently employ accessory response regulator domains to achieve a variety of functions in phosphorelays, signal inhibition or negative feedback loops [52,53]. To our knowledge, this is the first time that a signal amplifier function is identified for a RR protein and because FrzZ-like CheY-CheY fusion proteins are predicted in other complex two component systems (Survey of the Microbial Signal Transduction database,[54]), characterizing the amplification mechanism may generally impact our understanding of bacterial signal transduction.

### Conclusions

In summary, this work reveals the modular structure of the Frz signal transduction pathway and suggests that the new pathway branch emerged at least in part by (i), gene duplication followed by new functional specialization (*ie* the emergence of AglZ and its connection to the A-motility system) and (ii), by the re-wiring of the signal flow, in this case an amplification system to partition signals at the branch point. These modifications are linked to the molecular structure of the *Myxococcus* regulation circuit where MglA acts as a regulation checkpoint, integrating upstream Frz signals into the coordinate regulation of the A- and S-motility machineries. In principle, amplification systems could operate in any signal transduction pathways that converge to the regulation of a checkpoint protein (also called a master regulator). For example and conceptually similar to the *Myxococcus* system, eukaryotic Ras-like G-proteins must also partition their activity to several output proteins, *ie* during chemotaxis [55]. While different circuit designs could have evolved to achieve this function, the *Myxococcus* system could provide valuable lens to study the evolutionary and mechanistic processes that allowed one such diversification.

## Materials and Methods

### Bacterial strains, plasmids, and growth

Strains, plasmids and primers used for this study are listed in S1, S2, and S3 Tables. In general, *M*. *xanthus* strains were grown at 32°C in CYE rich media as previously described [31]. Plasmids were introduced in *M*. *xanthus* by electroporation. Mutants and transformants were obtained by homologous recombination based on a previously reported method [31]. *E*. *coli* cells were grown under standard laboratory conditions in Luria-Bertani broth supplemented with antibiotics, if necessary.

### Phenotypic assays

Unless otherwise specified, soft and hard agar motility and development assays were performed as previously described [31]. In general, cell were grown up to an OD = 0.5 and concentrated ten times before they were spotted (10μL) on CYE or CF [31] 0.5% (soft) agar or 1.5% (hard) agar plates for motility or for developmental assays. Colonies were photographed after 48 H or 72 H for motility or development, respectively. Developmental assays in the presence of Isoamyl alcohol (IAA, Sigma Aldrich) were performed similarly except that plates also contained IAA at appropriate concentrations.

### Single cell motility assays

Single cell Tfp-dependent motility assays were initially developed by Sun et al. [13] in a system where *Myxococcus* cells are overlaid in methylcellulose. However, in this assay, reversals as observed on agar are not observed. In this media, the cells are loosely attached to the glass surface and they systematically detach and become tethered by one cell pole before a directional change is observed [13]. Many of these events may well result from actual reversal events, but other Tfp-dependent motions have been observed including stick-slip motions, sling-shot motions and walking up-right [42,56,57]. To avoid confusion linked to complex Tfp-dependent movements, we sought to optimize the methylcellulose assay. For this, homemade PDMS glass microfluidic chambers [58] were treated with 0.015% carboxymethylcellulose after extensive washing of the glass slide with water. For each experiment, 1mL of a CYE grown culture of OD = 0.5–1 was injected directly into the chamber and the cells were allowed to settle for 5 min. Motility was assayed after the chamber was washed with TPM 1mM CaCl_2_ buffer [58]. For IAA injections, IAA solutions made in TPM 1mM CaCl_2_ buffer at appropriate concentrations were injected directly into the channels and motility was assayed directly under the microscope. In TPM 1mM CaCl_2_, we found that most WT motile cells left the field of view before reversing, making statistically reliable measurements of reversal frequencies difficult. This low reversal frequency is due to the absence of stimulating signals in these conditions. To increase the reversals counts and unless otherwise stated, Frz signaling was stimulated by adding 0.1–0.15% IAA to the TPM mix. Time-lapse experiments were performed as previously described (Ducret et al., 2013) using a Nikon TE2000-E-PFS inverted epifluorescence microscope.

### Automated cell tracking and statistics

Image analysis was performed with a specific library of functions written in Python and adapted from available plugins in FIJI/ImageJ [59]. Cells were detected by thresholding the phase contrast images after stabilization. Cell tracking was obtained by calculating all objects distances between two consecutive frames, thus selecting the nearest objects. The computed trajectories were systematically verified manually and when errors were encountered, the trajectories were removed. The analysis of the trajectories is done automatically by a Python script that calculates the angle formed by the segments between the center of the cell at time t, the center of the cell at time t-1 and the center at time t+1. Directional changes were scored as reversals when cells switched their direction of movement and the angle between segments was less than 90°. For non-reversing strains, the number of reversals for each cells was plotted against time using R software (http://www.R-project.org/). For strains that frequently reversed, the mean time between two reversals for each cells was plotted against time using R software.

To further discriminate *bona fide* reversal events from stick-slip motions, the fluorescence intensity of FrzS-YFP was measured at cell poles over time. For each cell that was tracked, the fluorescence intensity and reversal profiles were correlated to distinguish *bona fide* reversals from stick-slip events with the R software. When a directional change was not correlated to a switch in fluorescence intensity, this change was discarded as a stick-slip event. The number of reversals was plotted against time using R software.

Statistics were done using R software: Wilcox test was used when the number of cells was less than 40 in at least one of the two populations compared, and student test (t-test) was used for a number of cells higher than 40.

### Cloning, expression and purification of *M*. *xanthus* Frz system proteins

The genes encoding FrzE^kinase^, FrzA, FrzCD and FrzCD^c^ were amplified by PCR using *M*. *xanthus* DZ2 chromosomal DNA as template and the forward and reverse primers listed in S3 Table. The amplified product was digested with the appropriate restriction enzymes, and ligated either into the pETPhos or pGEX plasmids generating pETPhos_*frzE* ^*kinase*^, pETPhos_*frzCD*, pETPhos_*frzCDc* and pGEX_*frzA* which were used to transform *E*. *coli* BL21(DE3)Star cells in order to overexpress His-tagged or GST-tagged proteins. All constructs were verified by DNA sequencing. Recombinant strains harboring the different constructs were used to inoculate 400 ml of LB medium supplemented with glucose (1mg/mL) and ampicillin (100μg/ml), and the resulting cultures were incubated at 25°C with shaking until the optical density of the culture reached an OD = 0.6. IPTG (0.5 mM final) was added to induce the overexpression, and growth was continued for 3 extra hours at 25°C. Purification of the His-tagged/GST-tagged recombinant proteins was performed as described by the manufacturer (Clontech/GE Healthcare).

### *In vitro* autophosphorylation assay

*In vitro* phosphorylation assay was performed with *E*. *coli* purified recombinant proteins. 4 μg of FrzE^kinase^ were incubated with 1μg of FrzA and increasing concentrations (0.5 to 7μg) of either FrzCD or FrzCD^c^ in 25 μl of buffer P (50 mM Tris-HCl, pH 7.5; 1 mM DTT; 5 mM MgCl_2_; 50mM KCl; 5 mM EDTA; 50μM ATP, 10% glycerol) supplemented with 200 μCi ml^-1^ (65 nM) of [γ-33P]ATP (PerkinElmer, 3000 Ci mmol^-1^) for 10 minutes at room temperature in order to obtain the optimal FrzE^kinase^ autophosphorylation activity. Each reaction mixture was stopped by addition of 5 × Laemmli and quickly loaded onto SDS-PAGE gel. After electrophoresis, proteins were revealed using Coomassie Brilliant Blue before gel drying. Radioactive proteins were visualized by autoradiography using direct exposure to film (Carestream).

### Inducible expression of FrzCD by IPTG

669 bp upstream from *frzCD* were amplified with primers CDind1 (gaattcATGTCCCTGGACACCCCCAACGA) and CDind2 (actagtCATGGCCTGGATGAACTCGCCAAT) and cloned into pGEM T-easy (Promega) to obtain plasmid pEM140. pEM140 was digested with SpeI and EcoRI and the excised DNA fragment was cloned into pLacI (a derivative of pAK20 [60]) previously digested with the same enzymes. The resulting plasmid, pEM143, is a derivative of pBBR1MCS carrying the *lacI* gene under its promoter and followed by the first 669 of *frzCD* and thus its integration by homologous recombination places the entire *frz* operon under IPTG control.

Developmental plate assays were conducted in the presence (0.5 mM) or absence of IPTG. For western blotting, strains were grown overnight with or without appropriate concentrations of IPTG. The cultures were concentrated to OD = 4 and western blotting was performed as previously described with 1/10,000 dilutions of anti-FrzCD (Bustamante et al., 2004).

### Genomic dataset construction

A local protein database containing the 2,316 complete prokaryotic proteomes available in the NCBI (http://www.ncbi.nlm.nih.gov/) as of May 23, 2013 was built. This database was queried with the BlastP program (default parameters, [61]) using the full length sequences of the signaling proteins (FrzF (MXAN_4138), FrzG (MXAN_4139), FrzE (MXAN_4140), FrzCD (MXAN_4141), FrzB (MXAN_4142), FrzA (MXAN_4143) and FrzZ (MXAN_4144)), the polarity control proteins (MglA (MXAN_1925), MglB (MXAN_1926) and RomR (MXAN_4461)) and the downstream proteins (FrzS (MXAN_4149) and AglZ (MXAN_2991)) of *M*. *xanthus* as a seed. The homology was assessed by visual inspection of each BlastP output (no arbitrary cut-offs on the E-value or score). The retrieved sequences were aligned using MAFFT version 7 (default parameters, [62]). Regions where the homology between amino acid positions was doubtful were removed using the BMGE software (BLOSUM30 option; [63]).

For each protein, preliminary phylogenetic analyses were performed using FastTree v.2 using a gamma distribution with four categories [64]. Most of the studied proteins belong to very large protein families. Based on the resulting trees, the subfamilies containing the sequences from *M*. *xanthus* were identified and selected for further phylogenetic investigations. The corresponding sequences were realigned using MAFFT version 7 with the linsi option, which ensures accurate alignments. The resulting alignments were trimmed with BMGE as previously described.

### Phylogenetic analyses

Maximum likelihood (ML) trees were computed using PHYML version 3.1 [65] with the Le and Gascuel (LG) model (amino acid frequencies estimated from the dataset) and a gamma distribution (4 discrete categories of sites and an estimated alpha parameter) to take into account evolutionary rate variations across sites. Branch robustness was estimated by the non-parametric bootstrap procedure implemented in PhyML (100 replicates of the original dataset with the same parameters). Bayesian inferences (BI) were performed using Mrbayes 3.2.2 [66] with a mixed model of amino acid substitution including a gamma distribution (4 discrete categories). MrBayes was run with four chains for 1 million generations and trees were sampled every 100 generations. To construct the consensus tree, the first 2000 trees were discarded as “burn in”.

The phylogenetic signal can be substantially increased by combining multiple sequence alignments of proteins involved in the same cellular function/biological process and sharing a common evolutionary history in a single large alignment (also called supermatrix), [16,67–71]. Among the 12 studied genes, we showed that FrzF, FrzG, FrzCD and FrzE are always clustered together in genomes and share a similar evolutionary history. These genes were thus combined to build a supermatrix (S4 Fig). For similar reasons, a second supermatrix was built by combining MglA and MglB (S2 Fig). The ML and BI phylogenetic trees corresponding to these two supermatrices were inferred as previously described [16].

### Reference phylogeny of Delta/Epsilonproteobacteria

The 79 complete proteomes of Delta/Epsilonproteobacteria available at the NCBI in May 17, 2013 were retrieved and assembled in a local database (S5 Table). We used SILIX to build the protein families of homologous sequences present in these genomes (default parameters; [72]).The homologous sequences corresponding to protein families present exactly in a single copy per genome (17 proteins) were aligned using MAFFT Version 7 (linsi option), trimmed with BMGE and combined to build a large supermatrix (6958 positions). The ML phylogenetic tree corresponding to this large supermatrix was inferred with PhyML, as described above.

## Supporting Information

S1 Fig Individual phylogenies of the polarity control proteins. Shown are unrooted Bayesian phylogenetic trees for **(A)** MglA (MXAN_1925, 215 sequences, 118 positions), Group 1 MglA are represented in the lower part of the tree **(B)** MglB (MXAN_1926, 90 sequences, 110 positions) and **(C)** RomR (MXAN_4461, 30 sequences, 206 positions). Numbers at nodes indicate posterior probabilities (PP) computed by MrBayes and bootstrap values (BV) computed by PhyML. Only PP and BV above 0.5 and 50% are shown. The scale bars represent the average number of substitutions per site. In the phylogenetic tree MglA, MglB and RomR proteins from *M*. *xanthus* are illustrated with color-coded gene symbols.
(PDF)

S2 Fig Phylogenetic trees of concatenated alignments of MglA and MglB. Shown is a rooted Bayesian phylogenetic tree (79 sequences, 248 positions). The root has been placed according to the phylogenies of the individual proteins. Numbers at nodes indicate posterior probabilities (PP) computed by MrBayes and bootstrap values (BV) computed by PhyML. Only PP and BV above 0.5 and 50% are shown. The scale bars represent the average number of substitutions per site. In the phylogenetic tree the concatenated MglA/B proteins from *M*. *xanthus* are illustrated with color-coded gene symbols. For each species the individual locus_tags of the concatenated proteins are indicated in brackets.
(PDF)

S3 Fig Individual phylogenies of the Frz proteins. Shown are unrooted Bayesian phylogenetic trees of the signaling proteins: **(A)** FrzCD (MXAN_4141, 191 sequences, 288 positions), **(B)** FrzA (MXAN_4143, 98 sequences and 84 positions), **(C)** FrzE (MXAN_4140, 144 sequences, 482 positions), **(D)** FrzF (MXAN_4138, 104 sequences, 276 positions), **(E)** FrzG (MXAN_4139, 132 sequences, 283 positions), **(F)** FrzB (MXAN_4142, 11 sequences, 113 positions) and **(G)** FrzZ (MXAN_4144, 64 sequences, 102 positions). Number at nodes indicates posterior probabilities (PP) and bootstrap support (BS) computed by Mrbayes and PhyMl, respectively. Only posterior probabilities and bootstrap values greater, respectively, than 0.5 and 50% are shown. The scale bars represent the number of substitutions per site. In each phylogenetic tree Frz proteins from *M*. *xanthus* are illustrated with color-coded gene symbols The domain composition is illustrated in the right.
(PDF)

S4 Fig Phylogenetic trees of concatenated alignments of FrzF, FrzG, FrzE and FrzCD. Shown is a rooted Bayesian phylogenetic tree (134 sequences, 1294 positions). The root has been placed according to the phylogenies of the individual proteins. Numbers at nodes indicate posterior probabilities (PP) computed by MrBayes and bootstrap values (BV) computed by PhyML. Only PP and BV above 0.5 and 50% are shown. The scale bars represent the average number of substitutions per site. In the phylogenetic tree the concatenated FrzF-G-E-CD proteins from *M*. *xanthus* are illustrated with color-coded gene symbols. For each species the individual locus_tags of the concatenated proteins are indicated in brackets. FrzA homologues were not added to the concatenated dataset because there is often more than one FrzA-like protein per system and therefore, the evolutionary relationships between these different copies cannot be determined with confidence.
(PDF)

S5 Fig Genetic organization of core *frz* genes in selected genomes. The same color code as in Fig 1 is used. Locus_tags are shown for all genes. White arrows indicate genes encoding for proteins that are not related to the Frz system or motility. The *frzZ* homologues containing only one response regulator domains are indicated with an asterisk.
(PDF)

S6 Fig FrzS and AglZ derive from the duplication of a FrzS ancestor gene. Shown is an unrooted Bayesian phylogenetic tree of AglZ/FrzS (MXAN_2991/MXAN_4149, 26 sequences, 467 positions). Numbers at nodes indicate posterior probabilities (PP) computed by MrBayes and bootstrap values (BV) computed by PhyML. Only PP and BV above 0.5 and 50% are shown. The scale bars represent the average number of substitutions per site. In the phylogenetic tree FrzS and AglZ proteins from *M*. *xanthus* are illustrated with color-coded gene symbols.
(PDF)

S7 Fig FrzS and AglZ response regulator domain alignment. The positions of the phosphorylation site (D57 in the canonical CheY domain), the residues that chelates the Mg^2+^necessary for aspartic acid phosphorylation (D12 and D13 in the canonical CheY domain) and the residues involved in a shift of the hydrogen-bonding network (T87, Y106 and K109 in the canonical CheY domain) are indicated in red, grey and black, respectively.
(PDF)

S8 Fig Tfp-dependent motility and single cell tracking in carboxymethylcellulose-coated glass microfluidic chambers. **(A)** Single cell Tfp-dependent reversal. Shown is a WT cell. Time frame: 15s, scale bar = 2μm. **(B)** Tracking of Tfp-dependent reversals. Selected timelapses are subjected to automated cell detection and tracking using a homegrown procedure under Fiji (Image J/NIH). For each cell the algorithm records the velocity (green), the cumulated traveled distance (blue) and count reversals (red), which is used to calculate the reversal frequency.
(PDF)

S9 Fig Discriminating regulated reversals from stick-slip motions by monitoring FrzS-YFP oscillations in reversing cells. **(A)** Time-lapse of a single cell expressing FrzS-YFP showing both reversals and stick-slip motions. Note that regulated reversals (R) are accompanied by pole-to-pole switching of FrzS while stick-slip motions (S) are not correlated with changes in FrzS fluorescence at the cell poles. Time frame = 15s. Scale bar = 2 μm. **(B)** Scoring reversals by single cell tracking of FrzS-YFP fluctuations. Shown is the fluorescence intensity profile over time of the cell shown in (A). Blue line, fluorescence intensity at the initial leading pole. Red line, fluorescence intensity at the initial lagging pole. For each cell that was tracked, a reversal was counted when a directional change correlated to a FrzS-YFP fluorescence intensity switch (black line, when the blue and red lines cross). Stick-slip motions (S) or any other motions that were not correlated to fluorescence switches were systematically discarded in the counts.
(PDF)

S1 Movie *aglQ* mutant motility on carboxymethylcellulose. Movie (15 frames per second) of an A^−^ (*aglQ*) mutant on carboxymethylcellulose assay in a microfluidic chamber under 40X objective in the presence of IAA 0.15%. The time frame is 15 s for a total time of 30 min.
(AVI)

S2 Movie WT strain on carboxymethylcellulose: composite movie showing cell automated tracking. Composite movie (15 frames per second) of a WT strain on carboxymethylcellulose assay in a microfluidic chamber under 40X objective in the presence of IAA 0.15%. The time frame is 15 s for a total time of 30 min. The composite represents the movie without treatment (left) and with cell traces (right) generated by the automated cell tracking. Cells were tracked only when they stayed in the field of view for at least 10 min.
(AVI)

S3 Movie *romR* mutant motility on carboxymethylcellulose. Movie (15 frames per second) of a *romR* mutant on carboxymethylcellulose assay in a microfluidic chamber under 40X objective. The time frame is 15 s for a total time of 30 min.
(AVI)

S1 Table Bacterial strains used in this study. (DOCX)

S2 Table Plasmids used in this study. (DOCX)

S3 Table Primers used in this study. (DOCX)

S4 Table Protein domains in FrzCD, FrzE, FrzG, FrzF and FrzS homologues. (PDF)

S5 Table Genomes used to construct the tree of the Deltaproteobacteria. (DOCX)

S1 Text Supporting references. (DOCX)
