# Supplementary figures and images for: Evolution and Design Governing Signal Precision and Amplification in a Bacterial Chemosensory Pathway
Source: PLoS Genet. 2015 Aug 20;11(8):e1005460. doi: 10.1371/journal.pgen.1005460 (PMC4546325; doi:10.1371/journal.pgen.1005460)

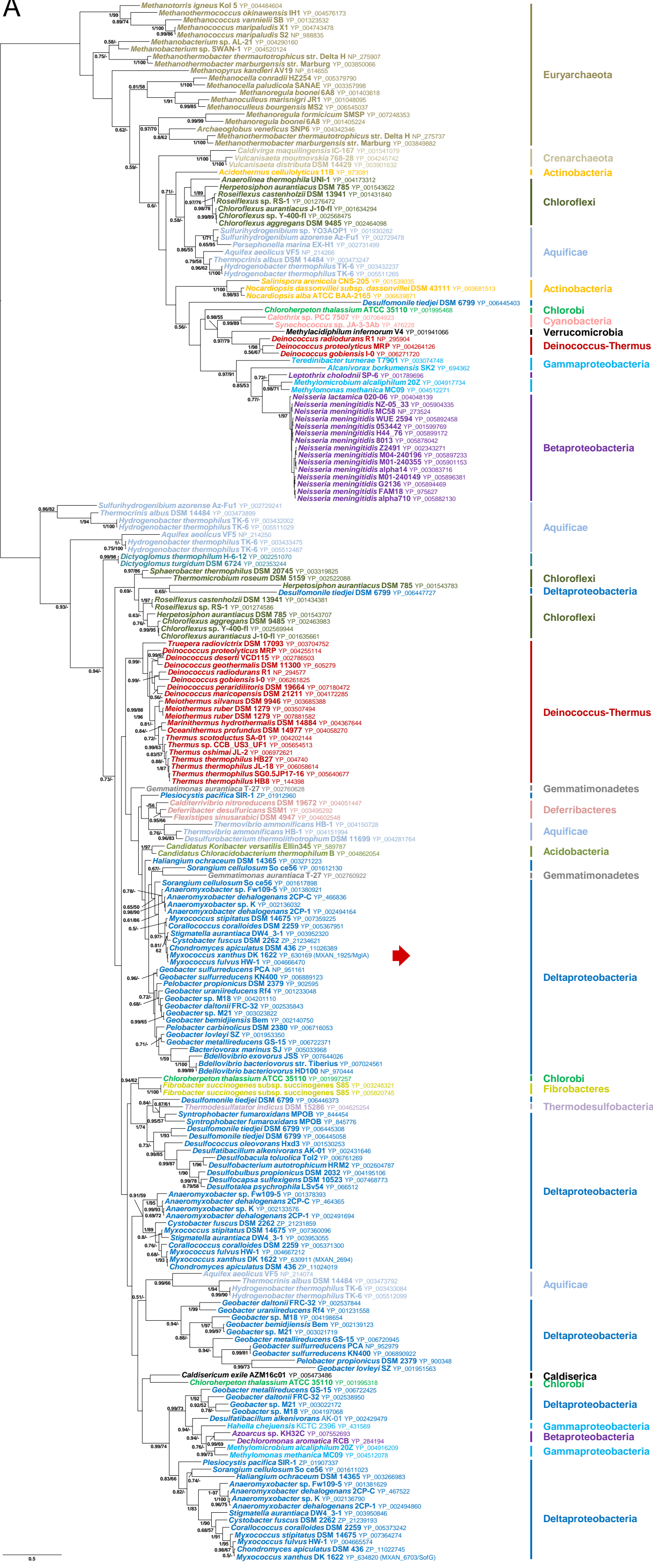

B

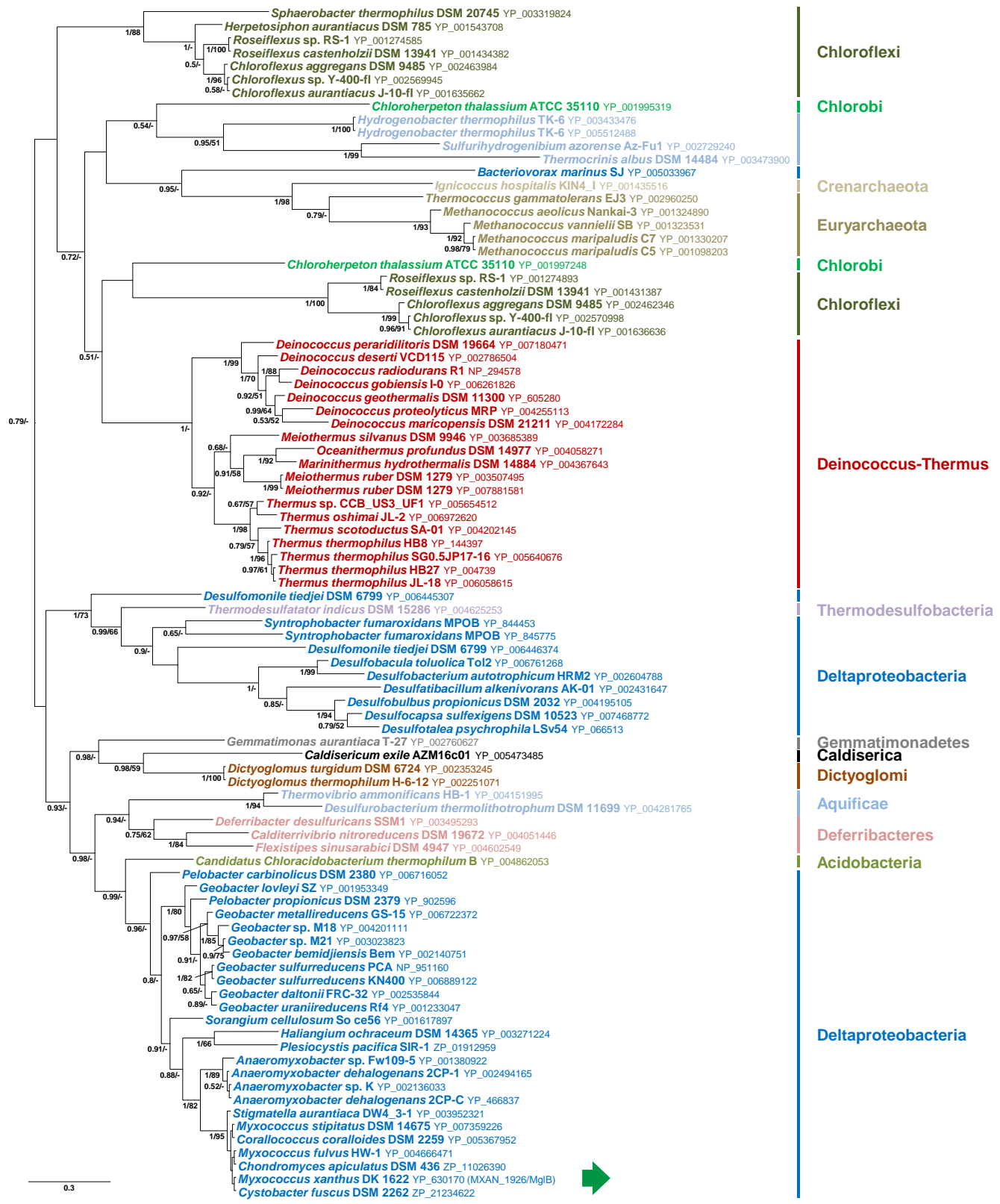

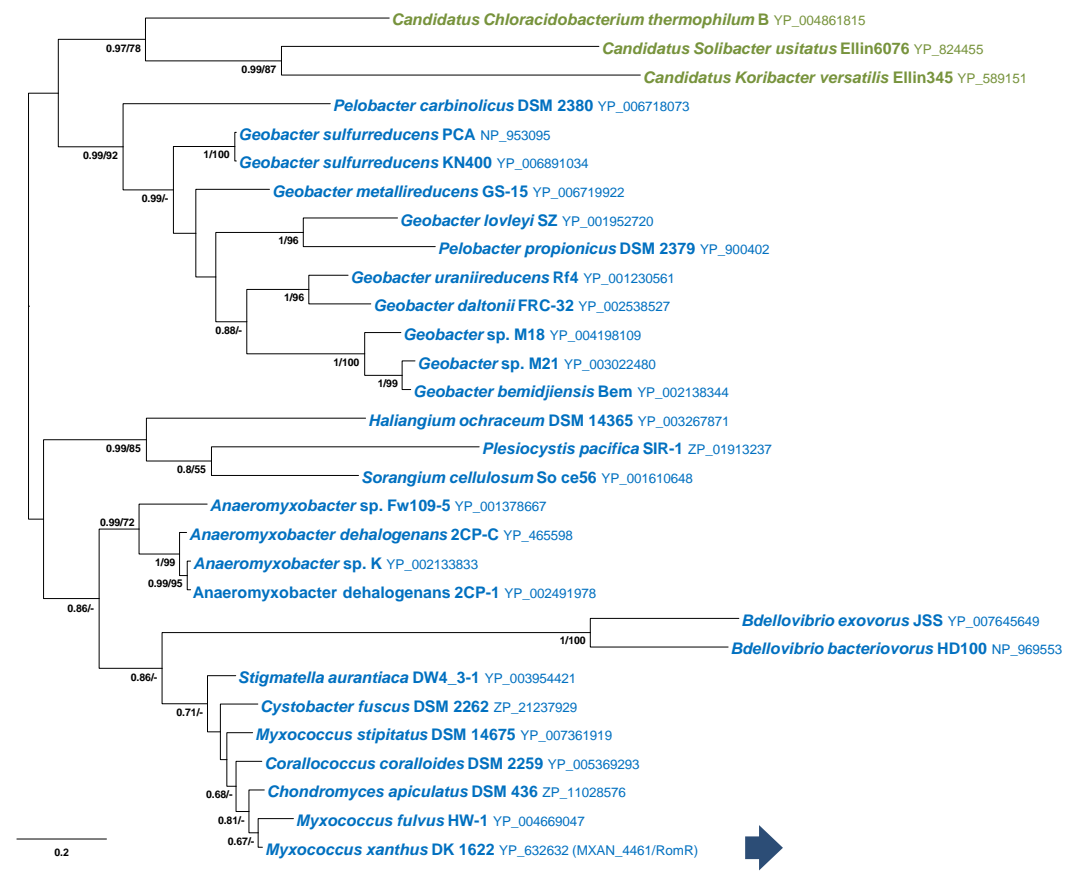

Acidobacteria

Deltaproteobacteria

Supplement: S1 Fig — Shown are unrooted Bayesian phylogenetic trees for (A) MglA (MXAN_1925, 215 sequences, 118 positions), Group 1 MglA are represented in the lower part of the tree (B) MglB (MXAN_1926, 90 sequences, 110 positions) and (C) RomR (MXAN_4461, 30 sequences, 206 positions). Numbers at nodes indicate posterior probabilities (PP) computed by MrBayes and bootstrap values (BV) computed by PhyML. Only PP and BV above 0.5 and 50% are shown. The scale bars represent the average number of substitutions per site. In the phylogenetic tree MglA, MglB and RomR proteins from M. xanthus are illustrated with color-coded gene symbols. (PDF) [file pgen.1005460.s001.pdf]

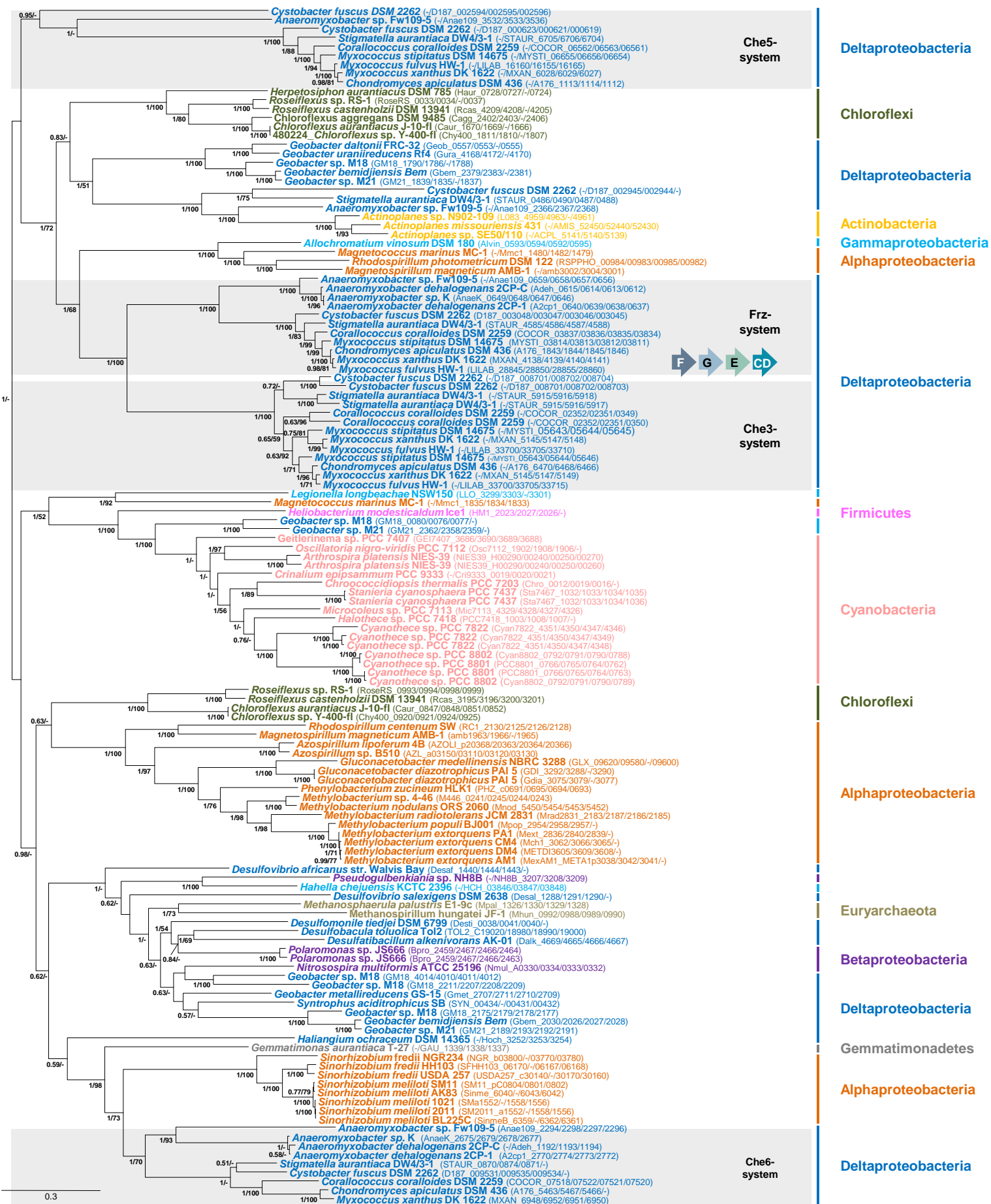

Supplement: S4 Fig — Shown is a rooted Bayesian phylogenetic tree (134 sequences, 1294 positions). The root has been placed according to the phylogenies of the individual proteins. Numbers at nodes indicate posterior probabilities (PP) computed by MrBayes and bootstrap values (BV) computed by PhyML. Only PP and BV above 0.5 and 50% are shown. The scale bars represent the average number of substitutions per site. In the phylogenetic tree the concatenated FrzF-G-E-CD proteins from M. xanthus are illustrated with color-coded gene symbols. For each species the individual locus_tags of the concatenated proteins are indicated in brackets. FrzA homologues were not added to the concatenated dataset because there is often more than one FrzA-like protein per system and therefore, the evolutionary relationships between these different copies cannot be determined with confidence. (PDF) [file pgen.1005460.s004.pdf]

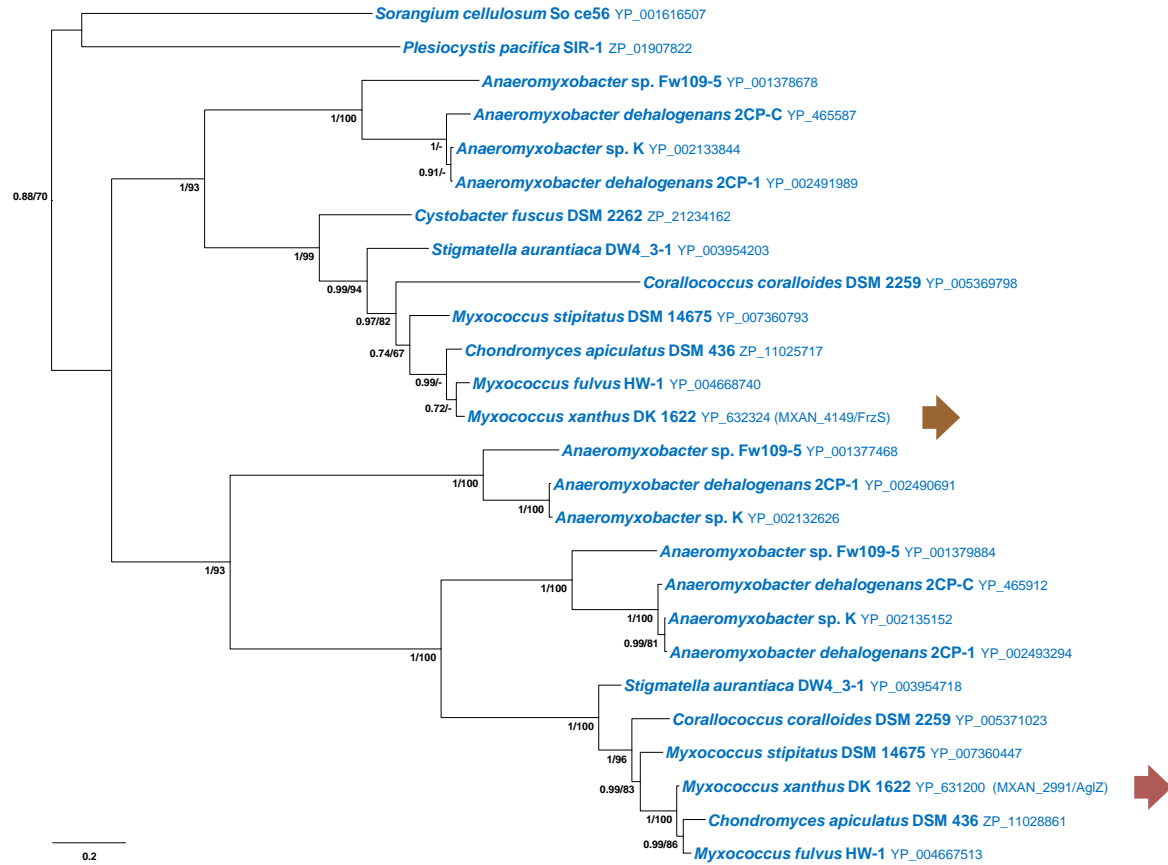

Deltaproteobacteria

Supplement: S6 Fig — Shown is an unrooted Bayesian phylogenetic tree of AglZ/FrzS (MXAN_2991/MXAN_4149, 26 sequences, 467 positions). Numbers at nodes indicate posterior probabilities (PP) computed by MrBayes and bootstrap values (BV) computed by PhyML. Only PP and BV above 0.5 and 50% are shown. The scale bars represent the average number of substitutions per site. In the phylogenetic tree FrzS and AglZ proteins from M. xanthus are illustrated with color-coded gene symbols. (PDF) [file pgen.1005460.s006.pdf]

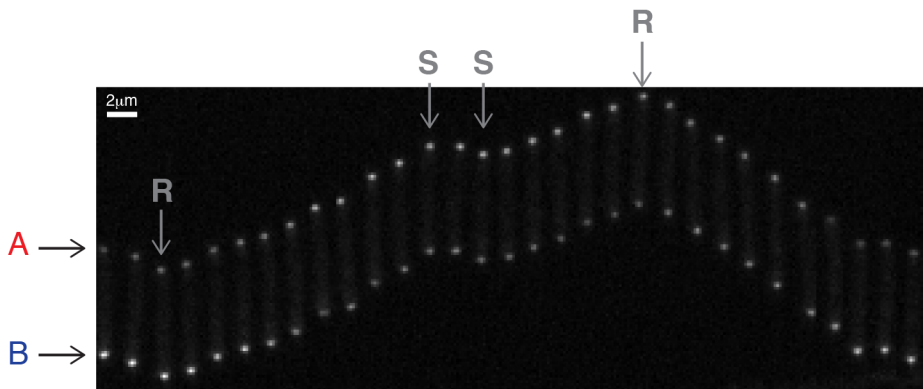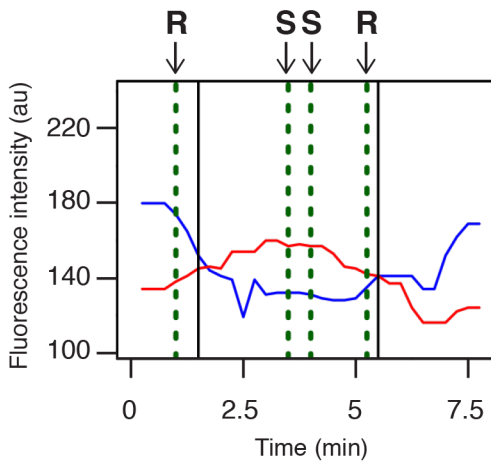

Supplement: S9 Fig — (A) Time-lapse of a single cell expressing FrzS-YFP showing both reversals and stick-slip motions. Note that regulated reversals (R) are accompanied by pole-to-pole switching of FrzS while stick-slip motions (S) are not correlated with changes in FrzS fluorescence at the cell poles. Time frame = 15s. Scale bar = 2 μm. (B) Scoring reversals by single cell tracking of FrzS-YFP fluctuations. Shown is the fluorescence intensity profile over time of the cell shown in (A). Blue line, fluorescence intensity at the initial leading pole. Red line, fluorescence intensity at the initial lagging pole. For each cell that was tracked, a reversal was counted when a directional change correlated to a FrzS-YFP fluorescence intensity switch (black line, when the blue and red lines cross). Stick-slip motions (S) or any other motions that were not correlated to fluorescence switches were systematically discarded in the counts. (PDF) [file pgen.1005460.s009.pdf]
