# Supplementary material for: Evolution and Design Governing Signal Precision and Amplification in a Bacterial Chemosensory Pathway
Source: PLoS Genet. 2015 Aug 20;11(8):e1005460. doi: 10.1371/journal.pgen.1005460 (PMC4546325; doi:10.1371/journal.pgen.1005460)
Supplement: S2 Fig — Shown is a rooted Bayesian phylogenetic tree (79 sequences, 248 positions). The root has been placed according to the phylogenies of the individual proteins. Numbers at nodes indicate posterior probabilities (PP) computed by MrBayes and bootstrap values (BV) computed by PhyML. Only PP and BV above 0.5 and 50% are shown. The scale bars represent the average number of substitutions per site. In the phylogenetic tree the concatenated MglA/B proteins from M. xanthus are illustrated with color-coded gene symbols. For each species the individual locus_tags of the concatenated proteins are indicated in brackets. (PDF) [file pgen.1005460.s002.pdf]

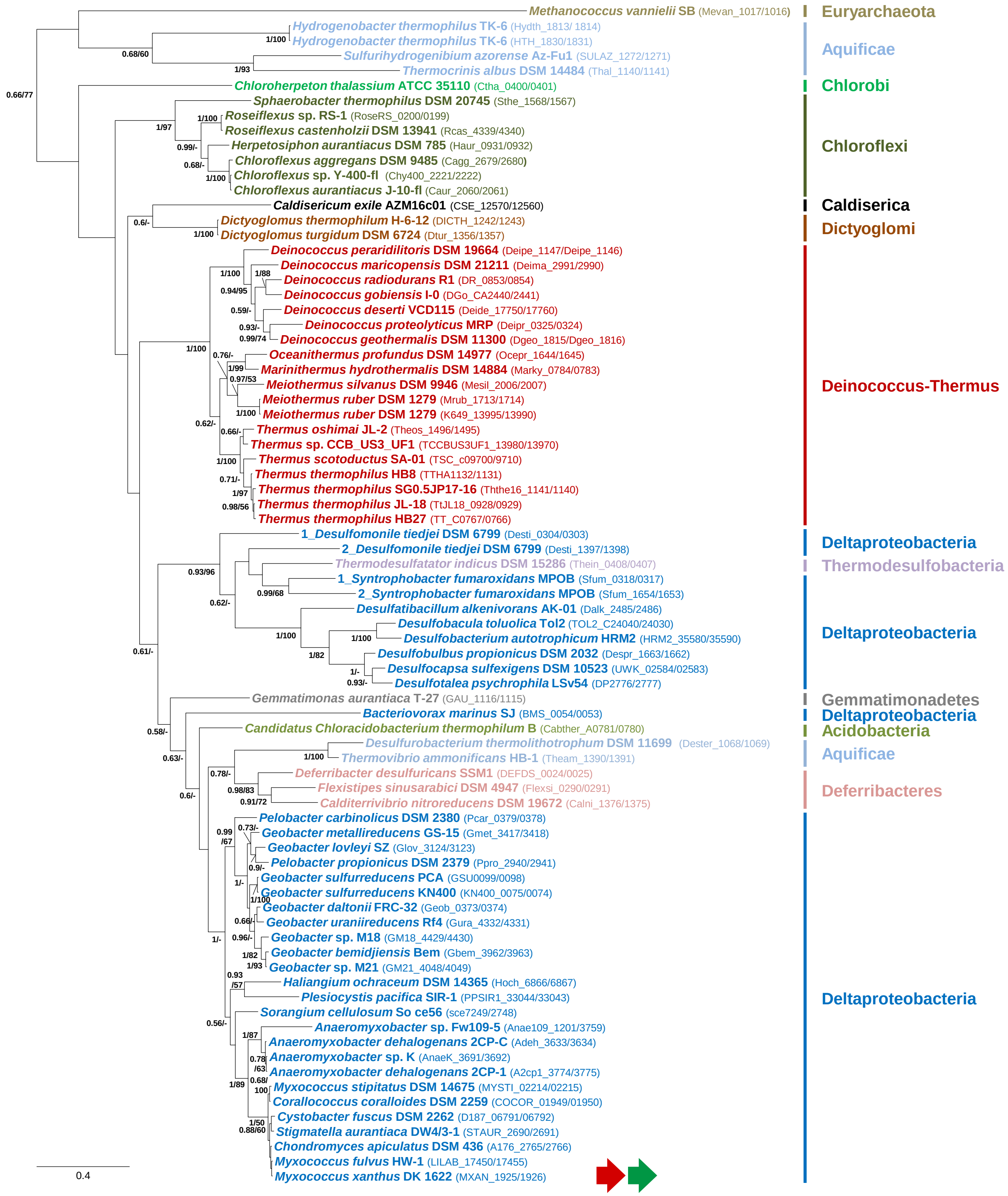

Methanococcus vannielii SB (Mevan_1017/1016)
Hydrogenobacter thermophilus TK-6 (Hydth_1813/ 1814)
Hydrogenobacter thermophilus TK-6 (HTH_1830/1831)
Sulfurihydrogenibium azorense Az-Fu1 (SULAZ_1272/1271)
Thermocrinis albus DSM 14484 (Thal_1140/1141)
Chloroherpeton thalassium ATCC 35110 (Ctha_0400/0401)
Sphaerobacter thermophilus DSM 20745 (Sthe_1568/1567)
Roseiflexus sp. RS-1 (RoseRS_0200/0199)
Roseiflexus castenholzii DSM 13941 (Rcas_4339/4340)
Herpetosiphon aurantiacus DSM 785 (Haur_0931/0932)
Chloroflexus aggregans DSM 9485 (Cagg_2679/2680)
Chloroflexus sp. Y-400-fl (Chy400_2221/2222)
Chloroflexus aurantiacus J-10-fl (Caur_2060/2061)
Caldisericum exile AZM16c01 (CSE_12570/12560)
Dictyoglomus thermophilum H-6-12 (DICTH_1242/1243)
Dictyoglomus turgidum DSM 6724 (Dtur_1356/1357)
Deinococcus peraridilitoris DSM 19664 (Deipe_1147/Deipe_1146)
Deinococcus maricopensis DSM 21211 (Deima_2991/2990)
Deinococcus radiodurans R1 (DR_0853/0854)
Deinococcus gobiensis I-0 (DGo_CA2440/2441)
Deinococcus deserti VCD115 (Deide_17750/17760)
Deinococcus proteolyticus MRP (Deipr_0325/0324)
Deinococcus geothermalis DSM 11300 (Dgeo_1815/Dgeo_1816)
Oceanithermus profundus DSM 14977 (Ocepr_1644/1645)
Marinithermus hydrothermalis DSM 14884 (Marky_0784/0783)
Meiothermus silvanus DSM 9946 (Mesil_2006/2007)
Meiothermus ruber DSM 1279 (Mrub_1713/1714)
Meiothermus ruber DSM 1279 (K649_13995/13990)
Thermus oshimai JL-2 (Theos_1496/1495)
Thermus sp. CCB_US3_UF1 (TCCBUS3UF1_13980/13970)
Thermus scotoductus SA-01 (TSC_c09700/9710)
Thermus thermophilus HB8 (TTHA1132/1131)
Thermus thermophilus SG0.5JP17-16 (Ththe16_1141/1140)
Thermus thermophilus JL-18 (TtJL18_0928/0929)
Thermus thermophilus HB27 (TT_C0767/0766)
1_Desulfomonile tiedjei DSM 6799 (Desti_0304/0303)
2_Desulfomonile tiedjei DSM 6799 (Desti_1397/1398)
Thermodesulfatator indicus DSM 15286 (Thein_0408/0407)
1_Syntrophobacter fumaroxidans MPOB (Sfum_0318/0317)
2_Syntrophobacter fumaroxidans MPOB (Sfum_1654/1653)
Desulfatibacillum alkenivorans AK-01 (Dalk_2485/2486)
Desulfobacula toluolica Tol2 (TOL2_C24040/24030)
Desulfobacterium autotrophicum HRM2 (HRM2_35580/35590)
Desulfobulbus propionicus DSM 2032 (Despr_1663/1662)
Desulfocapsa sulfexigens DSM 10523 (UWK_02584/02583)
Desulfotalea psychrophila LSv54 (DP2776/2777)
Gemmatimonas aurantiaca T-27 (GAU_1116/1115)
Bacteriovorax marinus SJ (BMS_0054/0053)
Candidatus Chloracidobacterium thermophilum B (Cabther_A0781/0780)
Desulfurobacterium thermolithotrophum DSM 11699 (Dester_1068/1069)
Thermovibrio ammonificans HB-1 (Theam_1390/1391)
Deferribacter desulfuricans SSM1 (DEFDS_0024/0025)
Flexistipes sinusarabici DSM 4947 (Flexsi_0290/0291)
Calditerrivibrio nitroreducens DSM 19672 (Calni_1376/1375)
Pelobacter carbinolicus DSM 2380 (Pcar_0379/0378)
Geobacter metallireducens GS-15 (Gmet_3417/3418)
Geobacter lovleyi SZ (Glov_3124/3123)
Pelobacter propionicus DSM 2379 (Ppro_2940/2941)
Geobacter sulfurreducens PCA (GSU0099/0098)
Geobacter sulfurreducens KN400 (KN400_0075/0074)
Geobacter daltonii FRC-32 (Geob_0373/0374)
Geobacter uraniireducens Rf4 (Gura_4332/4331)
Geobacter sp. M18 (GM18_4429/4430)
Geobacter bemidjiensis Bem (Gbem_3962/3963)
Geobacter sp. M21 (GM21_4048/4049)
Haliangium ochraceum DSM 14365 (Hoch_6866/6867)
Plesiocystis pacifica SIR-1 (PPSIR1_33044/33043)
Sorangium cellulosum So ce56 (sce7249/2748)
Anaeromyxobacter sp. Fw109-5 (Anae109_1201/3759)
Anaeromyxobacter dehalogenans 2CP-C (Adeh_3633/3634)
Anaeromyxobacter sp. K (AnaeK_3691/3692)
Anaeromyxobacter dehalogenans 2CP-1 (A2cp1_3774/3775)
Myxococcus stipitatus DSM 14675 (MYSTI_02214/02215)
Corallococcus coralloides DSM 2259 (COCOR_01949/01950)
Cystobacter fuscus DSM 2262 (D187_06791/06792)
Stigmatella aurantiaca DW4/3-1 (STAUR_2690/2691)
Chondromyces apiculatus DSM 436 (A176_2765/2766)
Myxococcus fulvus HW-1 (LILAB_17450/17455)
Myxococcus xanthus DK 1622 (MXAN_1925/1926)
Euryarchaeota
Aquificae
Chlorobi
Chloroflexi
Caldiserica
Dictyoglomi
Deinococcus-Thermus
Deltaproteobacteria
Thermodesulfobacteria
Deltaproteobacteria
Gemmatimonadetes
Deltaproteobacteria
Acidobacteria
Aquificae
Deferribacteres
Deltaproteobacteria
0.4
