# Supplementary material for: Evolution and Design Governing Signal Precision and Amplification in a Bacterial Chemosensory Pathway
Source: PLoS Genet. 2015 Aug 20;11(8):e1005460. doi: 10.1371/journal.pgen.1005460 (PMC4546325; doi:10.1371/journal.pgen.1005460)
Supplement: S3 Fig — Shown are unrooted Bayesian phylogenetic trees of the signaling proteins: (A) FrzCD (MXAN_4141, 191 sequences, 288 positions), (B) FrzA (MXAN_4143, 98 sequences and 84 positions), (C) FrzE (MXAN_4140, 144 sequences, 482 positions), (D) FrzF (MXAN_4138, 104 sequences, 276 positions), (E) FrzG (MXAN_4139, 132 sequences, 283 positions), (F) FrzB (MXAN_4142, 11 sequences, 113 positions) and (G) FrzZ (MXAN_4144, 64 sequences, 102 positions). Number at nodes indicates posterior probabilities (PP) and bootstrap support (BS) computed by Mrbayes and PhyMl, respectively. Only posterior probabilities and bootstrap values greater, respectively, than 0.5 and 50% are shown. The scale bars represent the number of substitutions per site. In each phylogenetic tree Frz proteins from M. xanthus are illustrated with color-coded gene symbols The domain composition is illustrated in the right. (PDF) [file pgen.1005460.s003.pdf]

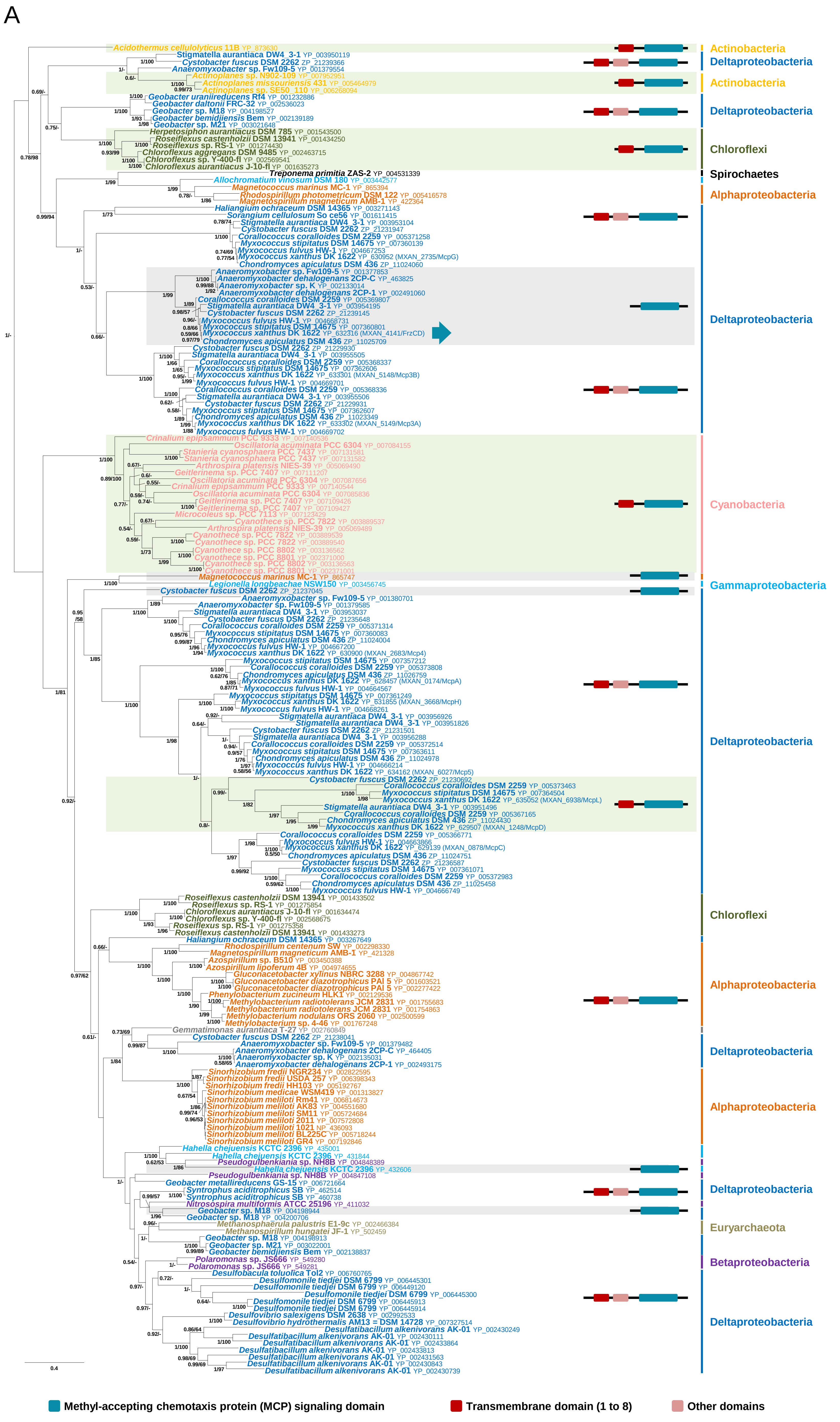

A
Acidothermus cellulolyticus 11B YP_873630
Stigmatella aurantiaca DW4_3-1 YP_003950119
Cystobacter fuscus DSM 2262 ZP_21239366
Anaeromyxobacter sp. Fw109-5 YP_001379554
Actinoplanes sp. N902-109 YP_007952951
Actinoplanes missouriensis 431 YP_005464979
Actinoplanes sp. SE50_110 YP_006268094
Geobacter uraniireducens Rf4 YP_001232886
Geobacter daltonii FRC-32 YP_002536023
Geobacter sp. M18 YP_004198527
Geobacter bemidjiensis Bem YP_002139189
Geobacter sp. M21 YP_003021648
Herpetosiphon aurantiacus DSM 785 YP_001543500
Roseiflexus castenholzii DSM 13941 YP_001434250
Roseiflexus sp. RS-1 YP_001274430
Chloroflexus aggregans DSM 9485 YP_002463715
Chloroflexus sp. Y-400-fl YP_002569541
Chloroflexus aurantiacus J-10-fl YP_001635273
Treponema primitia ZAS-2 YP_004531339
Allochromatium vinosum DSM 180 YP_003442577
Magnetococcus marinus MC-1 YP_865394
Rhodospirillum photometricum DSM 122 YP_005416578
Magnetospirillum magneticum AMB-1 YP_422364
Haliangium ochraceum DSM 14365 YP_003271143
Sorangium cellulosum So ce56 YP_001611415
Stigmatella aurantiaca DW4_3-1 YP_003953104
Cystobacter fuscus DSM 2262 ZP_21231947
Corallococcus coralloides DSM 2259 YP_005371258
Myxococcus stipitatus DSM 14675 YP_007360139
Myxococcus fulvus HW-1 YP_004667253
Myxococcus xanthus DK 1622 YP_630952 (MXAN_2735/McpG)
Chondromyces apiculatus DSM 436 ZP_11024060
Anaeromyxobacter sp. Fw109-5 YP_001377853
Anaeromyxobacter dehalogenans 2CP-C YP_463825
Anaeromyxobacter sp. K YP_002133014
Anaeromyxobacter dehalogenans 2CP-1 YP_002491060
Corallococcus coralloides DSM 2259 YP_005369807
Stigmatella aurantiaca DW4_3-1 YP_003954195
Cystobacter fuscus DSM 2262 ZP_21239145
Myxococcus fulvus HW-1 YP_004668731
Myxococcus stipitatus DSM 14675 YP_007360801
Myxococcus xanthus DK 1622 YP_632316 (MXAN_4141/FrzCD)
Chondromyces apiculatus DSM 436 ZP_11025709
Cystobacter fuscus DSM 2262 ZP_21229930
Stigmatella aurantiaca DW4_3-1 YP_003955505
Corallococcus coralloides DSM 2259 YP_005368337
Myxococcus stipitatus DSM 14675 YP_007362606
Myxococcus xanthus DK 1622 YP_633301 (MXAN_5148/Mcp3B)
Myxococcus fulvus HW-1 YP_004669701
Corallococcus coralloides DSM 2259 YP_005368336
Stigmatella aurantiaca DW4_3-1 YP_003955506
Cystobacter fuscus DSM 2262 ZP_21229931
Myxococcus stipitatus DSM 14675 YP_007362607
Chondromyces apiculatus DSM 436 ZP_11023349
Myxococcus xanthus DK 1622 YP_633302 (MXAN_5149/Mcp3A)
Myxococcus fulvus HW-1 YP_004669702
Crinalium epipsammum PCC 9333 YP_007140536
Oscillatoria acuminata PCC 6304 YP_007084155
Stanieria cyanosphaera PCC 7437 YP_007131581
Stanieria cyanosphaera PCC 7437 YP_007131582
Arthrospira platensis NIES-39 YP_005069490
Geitlerinema sp. PCC 7407 YP_007111207
Oscillatoria acuminata PCC 6304 YP_007087656
Crinalium epipsammum PCC 9333 YP_007140544
Oscillatoria acuminata PCC 6304 YP_007085836
Geitlerinema sp. PCC 7407 YP_007109426
Geitlerinema sp. PCC 7407 YP_007109427
Microcoleus sp. PCC 7113 YP_007123429
Cyanothece sp. PCC 7822 YP_003889537
Arthrospira platensis NIES-39 YP_005069489
Cyanothece sp. PCC 7822 YP_003889539
Cyanothece sp. PCC 7822 YP_003889540
Cyanothece sp. PCC 8802 YP_003136562
Cyanothece sp. PCC 8801 YP_002371000
Cyanothece sp. PCC 8802 YP_003136563
Cyanothece sp. PCC 8801 YP_002371001
Magnetococcus marinus MC-1 YP_865747
Legionella longbeachae NSW150 YP_003456745
Cystobacter fuscus DSM 2262 ZP_21237045
Anaeromyxobacter sp. Fw109-5 YP_001380701
Anaeromyxobacter sp. Fw109-5 YP_001379585
Stigmatella aurantiaca DW4_3-1 YP_003953037
Cystobacter fuscus DSM 2262 ZP_21235648
Corallococcus coralloides DSM 2259 YP_005371314
Myxococcus stipitatus DSM 14675 YP_007360083
Chondromyces apiculatus DSM 436 ZP_11024004
Myxococcus fulvus HW-1 YP_004667200
Myxococcus xanthus DK 1622 YP_630900 (MXAN_2683/Mcp4)
Myxococcus stipitatus DSM 14675 YP_007357212
Corallococcus coralloides DSM 2259 YP_005373808
Chondromyces apiculatus DSM 436 ZP_11026759
Myxococcus xanthus DK 1622 YP_628457 (MXAN_0174/McpA)
Myxococcus fulvus HW-1 YP_004664567
Myxococcus stipitatus DSM 14675 YP_007361249
Myxococcus xanthus DK 1622 YP_631855 (MXAN_3668/McpH)
Myxococcus fulvus HW-1 YP_004668261
Stigmatella aurantiaca DW4_3-1 YP_003956926
Stigmatella aurantiaca DW4_3-1 YP_003951826
Cystobacter fuscus DSM 2262 ZP_21231501
Stigmatella aurantiaca DW4_3-1 YP_003956288
Corallococcus coralloides DSM 2259 YP_005372514
Myxococcus stipitatus DSM 14675 YP_007363611
Chondromyces apiculatus DSM 436 ZP_11024978
Myxococcus fulvus HW-1 YP_004666214
Myxococcus xanthus DK 1622 YP_634162 (MXAN_6027/Mcp5)
Cystobacter fuscus DSM 2262 ZP_21230692
Corallococcus coralloides DSM 2259 YP_005373463
Myxococcus stipitatus DSM 14675 YP_007364504
Myxococcus xanthus DK 1622 YP_635052 (MXAN_6938/McpL)
Stigmatella aurantiaca DW4_3-1 YP_003951496
Corallococcus coralloides DSM 2259 YP_005367165
Chondromyces apiculatus DSM 436 ZP_11024430
Myxococcus xanthus DK 1622 YP_629507 (MXAN_1248/McpD)
Corallococcus coralloides DSM 2259 YP_005366771
Myxococcus fulvus HW-1 YP_004663866
Myxococcus xanthus DK 1622 YP_629139 (MXAN_0878/McpC)
Chondromyces apiculatus DSM 436 ZP_11024751
Cystobacter fuscus DSM 2262 ZP_21236587
Myxococcus stipitatus DSM 14675 YP_007361071
Corallococcus coralloides DSM 2259 YP_005372983
Chondromyces apiculatus DSM 436 ZP_11025458
Myxococcus fulvus HW-1 YP_004666749
Roseiflexus castenholzii DSM 13941 YP_001433502
Roseiflexus sp. RS-1 YP_001275854
Chloroflexus aurantiacus J-10-fl YP_001634474
Chloroflexus sp. Y-400-fl YP_002568675
Roseiflexus sp. RS-1 YP_001275358
Roseiflexus castenholzii DSM 13941 YP_001433273
Haliangium ochraceum DSM 14365 YP_003267649
Rhodospirillum centenum SW YP_002298330
Magnetospirillum magneticum AMB-1 YP_421328
Azospirillum sp. B510 YP_003450388
Azospirillum lipoferum 4B YP_004974655
Gluconacetobacter xylinus NBRC 3288 YP_004867742
Gluconacetobacter diazotrophicus PAl 5 YP_001603521
Gluconacetobacter diazotrophicus PAl 5 YP_002277422
Phenylobacterium zucineum HLK1 YP_002129536
Methylobacterium radiotolerans JCM 2831 YP_001755683
Methylobacterium radiotolerans JCM 2831 YP_001754863
Methylobacterium nodulans ORS 2060 YP_002500599
Methylobacterium sp. 4-46 YP_001767248
Gemmatimonas aurantiaca T-27 YP_002760849
Cystobacter fuscus DSM 2262 ZP_21238041
Anaeromyxobacter sp. Fw109-5 YP_001379482
Anaeromyxobacter dehalogenans 2CP-C YP_464405
Anaeromyxobacter sp. K YP_002135031
Anaeromyxobacter dehalogenans 2CP-1 YP_002493175
Sinorhizobium fredii NGR234 YP_002822595
Sinorhizobium fredii USDA 257 YP_006398343
Sinorhizobium fredii HH103 YP_005192767
Sinorhizobium medicae WSM419 YP_001313827
Sinorhizobium meliloti Rm41 YP_006814673
Sinorhizobium meliloti AK83 YP_004551680
Sinorhizobium meliloti SM11 YP_005724684
Sinorhizobium meliloti 2011 YP_007572808
Sinorhizobium meliloti 1021 NP_436093
Sinorhizobium meliloti BL225C YP_005718244
Sinorhizobium meliloti GR4 YP_007192846
Hahella chejuensis KCTC 2396 YP_435001
Hahella chejuensis KCTC 2396 YP_431844
Pseudogulbenkiania sp. NH8B YP_004848389
Hahella chejuensis KCTC 2396 YP_432606
Pseudogulbenkiania sp. NH8B YP_004847108
Geobacter metallireducens GS-15 YP_006721664
Syntrophus aciditrophicus SB YP_462514
Syntrophus aciditrophicus SB YP_460738
Nitrosospira multiformis ATCC 25196 YP_411032
Geobacter sp. M18 YP_004198944
Geobacter sp. M18 YP_004200706
Methanosphaerula palustris E1-9c YP_002466384
Methanospirillum hungatei JF-1 YP_502459
Geobacter sp. M18 YP_004198913
Geobacter sp. M21 YP_003022001
Geobacter bemidjiensis Bem YP_002138837
Polaromonas sp. JS666 YP_549280
Polaromonas sp. JS666 YP_549281
Desulfobacula toluolica Tol2 YP_006760765
Desulfomonile tiedjei DSM 6799 YP_006445301
Desulfomonile tiedjei DSM 6799 YP_006449120
Desulfomonile tiedjei DSM 6799 YP_006445300
Desulfomonile tiedjei DSM 6799 YP_006445913
Desulfomonile tiedjei DSM 6799 YP_006445914
Desulfovibrio salexigens DSM 2638 YP_002992533
Desulfovibrio hydrothermalis AM13 = DSM 14728 YP_007327514
Desulfatibacillum alkenivorans AK-01 YP_002430249
Desulfatibacillum alkenivorans AK-01 YP_002430111
Desulfatibacillum alkenivorans AK-01 YP_002433864
Desulfatibacillum alkenivorans AK-01 YP_002433813
Desulfatibacillum alkenivorans AK-01 YP_002431563
Desulfatibacillum alkenivorans AK-01 YP_002430843
Desulfatibacillum alkenivorans AK-01 YP_002430739
0.4
Actinobacteria
Deltaproteobacteria
Actinobacteria
Deltaproteobacteria
Chloroflexi
Spirochaetes
Alphaproteobacteria
Deltaproteobacteria
Cyanobacteria
Gammaproteobacteria
Deltaproteobacteria
Chloroflexi
Alphaproteobacteria
Deltaproteobacteria
Alphaproteobacteria
Deltaproteobacteria
Euryarchaeota
Betaproteobacteria
Deltaproteobacteria
Methyl-accepting chemotaxis protein (MCP) signaling domain
Transmembrane domain (1 to 8)
Other domains

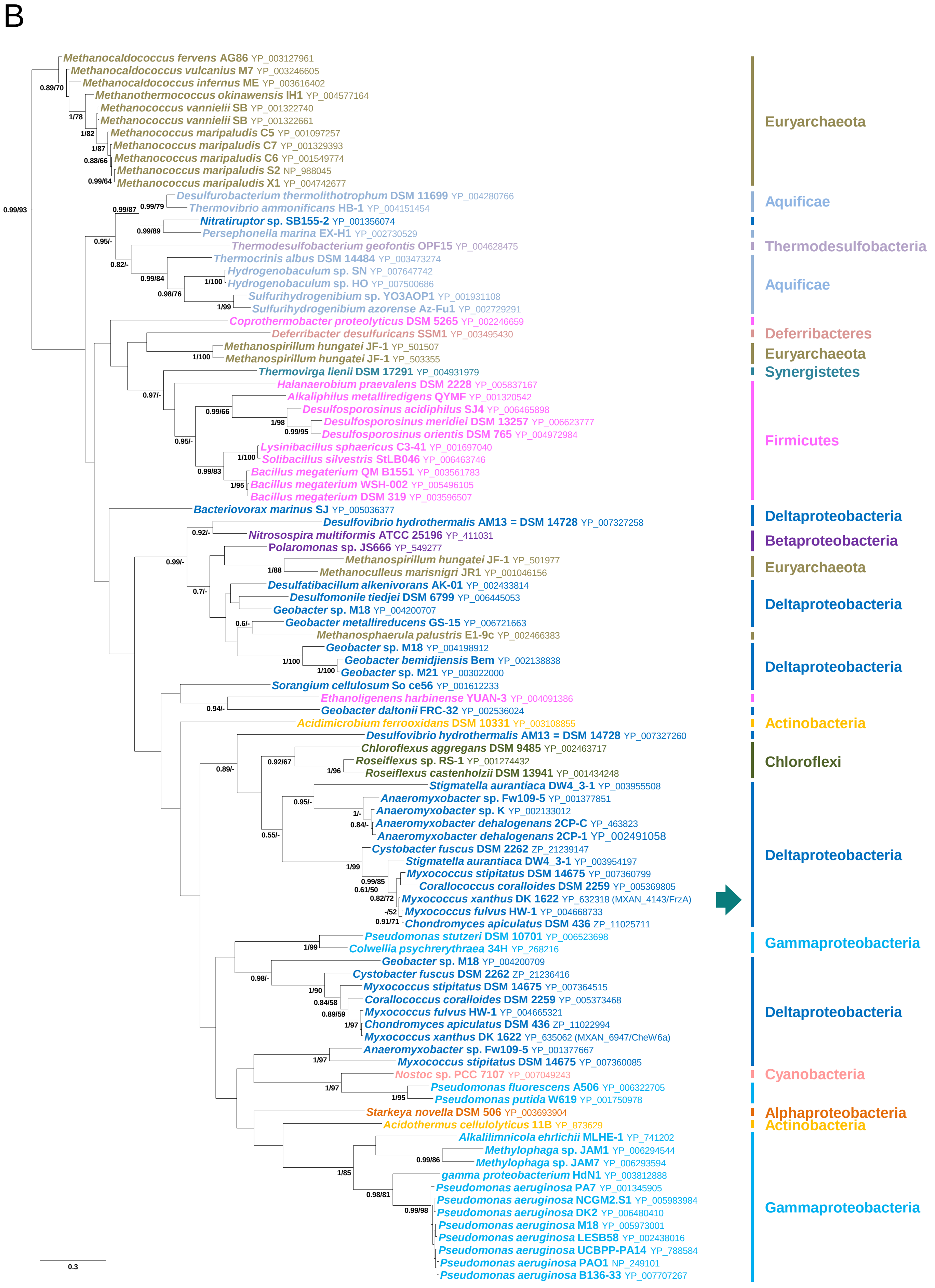
B
Methanocaldococcus fervens AG86 YP_003127961
Methanocaldococcus vulcanius M7 YP_003246605
Methanocaldococcus infernus ME YP_003616402
Methanothermococcus okinawensis IH1 YP_004577164
Methanococcus vannielii SB YP_001322740
Methanococcus vannielii SB YP_001322661
Methanococcus maripaludis C5 YP_001097257
Methanococcus maripaludis C7 YP_001329393
Methanococcus maripaludis C6 YP_001549774
Methanococcus maripaludis S2 NP_988045
Methanococcus maripaludis X1 YP_004742677
Desulfurobacterium thermolithotrophum DSM 11699 YP_004280766
Thermovibrio ammonificans HB-1 YP_004151454
Nitratiruptor sp. SB155-2 YP_001356074
Persephonella marina EX-H1 YP_002730529
Thermodesulfobacterium geofontis OPF15 YP_004628475
Thermocrinis albus DSM 14484 YP_003473274
Hydrogenobaculum sp. SN YP_007647742
Hydrogenobaculum sp. HO YP_007500686
Sulfurihydrogenibium sp. YO3AOP1 YP_001931108
Sulfurihydrogenibium azorense Az-Fu1 YP_002729291
Coprothermobacter proteolyticus DSM 5265 YP_002246659
Deferribacter desulfuricans SSM1 YP_003495430
Methanospirillum hungatei JF-1 YP_501507
Methanospirillum hungatei JF-1 YP_503355
Thermovirga lienii DSM 17291 YP_004931979
Halanaerobium praevalens DSM 2228 YP_005837167
Alkaliphilus metalliredigens QYMF YP_001320542
Desulfosporosinus acidiphilus SJ4 YP_006465898
Desulfosporosinus meridiei DSM 13257 YP_006623777
Desulfosporosinus orientis DSM 765 YP_004972984
Lysinibacillus sphaericus C3-41 YP_001697040
Solibacillus silvestris StLB046 YP_006463746
Bacillus megaterium QM B1551 YP_003561783
Bacillus megaterium WSH-002 YP_005496105
Bacillus megaterium DSM 319 YP_003596507
Bacteriovorax marinus SJ YP_005036377
Desulfovibrio hydrothermalis AM13 = DSM 14728 YP_007327258
Nitrosospira multiformis ATCC 25196 YP_411031
Polaromonas sp. JS666 YP_549277
Methanospirillum hungatei JF-1 YP_501977
Methanoculleus marisnigri JR1 YP_001046156
Desulfatibacillum alkenivorans AK-01 YP_002433814
Desulfomonile tiedjei DSM 6799 YP_006445053
Geobacter sp. M18 YP_004200707
Geobacter metallireducens GS-15 YP_006721663
Methanosphaerula palustris E1-9c YP_002466383
Geobacter sp. M18 YP_004198912
Geobacter bemidjiensis Bem YP_002138838
Geobacter sp. M21 YP_003022000
Sorangium cellulosum So ce56 YP_001612233
Ethanoligenens harbinense YUAN-3 YP_004091386
Geobacter daltonii FRC-32 YP_002536024
Acidimicrobium ferrooxidans DSM 10331 YP_003108855
Desulfovibrio hydrothermalis AM13 = DSM 14728 YP_007327260
Chloroflexus aggregans DSM 9485 YP_002463717
Roseiflexus sp. RS-1 YP_001274432
Roseiflexus castenholzii DSM 13941 YP_001434248
Stigmatella aurantiaca DW4_3-1 YP_003955508
Anaeromyxobacter sp. Fw109-5 YP_001377851
Anaeromyxobacter sp. K YP_002133012
Anaeromyxobacter dehalogenans 2CP-C YP_463823
Anaeromyxobacter dehalogenans 2CP-1 YP_002491058
Cystobacter fuscus DSM 2262 ZP_21239147
Stigmatella aurantiaca DW4_3-1 YP_003954197
Myxococcus stipitatus DSM 14675 YP_007360799
Corallococcus coralloides DSM 2259 YP_005369805
Myxococcus xanthus DK 1622 YP_632318 (MXAN_4143/FrzA)
Myxococcus fulvus HW-1 YP_004668733
Chondromyces apiculatus DSM 436 ZP_11025711
Pseudomonas stutzeri DSM 10701 YP_006523698
Colwellia psychrerythraea 34H YP_268216
Geobacter sp. M18 YP_004200709
Cystobacter fuscus DSM 2262 ZP_21236416
Myxococcus stipitatus DSM 14675 YP_007364515
Corallococcus coralloides DSM 2259 YP_005373468
Myxococcus fulvus HW-1 YP_004665321
Chondromyces apiculatus DSM 436 ZP_11022994
Myxococcus xanthus DK 1622 YP_635062 (MXAN_6947/CheW6a)
Anaeromyxobacter sp. Fw109-5 YP_001377667
Myxococcus stipitatus DSM 14675 YP_007360085
Nostoc sp. PCC 7107 YP_007049243
Pseudomonas fluorescens A506 YP_006322705
Pseudomonas putida W619 YP_001750978
Starkeya novella DSM 506 YP_003693904
Acidothermus cellulolyticus 11B YP_873629
Alkalilimnicola ehrlichii MLHE-1 YP_741202
Methylophaga sp. JAM1 YP_006294544
Methylophaga sp. JAM7 YP_006293594
gamma proteobacterium HdN1 YP_003812888
Pseudomonas aeruginosa PA7 YP_001345905
Pseudomonas aeruginosa NCGM2.S1 YP_005983984
Pseudomonas aeruginosa DK2 YP_006480410
Pseudomonas aeruginosa M18 YP_005973001
Pseudomonas aeruginosa LESB58 YP_002438016
Pseudomonas aeruginosa UCBPP-PA14 YP_788584
Pseudomonas aeruginosa PAO1 NP_249101
Pseudomonas aeruginosa B136-33 YP_007707267
Euryarchaeota
Aquificae
Thermodesulfobacteria
Aquificae
Deferribacteres
Euryarchaeota
Synergistetes
Firmicutes
Deltaproteobacteria
Betaproteobacteria
Euryarchaeota
Deltaproteobacteria
Deltaproteobacteria
Actinobacteria
Chloroflexi
Deltaproteobacteria
Gammaproteobacteria
Deltaproteobacteria
Cyanobacteria
Alphaproteobacteria
Actinobacteria
Gammaproteobacteria
0.3

C

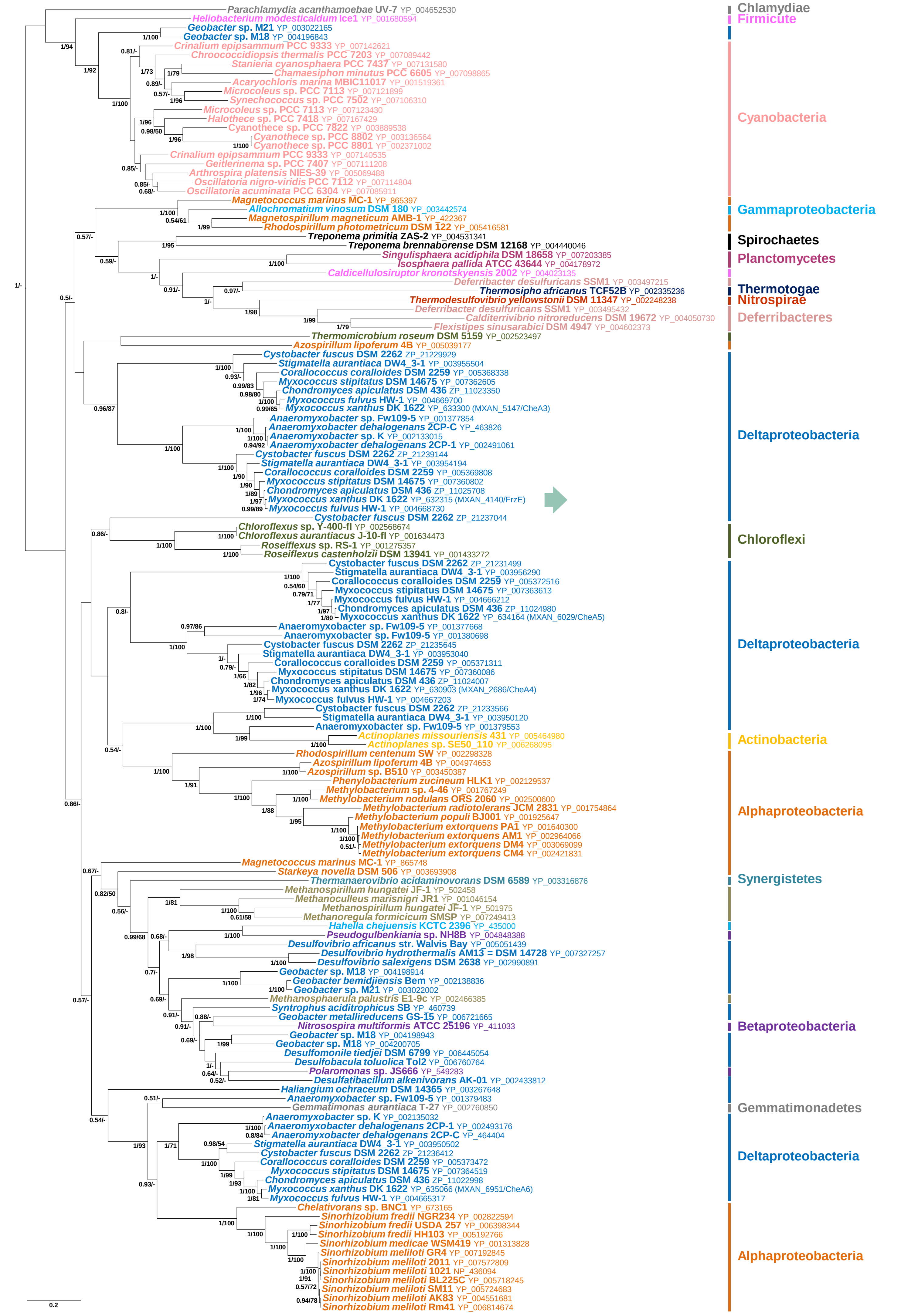

Parachlamydia acanthamoebae UV-7 YP_004652530
Heliobacterium modesticaldum Ice1 YP_001680594
Geobacter sp. M21 YP_003022165
Geobacter sp. M18 YP_004196843
Crinalium epipsammum PCC 9333 YP_007142621
Chroococcidiopsis thermalis PCC 7203 YP_007089442
Stanieria cyanosphaera PCC 7437 YP_007131580
Chamaesiphon minutus PCC 6605 YP_007098865
Acaryochloris marina MBIC11017 YP_001519361
Microcoleus sp. PCC 7113 YP_007121899
Synechococcus sp. PCC 7502 YP_007106310
Microcoleus sp. PCC 7113 YP_007123430
Halothece sp. PCC 7418 YP_007167429
Cyanothece sp. PCC 7822 YP_003889538
Cyanothece sp. PCC 8802 YP_003136564
Cyanothece sp. PCC 8801 YP_002371002
Crinalium epipsammum PCC 9333 YP_007140535
Geitlerinema sp. PCC 7407 YP_007111208
Arthrospira platensis NIES-39 YP_005069488
Oscillatoria nigro-viridis PCC 7112 YP_007114804
Oscillatoria acuminata PCC 6304 YP_007085911
Magnetococcus marinus MC-1 YP_865397
Allochromatium vinosum DSM 180 YP_003442574
Magnetospirillum magneticum AMB-1 YP_422367
Rhodospirillum photometricum DSM 122 YP_005416581
Treponema primitia ZAS-2 YP_004531341
Treponema brennaborense DSM 12168 YP_004440046
Singulisphaera acidiphila DSM 18658 YP_007203385
Isosphaera pallida ATCC 43644 YP_004178972
Caldicellulosiruptor kronotskyensis 2002 YP_004023135
Deferribacter desulfuricans SSM1 YP_003497215
Thermosipho africanus TCF52B YP_002335236
Thermodesulfovibrio yellowstonii DSM 11347 YP_002248238
Deferribacter desulfuricans SSM1 YP_003495432
Calditerrivibrio nitroreducens DSM 19672 YP_004050730
Flexistipes sinusarabici DSM 4947 YP_004602373
Thermomicrobium roseum DSM 5159 YP_002523497
Azospirillum lipoferum 4B YP_005039177
Cystobacter fuscus DSM 2262 ZP_21229929
Stigmatella aurantiaca DW4_3-1 YP_003955504
Corallococcus coralloides DSM 2259 YP_005368338
Myxococcus stipitatus DSM 14675 YP_007362605
Chondromyces apiculatus DSM 436 ZP_11023350
Myxococcus fulvus HW-1 YP_004669700
Myxococcus xanthus DK 1622 YP_633300 (MXAN_5147/CheA3)
Anaeromyxobacter sp. Fw109-5 YP_001377854
Anaeromyxobacter dehalogenans 2CP-C YP_463826
Anaeromyxobacter sp. K YP_002133015
Anaeromyxobacter dehalogenans 2CP-1 YP_002491061
Cystobacter fuscus DSM 2262 ZP_21239144
Stigmatella aurantiaca DW4_3-1 YP_003954194
Corallococcus coralloides DSM 2259 YP_005369808
Myxococcus stipitatus DSM 14675 YP_007360802
Chondromyces apiculatus DSM 436 ZP_11025708
Myxococcus xanthus DK 1622 YP_632315 (MXAN_4140/FrzE)
Myxococcus fulvus HW-1 YP_004668730
Cystobacter fuscus DSM 2262 ZP_21237044
Chloroflexus sp. Y-400-fl YP_002568674
Chloroflexus aurantiacus J-10-fl YP_001634473
Roseiflexus sp. RS-1 YP_001275357
Roseiflexus castenholzii DSM 13941 YP_001433272
Cystobacter fuscus DSM 2262 ZP_21231499
Stigmatella aurantiaca DW4_3-1 YP_003956290
Corallococcus coralloides DSM 2259 YP_005372516
Myxococcus stipitatus DSM 14675 YP_007363613
Myxococcus fulvus HW-1 YP_004666212
Chondromyces apiculatus DSM 436 ZP_11024980
Myxococcus xanthus DK 1622 YP_634164 (MXAN_6029/CheA5)
Anaeromyxobacter sp. Fw109-5 YP_001377668
Anaeromyxobacter sp. Fw109-5 YP_001380698
Cystobacter fuscus DSM 2262 ZP_21235645
Stigmatella aurantiaca DW4_3-1 YP_003953040
Corallococcus coralloides DSM 2259 YP_005371311
Myxococcus stipitatus DSM 14675 YP_007360086
Chondromyces apiculatus DSM 436 ZP_11024007
Myxococcus xanthus DK 1622 YP_630903 (MXAN_2686/CheA4)
Myxococcus fulvus HW-1 YP_004667203
Cystobacter fuscus DSM 2262 ZP_21233566
Stigmatella aurantiaca DW4_3-1 YP_003950120
Anaeromyxobacter sp. Fw109-5 YP_001379553
Actinoplanes missouriensis 431 YP_005464980
Actinoplanes sp. SE50_110 YP_006268095
Rhodospirillum centenum SW YP_002298328
Azospirillum lipoferum 4B YP_004974653
Azospirillum sp. B510 YP_003450387
Phenylobacterium zucineum HLK1 YP_002129537
Methylobacterium sp. 4-46 YP_001767249
Methylobacterium nodulans ORS 2060 YP_002500600
Methylobacterium radiotolerans JCM 2831 YP_001754864
Methylobacterium populi BJ001 YP_001925647
Methylobacterium extorquens PA1 YP_001640300
Methylobacterium extorquens AM1 YP_002964066
Methylobacterium extorquens DM4 YP_003069099
Methylobacterium extorquens CM4 YP_002421831
Magnetococcus marinus MC-1 YP_865748
Starkeya novella DSM 506 YP_003693908
Thermanaerovibrio acidaminovorans DSM 6589 YP_003316876
Methanospirillum hungatei JF-1 YP_502458
Methanoculleus marisnigri JR1 YP_001046154
Methanospirillum hungatei JF-1 YP_501975
Methanoregula formicicum SMSP YP_007249413
Hahella chejuensis KCTC 2396 YP_435000
Pseudogulbenkiania sp. NH8B YP_004848388
Desulfovibrio africanus str. Walvis Bay YP_005051439
Desulfovibrio hydrothermalis AM13 = DSM 14728 YP_007327257
Desulfovibrio salexigens DSM 2638 YP_002990891
Geobacter sp. M18 YP_004198914
Geobacter bemidjiensis Bem YP_002138836
Geobacter sp. M21 YP_003022002
Methanosphaerula palustris E1-9c YP_002466385
Syntrophus aciditrophicus SB YP_460739
Geobacter metallireducens GS-15 YP_006721665
Nitrosospira multiformis ATCC 25196 YP_411033
Geobacter sp. M18 YP_004198943
Geobacter sp. M18 YP_004200705
Desulfomonile tiedjei DSM 6799 YP_006445054
Desulfobacula toluolica Tol2 YP_006760764
Polaromonas sp. JS666 YP_549283
Desulfatibacillum alkenivorans AK-01 YP_002433812
Haliangium ochraceum DSM 14365 YP_003267648
Anaeromyxobacter sp. Fw109-5 YP_001379483
Gemmatimonas aurantiaca T-27 YP_002760850
Anaeromyxobacter sp. K YP_002135032
Anaeromyxobacter dehalogenans 2CP-1 YP_002493176
Anaeromyxobacter dehalogenans 2CP-C YP_464404
Stigmatella aurantiaca DW4_3-1 YP_003950502
Cystobacter fuscus DSM 2262 ZP_21236412
Corallococcus coralloides DSM 2259 YP_005373472
Myxococcus stipitatus DSM 14675 YP_007364519
Chondromyces apiculatus DSM 436 ZP_11022998
Myxococcus xanthus DK 1622 YP_635066 (MXAN_6951/CheA6)
Myxococcus fulvus HW-1 YP_004665317
Chelativorans sp. BNC1 YP_673165
Sinorhizobium fredii NGR234 YP_002822594
Sinorhizobium fredii USDA 257 YP_006398344
Sinorhizobium fredii HH103 YP_005192766
Sinorhizobium medicae WSM419 YP_001313828
Sinorhizobium meliloti GR4 YP_007192845
Sinorhizobium meliloti 2011 YP_007572809
Sinorhizobium meliloti 1021 NP_436094
Sinorhizobium meliloti BL225C YP_005718245
Sinorhizobium meliloti SM11 YP_005724683
Sinorhizobium meliloti AK83 YP_004551681
Sinorhizobium meliloti Rm41 YP_006814674
Chlamydiae
Firmicute
Cyanobacteria
Gammaproteobacteria
Spirochaetes
Planctomycetes
Thermotogae
Nitrospirae
Deferribacteres
Deltaproteobacteria
Chloroflexi
Deltaproteobacteria
Actinobacteria
Alphaproteobacteria
Synergistetes
Betaproteobacteria
Gemmatimonadetes
Deltaproteobacteria
Alphaproteobacteria
0.2

D

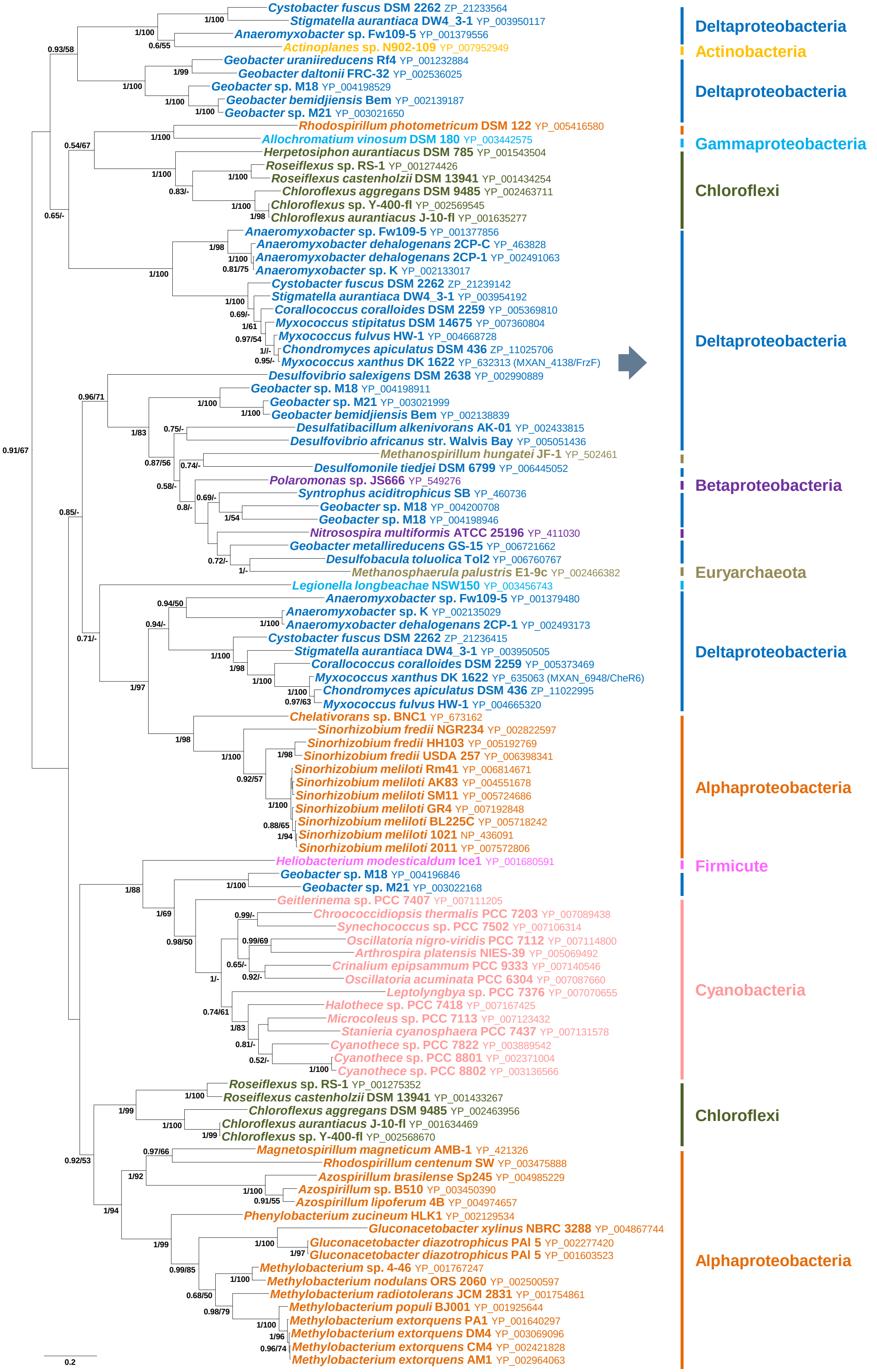
Cystobacter fuscus DSM 2262 ZP_21233564
Stigmatella aurantiaca DW4_3-1 YP_003950117
Anaeromyxobacter sp. Fw109-5 YP_001379556
Actinoplanes sp. N902-109 YP_007952949
Geobacter uraniireducens Rf4 YP_001232884
Geobacter daltonii FRC-32 YP_002536025
Geobacter sp. M18 YP_004198529
Geobacter bemidjiensis Bem YP_002139187
Geobacter sp. M21 YP_003021650
Rhodospirillum photometricum DSM 122 YP_005416580
Allochromatium vinosum DSM 180 YP_003442575
Herpetosiphon aurantiacus DSM 785 YP_001543504
Roseiflexus sp. RS-1 YP_001274426
Roseiflexus castenholzii DSM 13941 YP_001434254
Chloroflexus aggregans DSM 9485 YP_002463711
Chloroflexus sp. Y-400-fl YP_002569545
Chloroflexus aurantiacus J-10-fl YP_001635277
Anaeromyxobacter sp. Fw109-5 YP_001377856
Anaeromyxobacter dehalogenans 2CP-C YP_463828
Anaeromyxobacter dehalogenans 2CP-1 YP_002491063
Anaeromyxobacter sp. K YP_002133017
Cystobacter fuscus DSM 2262 ZP_21239142
Stigmatella aurantiaca DW4_3-1 YP_003954192
Corallococcus coralloides DSM 2259 YP_005369810
Myxococcus stipitatus DSM 14675 YP_007360804
Myxococcus fulvus HW-1 YP_004668728
Chondromyces apiculatus DSM 436 ZP_11025706
Myxococcus xanthus DK 1622 YP_632313 (MXAN_4138/FrzF)
Desulfovibrio salexigens DSM 2638 YP_002990889
Geobacter sp. M18 YP_004198911
Geobacter sp. M21 YP_003021999
Geobacter bemidjiensis Bem YP_002138839
Desulfatibacillum alkenivorans AK-01 YP_002433815
Desulfovibrio africanus str. Walvis Bay YP_005051436
Methanospirillum hungatei JF-1 YP_502461
Desulfomonile tiedjei DSM 6799 YP_006445052
Polaromonas sp. JS666 YP_549276
Syntrophus aciditrophicus SB YP_460736
Geobacter sp. M18 YP_004200708
Geobacter sp. M18 YP_004198946
Nitrosospira multiformis ATCC 25196 YP_411030
Geobacter metallireducens GS-15 YP_006721662
Desulfobacula toluolica Tol2 YP_006760767
Methanosphaerula palustris E1-9c YP_002466382
Legionella longbeachae NSW150 YP_003456743
Anaeromyxobacter sp. Fw109-5 YP_001379480
Anaeromyxobacter sp. K YP_002135029
Anaeromyxobacter dehalogenans 2CP-1 YP_002493173
Cystobacter fuscus DSM 2262 ZP_21236415
Stigmatella aurantiaca DW4_3-1 YP_003950505
Corallococcus coralloides DSM 2259 YP_005373469
Myxococcus xanthus DK 1622 YP_635063 (MXAN_6948/CheR6)
Chondromyces apiculatus DSM 436 ZP_11022995
Myxococcus fulvus HW-1 YP_004665320
Chelativorans sp. BNC1 YP_673162
Sinorhizobium fredii NGR234 YP_002822597
Sinorhizobium fredii HH103 YP_005192769
Sinorhizobium fredii USDA 257 YP_006398341
Sinorhizobium meliloti Rm41 YP_006814671
Sinorhizobium meliloti AK83 YP_004551678
Sinorhizobium meliloti SM11 YP_005724686
Sinorhizobium meliloti GR4 YP_007192848
Sinorhizobium meliloti BL225C YP_005718242
Sinorhizobium meliloti 1021 NP_436091
Sinorhizobium meliloti 2011 YP_007572806
Heliobacterium modesticaldum Ice1 YP_001680591
Geobacter sp. M18 YP_004196846
Geobacter sp. M21 YP_003022168
Geitlerinema sp. PCC 7407 YP_007111205
Chroococcidiopsis thermalis PCC 7203 YP_007089438
Synechococcus sp. PCC 7502 YP_007106314
Oscillatoria nigro-viridis PCC 7112 YP_007114800
Arthrospira platensis NIES-39 YP_005069492
Crinalium epipsammum PCC 9333 YP_007140546
Oscillatoria acuminata PCC 6304 YP_007087660
Leptolyngbya sp. PCC 7376 YP_007070655
Halothece sp. PCC 7418 YP_007167425
Microcoleus sp. PCC 7113 YP_007123432
Stanieria cyanosphaera PCC 7437 YP_007131578
Cyanothece sp. PCC 7822 YP_003889542
Cyanothece sp. PCC 8801 YP_002371004
Cyanothece sp. PCC 8802 YP_003136566
Roseiflexus sp. RS-1 YP_001275352
Roseiflexus castenholzii DSM 13941 YP_001433267
Chloroflexus aggregans DSM 9485 YP_002463956
Chloroflexus aurantiacus J-10-fl YP_001634469
Chloroflexus sp. Y-400-fl YP_002568670
Magnetospirillum magneticum AMB-1 YP_421326
Rhodospirillum centenum SW YP_003475888
Azospirillum brasilense Sp245 YP_004985229
Azospirillum sp. B510 YP_003450390
Azospirillum lipoferum 4B YP_004974657
Phenylobacterium zucineum HLK1 YP_002129534
Gluconacetobacter xylinus NBRC 3288 YP_004867744
Gluconacetobacter diazotrophicus PAl 5 YP_002277420
Gluconacetobacter diazotrophicus PAl 5 YP_001603523
Methylobacterium sp. 4-46 YP_001767247
Methylobacterium nodulans ORS 2060 YP_002500597
Methylobacterium radiotolerans JCM 2831 YP_001754861
Methylobacterium populi BJ001 YP_001925644
Methylobacterium extorquens PA1 YP_001640297
Methylobacterium extorquens DM4 YP_003069096
Methylobacterium extorquens CM4 YP_002421828
Methylobacterium extorquens AM1 YP_002964063
0.2
Deltaproteobacteria
Actinobacteria
Deltaproteobacteria
Gammaproteobacteria
Chloroflexi
Deltaproteobacteria
Betaproteobacteria
Euryarchaeota
Deltaproteobacteria
Alphaproteobacteria
Firmicute
Cyanobacteria
Chloroflexi
Alphaproteobacteria

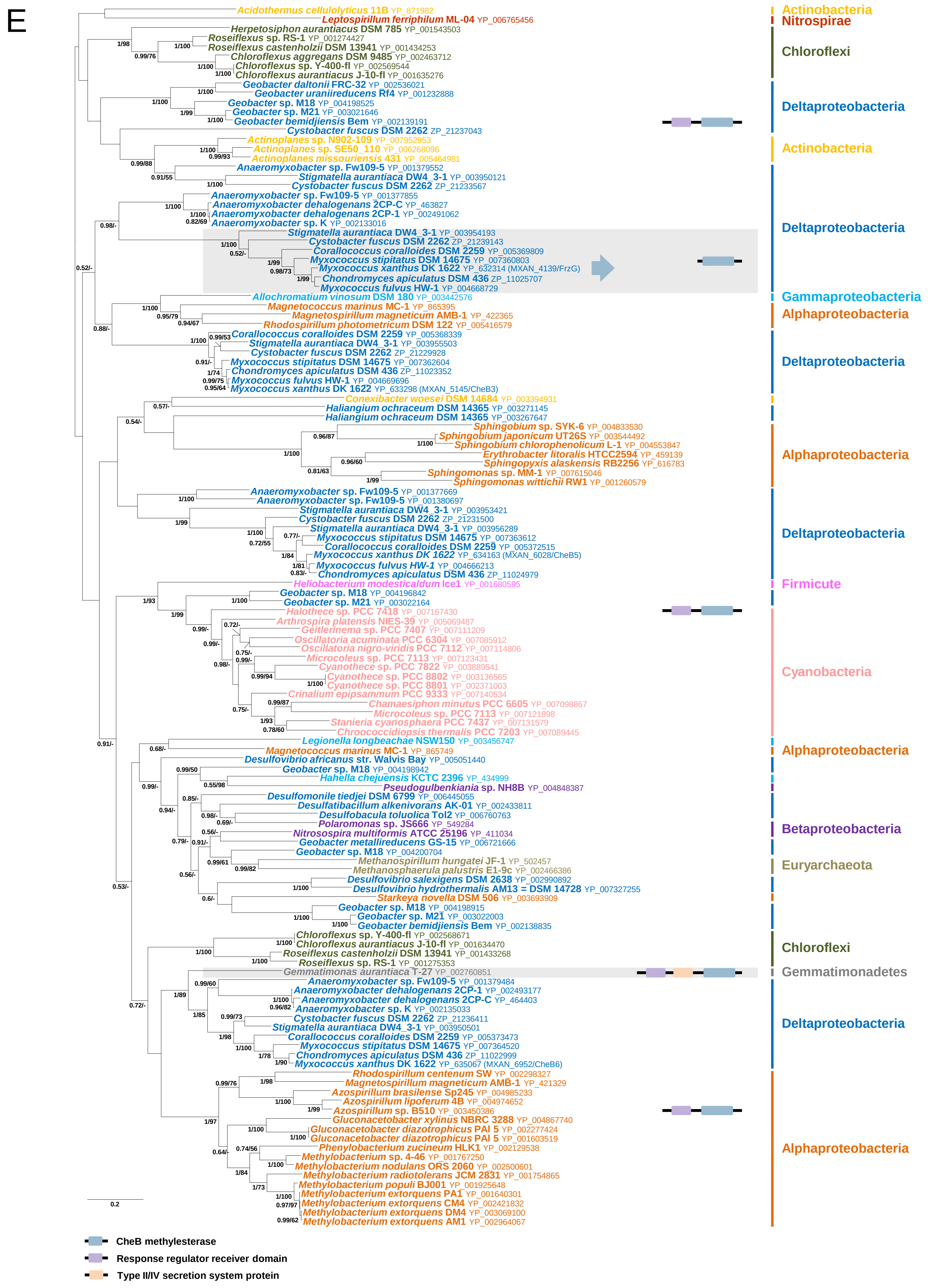
E
Acidothermus cellulolyticus 11B YP_871982
Leptospirillum ferriphilum ML-04 YP_006765456
Herpetosiphon aurantiacus DSM 785 YP_001543503
Roseiflexus sp. RS-1 YP_001274427
Roseiflexus castenholzii DSM 13941 YP_001434253
Chloroflexus aggregans DSM 9485 YP_002463712
Chloroflexus sp. Y-400-fl YP_002569544
Chloroflexus aurantiacus J-10-fl YP_001635276
Geobacter daltonii FRC-32 YP_002536021
Geobacter uraniireducens Rf4 YP_001232888
Geobacter sp. M18 YP_004198525
Geobacter sp. M21 YP_003021646
Geobacter bemidjiensis Bem YP_002139191
Cystobacter fuscus DSM 2262 ZP_21237043
Actinoplanes sp. N902-109 YP_007952953
Actinoplanes sp. SE50_110 YP_006268096
Actinoplanes missouriensis 431 YP_005464981
Anaeromyxobacter sp. Fw109-5 YP_001379552
Stigmatella aurantiaca DW4_3-1 YP_003950121
Cystobacter fuscus DSM 2262 ZP_21233567
Anaeromyxobacter sp. Fw109-5 YP_001377855
Anaeromyxobacter dehalogenans 2CP-C YP_463827
Anaeromyxobacter dehalogenans 2CP-1 YP_002491062
Anaeromyxobacter sp. K YP_002133016
Stigmatella aurantiaca DW4_3-1 YP_003954193
Cystobacter fuscus DSM 2262 ZP_21239143
Corallococcus coralloides DSM 2259 YP_005369809
Myxococcus stipitatus DSM 14675 YP_007360803
Myxococcus xanthus DK 1622 YP_632314 (MXAN_4139/FrzG)
Chondromyces apiculatus DSM 436 ZP_11025707
Myxococcus fulvus HW-1 YP_004668729
Allochromatium vinosum DSM 180 YP_003442576
Magnetococcus marinus MC-1 YP_865395
Magnetospirillum magneticum AMB-1 YP_422365
Rhodospirillum photometricum DSM 122 YP_005416579
Corallococcus coralloides DSM 2259 YP_005368339
Stigmatella aurantiaca DW4_3-1 YP_003955503
Cystobacter fuscus DSM 2262 ZP_21229928
Myxococcus stipitatus DSM 14675 YP_007362604
Chondromyces apiculatus DSM 436 ZP_11023352
Myxococcus fulvus HW-1 YP_004669696
Myxococcus xanthus DK 1622 YP_633298 (MXAN_5145/CheB3)
Conexibacter woesei DSM 14684 YP_003394931
Haliangium ochraceum DSM 14365 YP_003271145
Haliangium ochraceum DSM 14365 YP_003267647
Sphingobium sp. SYK-6 YP_004833530
Sphingobium japonicum UT26S YP_003544492
Sphingobium chlorophenolicum L-1 YP_004553847
Erythrobacter litoralis HTCC2594 YP_459139
Sphingopyxis alaskensis RB2256 YP_616783
Sphingomonas sp. MM-1 YP_007615046
Sphingomonas wittichii RW1 YP_001260579
Anaeromyxobacter sp. Fw109-5 YP_001377669
Anaeromyxobacter sp. Fw109-5 YP_001380697
Stigmatella aurantiaca DW4_3-1 YP_003953421
Cystobacter fuscus DSM 2262 ZP_21231500
Stigmatella aurantiaca DW4_3-1 YP_003956289
Myxococcus stipitatus DSM 14675 YP_007363612
Corallococcus coralloides DSM 2259 YP_005372515
Myxococcus xanthus DK 1622 YP_634163 (MXAN_6028/CheB5)
Myxococcus fulvus HW-1 YP_004666213
Chondromyces apiculatus DSM 436 ZP_11024979
Heliobacterium modesticaldum Ice1 YP_001680595
Geobacter sp. M18 YP_004196842
Geobacter sp. M21 YP_003022164
Halothece sp. PCC 7418 YP_007167430
Arthrospira platensis NIES-39 YP_005069487
Geitlerinema sp. PCC 7407 YP_007111209
Oscillatoria acuminata PCC 6304 YP_007085912
Oscillatoria nigro-viridis PCC 7112 YP_007114806
Microcoleus sp. PCC 7113 YP_007123431
Cyanothece sp. PCC 7822 YP_003889541
Cyanothece sp. PCC 8802 YP_003136565
Cyanothece sp. PCC 8801 YP_002371003
Crinalium epipsammum PCC 9333 YP_007140534
Chamaesiphon minutus PCC 6605 YP_007098867
Microcoleus sp. PCC 7113 YP_007121898
Stanieria cyanosphaera PCC 7437 YP_007131579
Chroococcidiopsis thermalis PCC 7203 YP_007089445
Legionella longbeachae NSW150 YP_003456747
Magnetococcus marinus MC-1 YP_865749
Desulfovibrio africanus str. Walvis Bay YP_005051440
Geobacter sp. M18 YP_004198942
Hahella chejuensis KCTC 2396 YP_434999
Pseudogulbenkiania sp. NH8B YP_004848387
Desulfomonile tiedjei DSM 6799 YP_006445055
Desulfatibacillum alkenivorans AK-01 YP_002433811
Desulfobacula toluolica Tol2 YP_006760763
Polaromonas sp. JS666 YP_549284
Nitrosospira multiformis ATCC 25196 YP_411034
Geobacter metallireducens GS-15 YP_006721666
Geobacter sp. M18 YP_004200704
Methanospirillum hungatei JF-1 YP_502457
Methanosphaerula palustris E1-9c YP_002466386
Desulfovibrio salexigens DSM 2638 YP_002990892
Desulfovibrio hydrothermalis AM13 = DSM 14728 YP_007327255
Starkeya novella DSM 506 YP_003693909
Geobacter sp. M18 YP_004198915
Geobacter sp. M21 YP_003022003
Geobacter bemidjiensis Bem YP_002138835
Chloroflexus sp. Y-400-fl YP_002568671
Chloroflexus aurantiacus J-10-fl YP_001634470
Roseiflexus castenholzii DSM 13941 YP_001433268
Roseiflexus sp. RS-1 YP_001275353
Gemmatimonas aurantiaca T-27 YP_002760851
Anaeromyxobacter sp. Fw109-5 YP_001379484
Anaeromyxobacter dehalogenans 2CP-1 YP_002493177
Anaeromyxobacter dehalogenans 2CP-C YP_464403
Anaeromyxobacter sp. K YP_002135033
Cystobacter fuscus DSM 2262 ZP_21236411
Stigmatella aurantiaca DW4_3-1 YP_003950501
Corallococcus coralloides DSM 2259 YP_005373473
Myxococcus stipitatus DSM 14675 YP_007364520
Chondromyces apiculatus DSM 436 ZP_11022999
Myxococcus xanthus DK 1622 YP_635067 (MXAN_6952/CheB6)
Rhodospirillum centenum SW YP_002298327
Magnetospirillum magneticum AMB-1 YP_421329
Azospirillum brasilense Sp245 YP_004985233
Azospirillum lipoferum 4B YP_004974652
Azospirillum sp. B510 YP_003450386
Gluconacetobacter xylinus NBRC 3288 YP_004867740
Gluconacetobacter diazotrophicus PAl 5 YP_002277424
Gluconacetobacter diazotrophicus PAl 5 YP_001603519
Phenylobacterium zucineum HLK1 YP_002129538
Methylobacterium sp. 4-46 YP_001767250
Methylobacterium nodulans ORS 2060 YP_002500601
Methylobacterium radiotolerans JCM 2831 YP_001754865
Methylobacterium populi BJ001 YP_001925648
Methylobacterium extorquens PA1 YP_001640301
Methylobacterium extorquens CM4 YP_002421832
Methylobacterium extorquens DM4 YP_003069100
Methylobacterium extorquens AM1 YP_002964067
0.2
Actinobacteria
Nitrospirae
Chloroflexi
Deltaproteobacteria
Actinobacteria
Deltaproteobacteria
Gammaproteobacteria
Alphaproteobacteria
Deltaproteobacteria
Alphaproteobacteria
Deltaproteobacteria
Firmicute
Cyanobacteria
Alphaproteobacteria
Betaproteobacteria
Euryarchaeota
Chloroflexi
Gemmatimonadetes
Deltaproteobacteria
Alphaproteobacteria
CheB methylesterase
Response regulator receiver domain
Type II/IV secretion system protein

F

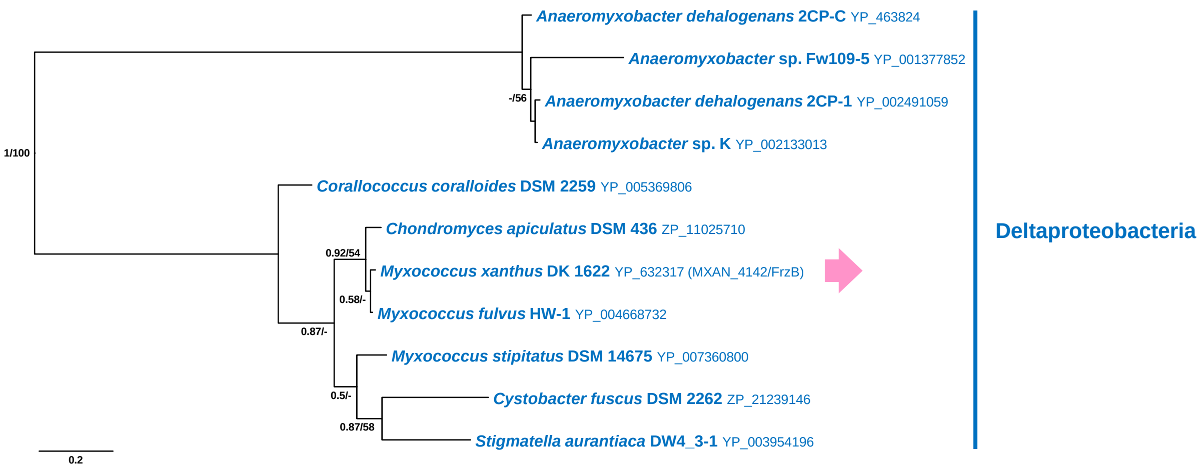
Anaeromyxobacter dehalogenans 2CP-C YP_463824
Anaeromyxobacter sp. Fw109-5 YP_001377852
Anaeromyxobacter dehalogenans 2CP-1 YP_002491059
Anaeromyxobacter sp. K YP_002133013
Corallococcus coralloides DSM 2259 YP_005369806
Chondromyces apiculatus DSM 436 ZP_11025710
Myxococcus xanthus DK 1622 YP_632317 (MXAN_4142/FrzB)
Myxococcus fulvus HW-1 YP_004668732
Myxococcus stipitatus DSM 14675 YP_007360800
Cystobacter fuscus DSM 2262 ZP_21239146
Stigmatella aurantiaca DW4_3-1 YP_003954196
Deltaproteobacteria
1/100
-/56
0.92/54
0.58/-
0.87/-
0.5/-
0.87/58
0.2

G

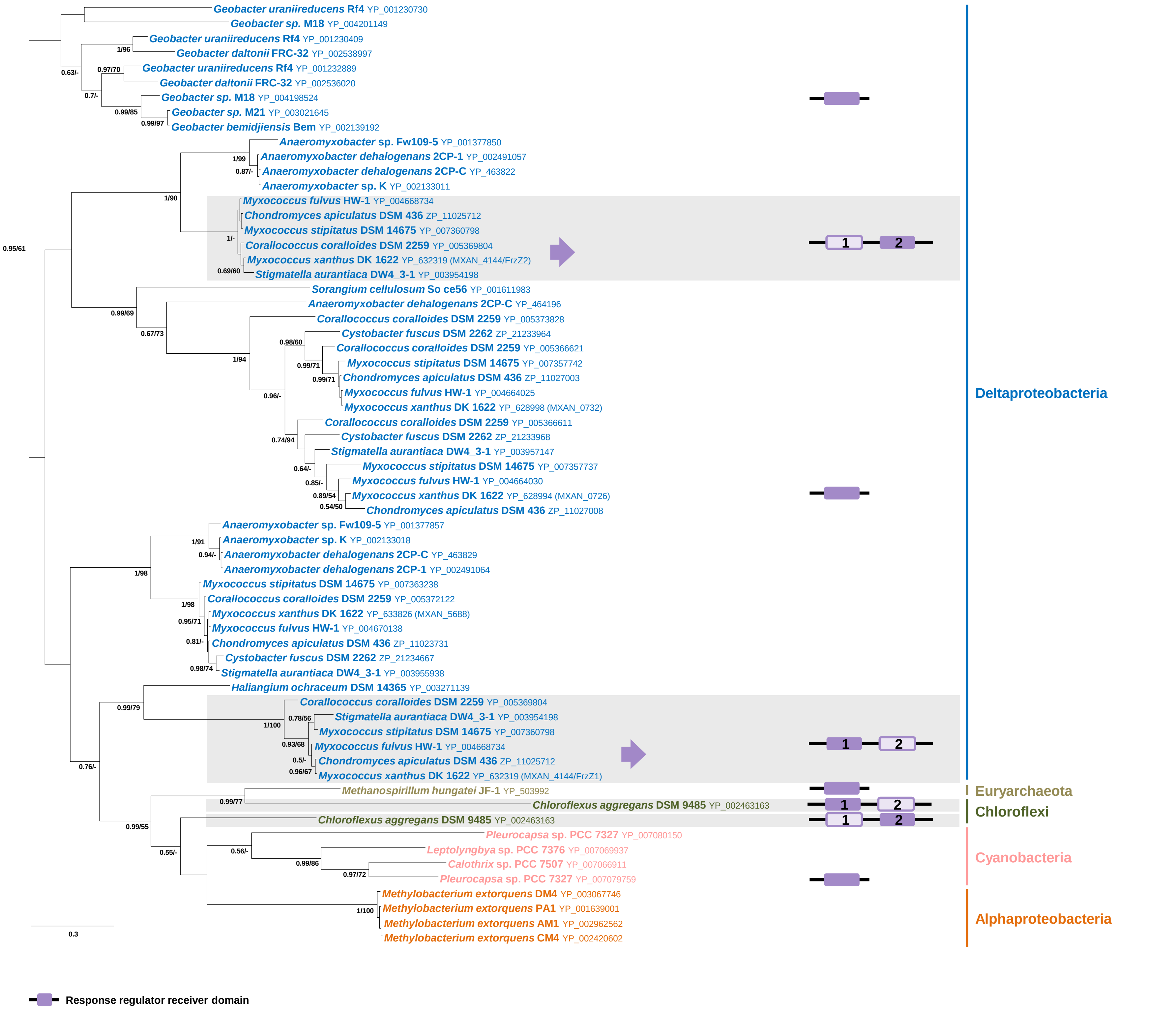
Geobacter uraniireducens Rf4 YP_001230730
Geobacter sp. M18 YP_004201149
Geobacter uraniireducens Rf4 YP_001230409
Geobacter daltonii FRC-32 YP_002538997
Geobacter uraniireducens Rf4 YP_001232889
Geobacter daltonii FRC-32 YP_002536020
Geobacter sp. M18 YP_004198524
Geobacter sp. M21 YP_003021645
Geobacter bemidjiensis Bem YP_002139192
Anaeromyxobacter sp. Fw109-5 YP_001377850
Anaeromyxobacter dehalogenans 2CP-1 YP_002491057
Anaeromyxobacter dehalogenans 2CP-C YP_463822
Anaeromyxobacter sp. K YP_002133011
Myxococcus fulvus HW-1 YP_004668734
Chondromyces apiculatus DSM 436 ZP_11025712
Myxococcus stipitatus DSM 14675 YP_007360798
Corallococcus coralloides DSM 2259 YP_005369804
Myxococcus xanthus DK 1622 YP_632319 (MXAN_4144/FrzZ2)
Stigmatella aurantiaca DW4_3-1 YP_003954198
Sorangium cellulosum So ce56 YP_001611983
Anaeromyxobacter dehalogenans 2CP-C YP_464196
Corallococcus coralloides DSM 2259 YP_005373828
Cystobacter fuscus DSM 2262 ZP_21233964
Corallococcus coralloides DSM 2259 YP_005366621
Myxococcus stipitatus DSM 14675 YP_007357742
Chondromyces apiculatus DSM 436 ZP_11027003
Myxococcus fulvus HW-1 YP_004664025
Myxococcus xanthus DK 1622 YP_628998 (MXAN_0732)
Corallococcus coralloides DSM 2259 YP_005366611
Cystobacter fuscus DSM 2262 ZP_21233968
Stigmatella aurantiaca DW4_3-1 YP_003957147
Myxococcus stipitatus DSM 14675 YP_007357737
Myxococcus fulvus HW-1 YP_004664030
Myxococcus xanthus DK 1622 YP_628994 (MXAN_0726)
Chondromyces apiculatus DSM 436 ZP_11027008
Anaeromyxobacter sp. Fw109-5 YP_001377857
Anaeromyxobacter sp. K YP_002133018
Anaeromyxobacter dehalogenans 2CP-C YP_463829
Anaeromyxobacter dehalogenans 2CP-1 YP_002491064
Myxococcus stipitatus DSM 14675 YP_007363238
Corallococcus coralloides DSM 2259 YP_005372122
Myxococcus xanthus DK 1622 YP_633826 (MXAN_5688)
Myxococcus fulvus HW-1 YP_004670138
Chondromyces apiculatus DSM 436 ZP_11023731
Cystobacter fuscus DSM 2262 ZP_21234667
Stigmatella aurantiaca DW4_3-1 YP_003955938
Haliangium ochraceum DSM 14365 YP_003271139
Corallococcus coralloides DSM 2259 YP_005369804
Stigmatella aurantiaca DW4_3-1 YP_003954198
Myxococcus stipitatus DSM 14675 YP_007360798
Myxococcus fulvus HW-1 YP_004668734
Chondromyces apiculatus DSM 436 ZP_11025712
Myxococcus xanthus DK 1622 YP_632319 (MXAN_4144/FrzZ1)
Methanospirillum hungatei JF-1 YP_503992
Chloroflexus aggregans DSM 9485 YP_002463163
Chloroflexus aggregans DSM 9485 YP_002463163
Pleurocapsa sp. PCC 7327 YP_007080150
Leptolyngbya sp. PCC 7376 YP_007069937
Calothrix sp. PCC 7507 YP_007066911
Pleurocapsa sp. PCC 7327 YP_007079759
Methylobacterium extorquens DM4 YP_003067746
Methylobacterium extorquens PA1 YP_001639001
Methylobacterium extorquens AM1 YP_002962562
Methylobacterium extorquens CM4 YP_002420602
Deltaproteobacteria
Euryarchaeota
Chloroflexi
Cyanobacteria
Alphaproteobacteria
0.3
Response regulator receiver domain
