# Supplementary material for: Evolution and Design Governing Signal Precision and Amplification in a Bacterial Chemosensory Pathway
Source: PLoS Genet. 2015 Aug 20;11(8):e1005460. doi: 10.1371/journal.pgen.1005460 (PMC4546325; doi:10.1371/journal.pgen.1005460)

Myxococcus xanthus DK 1622, *Chondromyces apiculatus* DSM 436, *Corallococcus coralloides* DSM 2259, *Stigmatella aurantiaca* DW4_3-1, *Cystobacter fuscus* DSM 2262, *Myxococcus fulvus* HW-1, *Myxococcus stipitatus* DSM 14675 - **Deltaproteobacteria**

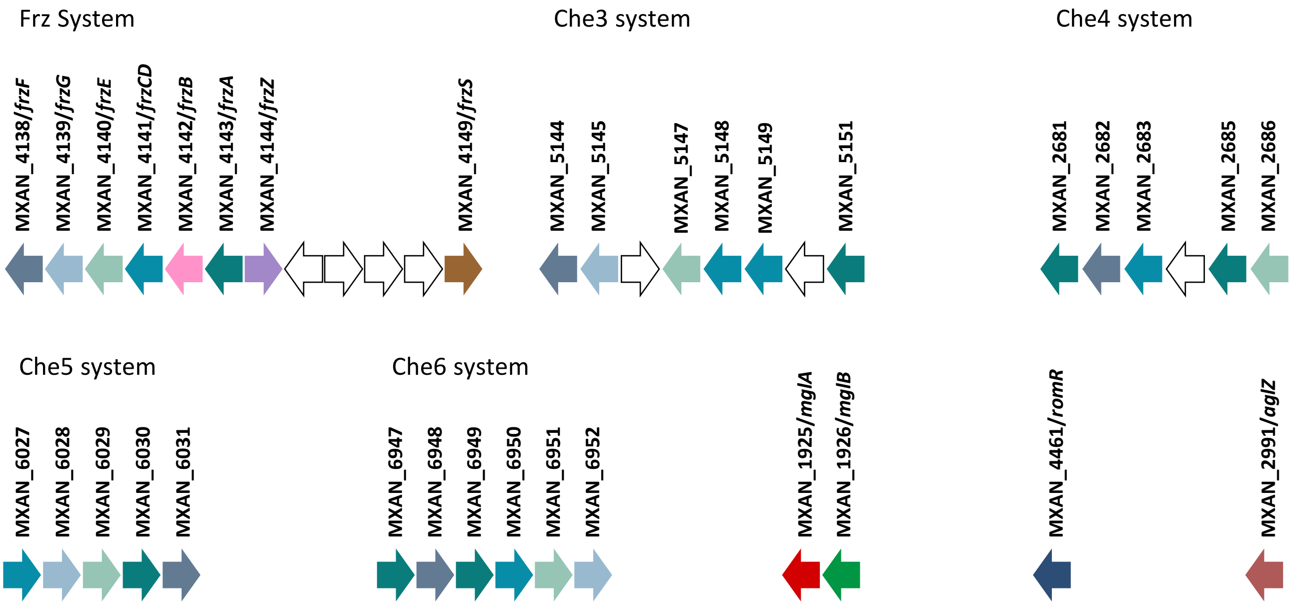

Gemmatimonas aurantiaca T-27 - **Gemmatimonadetes**

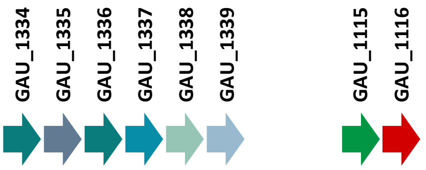

Anaeromyxobacter sp. Fw109-5, *Anaeromyxobacter dehalogenans* 2CP-C, *Anaeromyxobacter dehalogenans* 2CP-1, *Anaeromyxobacter* sp. K - **Deltaproteobacteria**

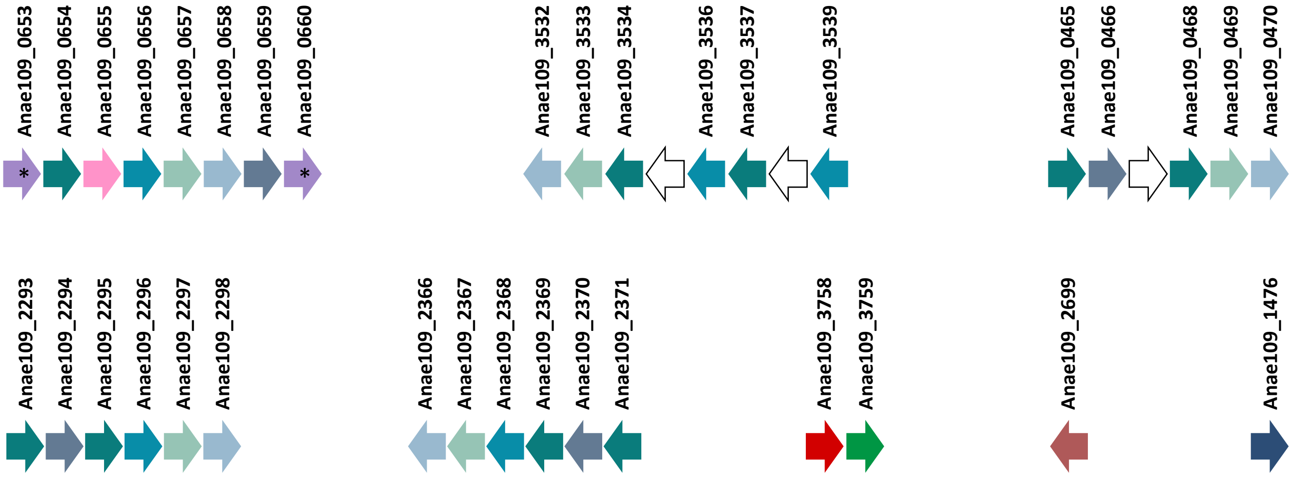

Haliangium ochraceum DSM14365 - **Deltaproteobacteria**

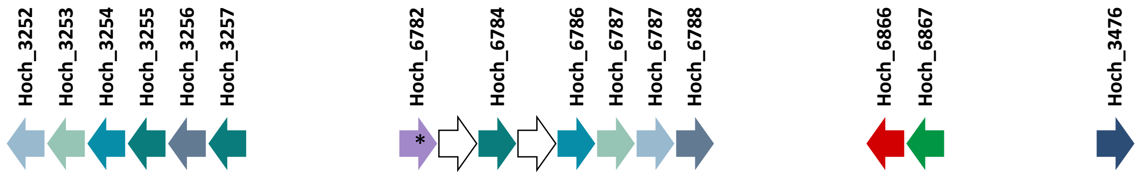

Geobacter sp. M18, *Geobacter* sp. M21 - **Deltaproteobacteria**

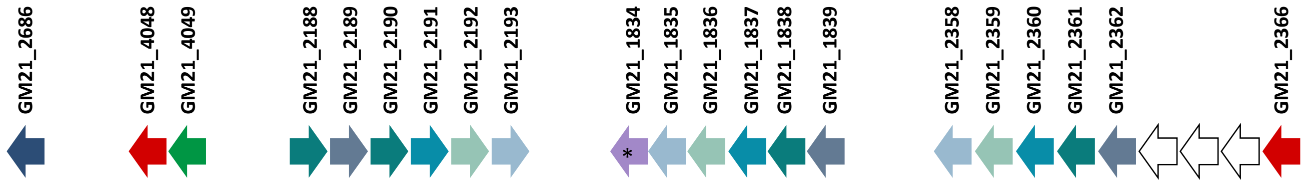

Chloroflexus aggregans DSM 9485, *Roseiflexus castenholzii* DSM 13941, *Roseiflexus* sp. RS-1, *Chloroflexus aurantiacus* J-10-fl, *Chloroflexus* sp. Y-400-fl - **Chloroflexi**

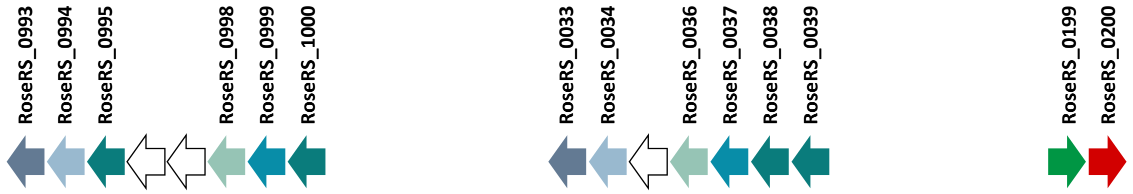

Nitrosospira multiformis ATCC 25196 - **Bettaproteobacteria**

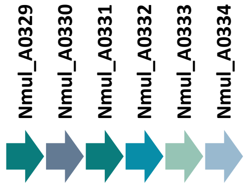

Methanospirillum hungatei JF-1 - **Euryarchaeota**

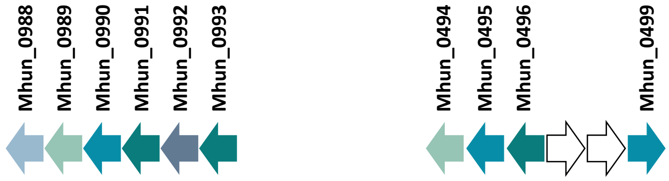

Cyanothece sp. PCC 8802 - **Cyanobacteria**

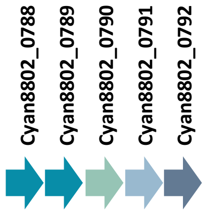

Sinorhizobium meliloti SM11, *Sinorhizobium fredii* NGR234 - **Alphaproteobacteria**

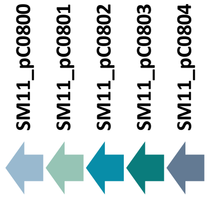

Gluconacetobacter diazotrophicus PAl 5 - **Alphaproteobacteria**

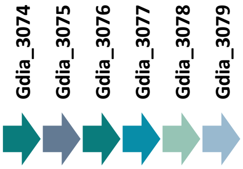

Acidothermus cellulolyticus 11B - **Actinobactria**

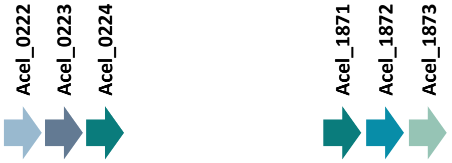

-*Magnetococcus marinus* MC-1 - **Proteobacteria**

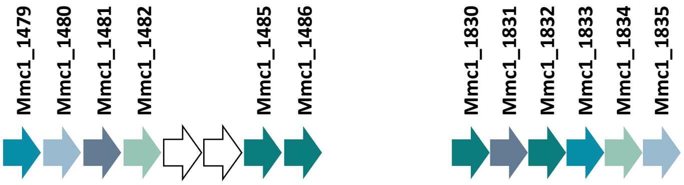

Hahella chejuensis KCTC 2396 - **Gammaproteobacteria**

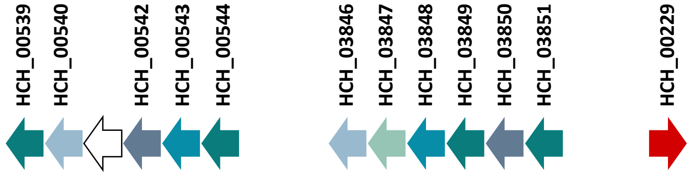

Supplement: S5 Fig — The same color code as in Fig 1 is used. Locus_tags are shown for all genes. White arrows indicate genes encoding for proteins that are not related to the Frz system or motility. The frzZ homologues containing only one response regulator domains are indicated with an asterisk. (PDF) [file pgen.1005460.s005.pdf]
