# Supplementary material for: Evolution and Design Governing Signal Precision and Amplification in a Bacterial Chemosensory Pathway
Source: PLoS Genet. 2015 Aug 20;11(8):e1005460. doi: 10.1371/journal.pgen.1005460 (PMC4546325; doi:10.1371/journal.pgen.1005460)
Supplement: S7 Fig — The positions of the phosphorylation site (D57 in the canonical CheY domain), the residues that chelates the Mg2+necessary for aspartic acid phosphorylation (D12 and D13 in the canonical CheY domain) and the residues involved in a shift of the hydrogen-bonding network (T87, Y106 and K109 in the canonical CheY domain) are indicated in red, grey and black, respectively. (PDF) [file pgen.1005460.s007.pdf]

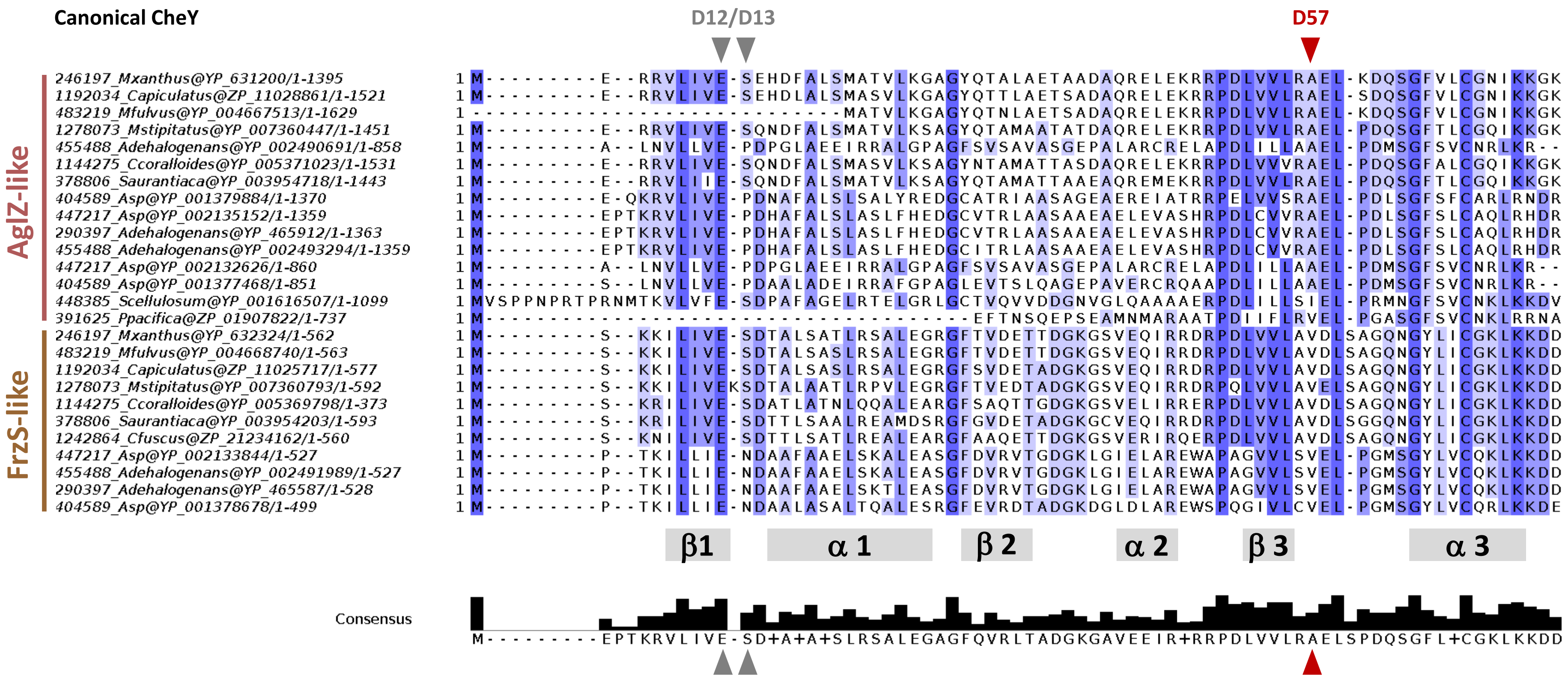

Canonical CheY
D12/D13
D57
AglZ-like
FrzS-like
246197_Mxanthus@YP_631200/1-1395
1192034_Capiculatus@ZP_11028861/1-1521
483219_Mfulvus@YP_004667513/1-1629
1278073_Mstipitatus@YP_007360447/1-1451
455488_Adehalogenans@YP_002490691/1-858
1144275_Ccoralloides@YP_005371023/1-1531
378806_Saurantiaca@YP_003954718/1-1443
404589_Asp@YP_001379884/1-1370
447217_Asp@YP_002135152/1-1359
290397_Adehalogenans@YP_465912/1-1363
455488_Adehalogenans@YP_002493294/1-1359
447217_Asp@YP_002132626/1-860
404589_Asp@YP_001377468/1-851
448385_Scellulosum@YP_001616507/1-1099
391625_Ppacifica@ZP_01907822/1-737
246197_Mxanthus@YP_632324/1-562
483219_Mfulvus@YP_004668740/1-563
1192034_Capiculatus@ZP_11025717/1-577
1278073_Mstipitatus@YP_007360793/1-592
1144275_Ccoralloides@YP_005369798/1-373
378806_Saurantiaca@YP_003954203/1-593
1242864_Cfuscus@ZP_21234162/1-560
447217_Asp@YP_002133844/1-527
455488_Adehalogenans@YP_002491989/1-527
290397_Adehalogenans@YP_465587/1-528
404589_Asp@YP_001378678/1-499
β1
α 1
β 2
α 2
β 3
α 3
Consensus

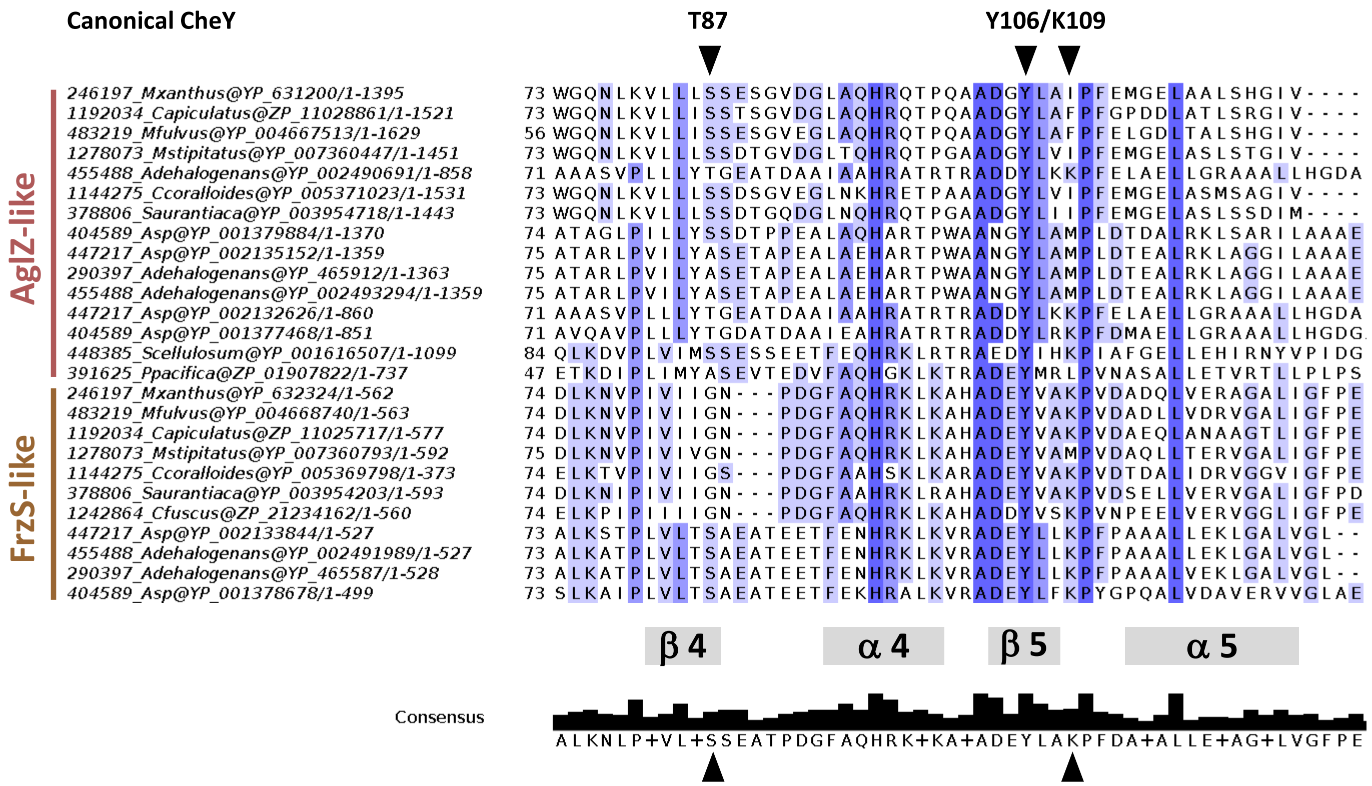
Canonical CheY
T87
Y106/K109
AglZ-like
FrzS-like
246197_Mxanthus@YP_631200/1-1395 73 WGQNLKVLLLSSESGVDGLAQHRQTPQAADGYLAIPFEMGELAALSHGIV----
1192034_Capiculatus@ZP_11028861/1-1521 73 WGQNLKVLLISSTSGVDGLAQHRQTPQAADGYLAFPFGPDDLATLSRGIV----
483219_Mfulvus@YP_004667513/1-1629 56 WGQNLKVLLISSESGVEGLAQHRQTPQAADGYLAFPFELGDLTALSHGIV----
1278073_Mstipitatus@YP_007360447/1-1451 73 WGQNLKVLLLSSDTGVDGLTQHRQTPGAADGYLVIPFEMGELASLSTGIV----
455488_Adehalogenans@YP_002490691/1-858 71 AAASVPLLLYTGEATDAAIAAHRATRTRADDYLKKPFELAELLGRAAALLHGDA
1144275_Ccoralloides@YP_005371023/1-1531 73 WGQNLKVLLLSSDSGVEGLNKHRETPAAADGYLVIPFEMGELASMSAGIV----
378806_Saurantiaca@YP_003954718/1-1443 73 WGQNLKVLLLSSDTGQDGLNQHRQTPGAADGYLIIPFEMGELASLSSDIM----
404589_Asp@YP_001379884/1-1370 74 ATAGLPILLYSSDTPPEALAQHARTPWAANGYLAMPLDTDALRKLSARILAAAE
447217_Asp@YP_002135152/1-1359 75 ATARLPVILYASETAPEALAEHARTPWAANGYLAMPLDTEALRKLAGGILAAAE
290397_Adehalogenans@YP_465912/1-1363 75 ATARLPVILYASETAPEALAEHARTPWAANGYLAMPLDTEALRKLAGGILAAAE
455488_Adehalogenans@YP_002493294/1-1359 75 ATARLPVILYASETAPEALAEHARTPWAANGYLAMPLDTEALRKLAGGILAAAE
447217_Asp@YP_002132626/1-860 71 AAASVPLLLYTGEATDAAIAAHRATRTRADDYLKKPFELAELLGRAAALLHGDA
404589_Asp@YP_001377468/1-851 71 AVQAVPLLLYTGDATDAAIEAHRATRTRADDYLRKPFDMAELLGRAAALLHGDG
448385_Scellulosum@YP_001616507/1-1099 84 QLKDVPLVIMSSESSEETFEQHRKLRTRAEDYIHKPIAFGELLEHIRNYVPIDG
391625_Ppacifica@ZP_01907822/1-737 47 ETKDIPLIMYASEVTEDVFAQHGKLKTRADEYMRLPVNASALLETVRTLLPLPS
246197_Mxanthus@YP_632324/1-562 74 DLKNVPIVIIGN---PDGFAQHRKLKAHADEYVAKPVDADQLVERAGALIGFPE
483219_Mfulvus@YP_004668740/1-563 74 DLKNVPIVIIGN---PDGFAQHRKLKAHADEYVAKPVDADLLVDRVGALIGFPE
1192034_Capiculatus@ZP_11025717/1-577 74 DLKNVPIVIIGN---PDGFAQHRKLKAHADEYVAKPVDAEQLANAAGTLIGFPE
1278073_Mstipitatus@YP_007360793/1-592 75 DLKNVPIVIVGN---PDGFAQHRKLKAHADEYVAMPVDAQLLTERVGALIGFPE
1144275_Ccoralloides@YP_005369798/1-373 74 ELKTVPIVIIGS---PDGFAAHSKLKARADEYVAKPVDTDALIDRVGGVIGFPE
378806_Saurantiaca@YP_003954203/1-593 74 DLKNIPIVIIGN---PDGFAAHRKLRAHADEYVAKPVDSELLVERVGALIGFPD
1242864_Cfuscus@ZP_21234162/1-560 74 ELKPIPIIIIGN---PDGFAQHRKLKAHADDYVSKPVNPEELVERVGGLIGFPE
447217_Asp@YP_002133844/1-527 73 ALKSTPLVLTSAEATEETFENHRKLKVRADEYLLKPFPAAALLEKLGALVGL--
455488_Adehalogenans@YP_002491989/1-527 73 ALKATPLVLTSAEATEETFENHRKLKVRADEYLLKPFPAAALLEKLGALVGL--
290397_Adehalogenans@YP_465587/1-528 73 ALKATPLVLTSAEATEETFENHRKLKVRADEYLLKPFPAAALVEKLGALVGL--
404589_Asp@YP_001378678/1-499 73 SLKAIPLVLTSAEATEETFEKHRALKVRADEYLFKPYGPQALVDAVERVVGLAE
β 4
α 4
β 5
α 5
Consensus
ALKNLP+VL+SSEATPDGFAQHRK+KA+ADEYLAKPFDA+ALLE+AG+LVGFPE
