# Supplementary material for: Evolution and Design Governing Signal Precision and Amplification in a Bacterial Chemosensory Pathway
Source: PLoS Genet. 2015 Aug 20;11(8):e1005460. doi: 10.1371/journal.pgen.1005460 (PMC4546325; doi:10.1371/journal.pgen.1005460)
Supplement: S8 Fig — (A) Single cell Tfp-dependent reversal. Shown is a WT cell. Time frame: 15s, scale bar = 2μm. (B) Tracking of Tfp-dependent reversals. Selected timelapses are subjected to automated cell detection and tracking using a homegrown procedure under Fiji (Image J/NIH). For each cell the algorithm records the velocity (green), the cumulated traveled distance (blue) and count reversals (red), which is used to calculate the reversal frequency. (PDF) [file pgen.1005460.s008.pdf]

A

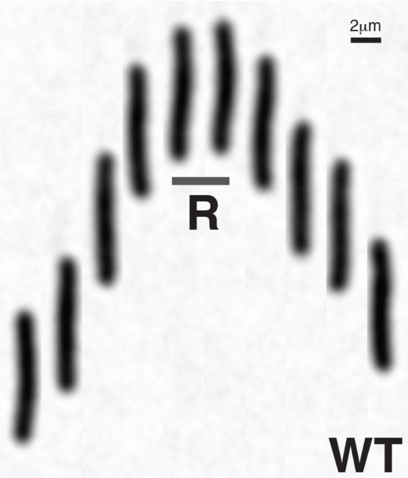

B

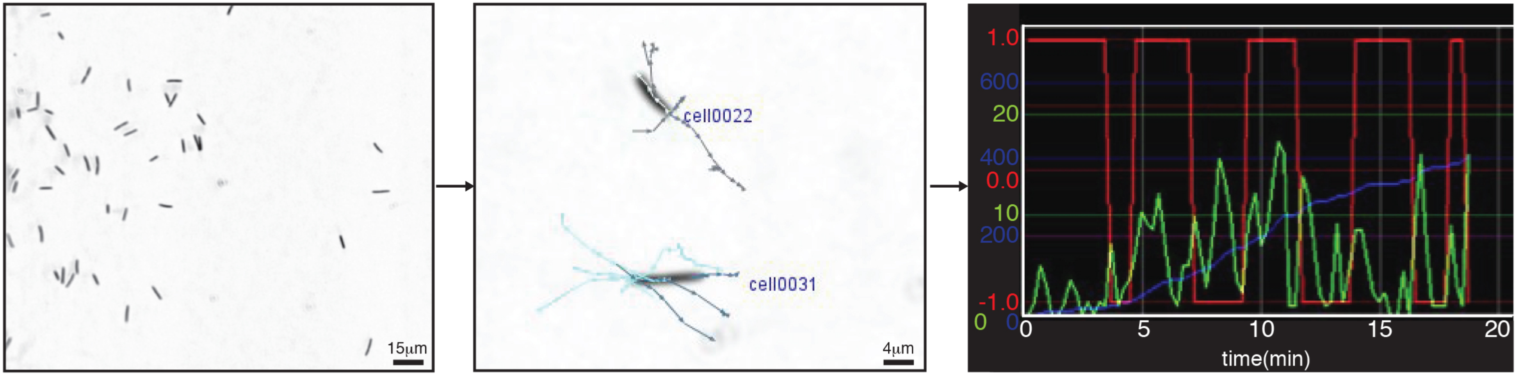

Timelapse on carboxymethylcellulose

Automated cell detection and tracking

Automated cell directional changes detection
