# Supplementary material for: Evolution and Design Governing Signal Precision and Amplification in a Bacterial Chemosensory Pathway
Source: PLoS Genet. 2015 Aug 20;11(8):e1005460. doi: 10.1371/journal.pgen.1005460 (PMC4546325; doi:10.1371/journal.pgen.1005460)
Supplement: S1 Table — (DOCX) [file pgen.1005460.s013.docx]

**Table S1: Bacterial strains used in this study**

| Table S1. *Myxococcus* strains | | | |
| --- | --- | --- | --- |
| Strain | Construction | Genotype or Description | Source or Reference |
| DZ2  DZ4038  DZ4479  DZ4480  DZ4481  DZ4484  DZ4547  DZ4624  DZ4703  DZ4770  TM3  TM4  TM146  TM184  TM254  TM389  TM399  TM400  TM586  TM844  TM856  TM857  TM860  TM871  TM873  TM876  TM877  TM880  TM932  TM941  TM965  TM973  EM213 | Wild type  DZ2 *frz^on^*  DZ2 Δ*frzB*  DZ2 Δ*frzCD*  DZ2 Δ*frzE*  DZ2 Δ*frzZ*  DZ2 *frzE*^RR^  DZ4547 *frzS-gfp*  DZ4547 Δ*frzZ*  DZ2 Δ*aglZ*  DZ2 *frzS-gfp*  DZ2 Δ*frzE*  *frzS-gfp*  DZ2 Δ*aglQ*  DZ2 MglA-YFP *mx8att::mglA*  DZ2 Δ*romR*  DZ2 Δ*pilA*  DZ2 Δ*romR* *frzS-yfp*  DZ2 Δ*romR mglA-yfp mglA*  DZ2 *frz^on^ ΔromR* (pBJ114 Δ*romR*)  DZ4484 *ΔromR* (pBJ114 Δ*romR*)  DZ4479 Δ*aglZ* (pBJ114 Δ*aglZ)*  TM30 Δ*frzZ* (pBJ114 Δ*frzZ*)  TM30 Δ*romR* (pBJ114 Δ*romR*)  DZ4038 Δ*frzZ* (pBJ114 Δ*frzZ*)  TM856 Δ*frzZ* (pBJ114 Δ*frzZ*)  TM860 *frzS-yfp* (pEFrzSY)  DZ4484 *frzS-yfp* (pEFrzSY)  TM844 *frzS-yfp* (pEFrzSY)  TM857 *frzS-yfp* (pEFrzSY)  TM890 *frzS-yfp* (pEFrzSY)  DZ4038 *frzE*^RR^ (pBJ114 *frzE*^RR^)  TM873 Δ*frzE* (pBJ114 Δ*frzE*)  DZ2 *lacI::frzCD669* (pEM140) | WT  *frz^on^*  Δ*frzB*  Δ*frzCD*  Δ*frzE*  Δ*frzZ*  *frzE*^RR^  *frzE*^RR^ *frzS-gfp*  *frzE*^RR^ Δ*frzZ*  Δ*aglZ*  *frzS-gfp*  Δ*frzE frzS-gfp*  Δ*aglQ*  *mglA-yfp mglA*  Δ*romR*  Δ*pilA*  Δ*romR frzS-yfp*  Δ*romR mglA-yfp mglA^+^*  *frz^on^* Δ*romR*  Δ*frzZ* Δ*romR*  Δ*frzB* Δ*aglZ*  Δ*frzE* Δ*frzZ*  Δ*frzE* Δ*romR*  *frz^on^* Δ*frzZ*  Δ*frzB* Δ*aglZ* Δ*frzZ*  Δ*frzE* Δ*romR frzS-yfp*  Δ*frzZ frzS-yfp*  Δ*frzZ* Δ*romR frzS-yfp*  Δ*frzE* Δ*frzZ frzS-yfp*  *frzE*^RR^ Δ*frzZ frzS-yfp*  *frz^on^ frzE*^RR^  Δ*frzB* Δ*aglZ* Δ*frzZ* Δ*frzE*  *lacI::frzCD669* | Laboratory collection  [1]  [1]  [1]  [1]  [1]  [2]  [3]  [4]  [5]  [6]  [6]  [7]  [8]  [9]  Laboratory collection  [9]  [9]  This work  This work  This work  This work  This work  This work  This work  This work  This work  This work  This work  This work  This work  This work  This work |
