# Supplementary material for: Evolution and Design Governing Signal Precision and Amplification in a Bacterial Chemosensory Pathway
Source: PLoS Genet. 2015 Aug 20;11(8):e1005460. doi: 10.1371/journal.pgen.1005460 (PMC4546325; doi:10.1371/journal.pgen.1005460)
Supplement: S2 Table — (DOCX) [file pgen.1005460.s014.docx]

**Table S2: Plasmids used in this study**

| Table S2. Plasmids |  |  |
| --- | --- | --- |
| Name | Description | Source |
| pBJ114  pEFrzSY  pBJ114 Δ*romR*  pBJ114 Δ*aglZ*  pBJ114 Δ*frzZ*  pBJ114 *frzE*^RR^  pBJ114 Δ*frzE*  pBBR1MCS  pEM143  pETPhos  pGEX(M)  pETPhos_*frzE^kinase^*  pETPhos_*frzCD*  pETPhos_*frzCD^c^*  pGEX(M)_*frzA* | Used to create deletions, *galK*, Km^R^  pEYFPN1 with a cassette allowing construction of the *frzS-yfp* chimeric gene  pBJ114 with a deletion cassette for *romR*  pBJ114 with a deletion cassette for *aglZ*  pBJ114 with a deletion cassette for *frzZ*  pBJ114 with a deletion cassette for *frzE*^RR^  pBJ114 with a deletion cassette for *frzE*  pAK20 derivative  Derivative of pBBR1MCS ( carrying the lacI gene under its promoter and followed by the first 669 of *frzCD* (SpeI/EcoRI)  pET-15b (Novagen) derivative including the replacement of the thrombin site coding sequence with a tobacco etch virus (TEV) protease site and Ser to Gly mutagenesis in the Nterm His-tag  pGEX derivative with a 321-bp EcoRI/BamHI fragment from pET19b introducing a HindIII site in the pGEX polylinker  pETPhos derivative used to express His-tagged fusion of *Myxococcus xanthus* FrzE^kinase^ in *E. coli*  pETPhos derivative used to express His-tagged fusion of*Myxococcus xanthus* FrzCD in *E. coli*  pETPhos derivative used to express His-tagged fusion of  *Myxococcus xanthus* FrzCD^c^ in *E. coli*  pGEX(M) derivative used to express GST-tagged fusion of  *Myxococcus xanthus* FrzA in *E. coli* | Laboratory collection  Laboratory collection  [9]  [5]  [1]  [2]  [1]  Arash Komeili, unpublished  This work  This work  [10]  This work  This work  This work  This work |
