# Supplementary material for: Evolution and Design Governing Signal Precision and Amplification in a Bacterial Chemosensory Pathway
Source: PLoS Genet. 2015 Aug 20;11(8):e1005460. doi: 10.1371/journal.pgen.1005460 (PMC4546325; doi:10.1371/journal.pgen.1005460)
Supplement: S3 Table — (DOCX) [file pgen.1005460.s015.docx]

**Table S3: Primers used in this study**

| Table S3. Primers | |  |
| --- | --- | --- |
| Plasmids | Name | Sequences of primers (5’---3’) |
| pETPhos Nterm FrzE^kinase^  pETPhos Cterm FrzE^kinase^  pETPhos Nterm FrzCD  pETPhos Cterm FrzCD  pETPhos Nterm FrzCD^c^  pETPhos Cterm FrzCD^c^  pGEX Nterm FrzA  pGEX Cterm FrzA  pEM143 | *frzE^kinase^*  *frzE^kinase^*  *frzCD*  *frzCD*  *frzCD^c^*  *frzCD^c^*  *frzA*  *frzA*  CDind1  CDind2 | TAATAGctCATATGgacaccgaggctctcaagaaatcc (*NdeI*)  TATGGATCCctagcgcttggcggcgggggcctg *(BamHI)*  TAATAGCTCATATGTCCCTGGACACCCCCAAC *(NdeI)*  TATGGATCCctagtcggccttgaaccgctt (*BamHI*)  ATTTCCAGGGCCATATGTCCCTGGACACCCCCAACGAG (*Nde*I)  TTAGTTATTAGGATCCCTGCTTCGAGGCCTGCGTCGT (*BamHI*)  TATGGATCCATGgctccggaccgcgccttg (*BamHI*)  TATAAGCTTTCAccgcgccaccgcccgctg (*HindIII*)  gaattcATGTCCCTGGACACCCCCAACGA (*EcoRI*)  actagtcatggcctggatgaactcgccaat (*SpeI*) |
