# Supplementary material for: Evolution and Design Governing Signal Precision and Amplification in a Bacterial Chemosensory Pathway
Source: PLoS Genet. 2015 Aug 20;11(8):e1005460. doi: 10.1371/journal.pgen.1005460 (PMC4546325; doi:10.1371/journal.pgen.1005460)
Supplement: S4 Table — (PDF) [file pgen.1005460.s016.pdf]

FrzCD domain

GI	Organis	Refseq	pfam				Transm
56780	Saciditrop hicus	YP_4625 14	Cache domain	HAMP domain	Methyl-accepting chemotax is protein (MCP) signalling domain		2
56780	Saciditrop hicus	YP_4607 38	Cache domain	HAMP domain	Methyl-accepting chemotax is protein (MCP) signalling domain		2
378806	Saurantia ca	YP_0039 50119	CHASE3 domain	Methyl-accepting chemotax is protein (MCP) signalling domain			2
1242864	Cfuscus	ZP_2123 9366	CHASE3 domain	Methyl-accepting chemotax is protein (MCP) signalling domain			2
443143	Gsp	YP_0041 98527	CHASE3 domain	Methyl-accepting chemotax is protein (MCP) signalling domain			2

				FrzCD domain	
1173022	Cepipsammum	YP_007140544	CHASE3 domain	Methyl-accepting chemotaxis protein (MCP) signalling domain	2
395962	Csp	YP_003136562	CHASE3 domain	Methyl-accepting chemotaxis protein (MCP) signalling domain	2
41431	Csp	YP_002371000	CHASE3 domain	Methyl-accepting chemotaxis protein (MCP) signalling domain	2
696747	Aplatensis	YP_005069489	CHASE3 domain	Methyl-accepting chemotaxis protein (MCP) signalling domain	2
56110	Oacuminata	YP_007087656	CHASE3 domain	Methyl-accepting chemotaxis protein (MCP) signalling domain	2

				FrzCD domain			
1173025	Gsp	YP_007111207	CHASE3 domain	Methyl-accepting chemotaxis protein (MCP) signalling domain			2
1173025	Gsp	YP_007109426	CHASE3 domain	Methyl-accepting chemotaxis protein (MCP) signalling domain			2
1173025	Gsp	YP_007109427	CHASE3 domain	Methyl-accepting chemotaxis protein (MCP) signalling domain			2
480224	Csp	YP_002568675	CHASE3 domain	HAMP domain	Methyl-accepting chemotaxis protein (MCP) signalling domain		1
706587	Dtiedjei	YP_006445913	CHASE3 domain	CHASE3 domain	HAMP domain	Methyl-accepting chemotaxis protein (MCP) signalling domain	2

FrzCD domain

706587	Dtiedjei	YP_006445914	CHASE3 domain	CHASE3 domain	HAMP domain	Methyl-accepting chemotaxis protein (MCP) signalling domain	2
706587	Dtiedjei	YP_006445300	CHASE3 domain	CHASE3 domain	HAMP domain	Methyl-accepting chemotaxis protein (MCP) signalling domain	2
296591	Psp	YP_549281	CHASE3 domain	HAMP domain	Methyl-accepting chemotaxis protein (MCP) signalling domain		2
1242864	Cfuscus	ZP_21238041	CHASE3 domain	HAMP domain	Methyl-accepting chemotaxis protein (MCP) signalling domain		2
426355	Mradiotolerans	YP_001755683	CHASE3 domain	HAMP domain	Methyl-accepting chemotaxis protein (MCP) signalling domain		2

497965	Csp	YP_003889537	Four helix bundle sensory module for signal transduction	Methyl-accepting chemotaxis protein (MCP) signalling domain			2
696747	Aplatensis	YP_005069490	Four helix bundle sensory module for signal transduction	Methyl-accepting chemotaxis protein (MCP) signalling domain			2
357808	Rsp	YP_001275358	Four helix bundle sensory module for signal transduction	HAMP domain	Methyl-accepting chemotaxis protein (MCP) signalling domain		2
383372	Rcastenholzii	YP_001433273	Four helix bundle sensory module for signal transduction	HAMP domain	Methyl-accepting chemotaxis protein (MCP) signalling domain		2
1121451	Dhydrothermalis	YP_007327514	Four helix bundle sensory module for signal transduction	HAMP domain	Methyl-accepting chemotaxis protein (MCP) signalling domain		2

FrzCD domain

443144	Gsp	YP_003022001	Four helix bundle sensory module for signal transduction	HAMP domain	Methyl-accepting chemotaxis protein (MCP) signalling domain		2
404380	Gbemidjiensis	YP_002138837	Four helix bundle sensory module for signal transduction	HAMP domain	HAMP domain	Methyl-accepting chemotaxis protein (MCP) signalling domain	2
443143	Gsp	YP_004198913	Four helix bundle sensory module for signal transduction	HAMP domain	HAMP domain	Methyl-accepting chemotaxis protein (MCP) signalling domain	2
323848	Nmultiformis	YP_411032	Four helix bundle sensory module for signal transduction	Methyl-accepting chemotaxis protein (MCP) signalling domain			2
269799	Gmetallireducen	YP_006721664	Four helix bundle sensory module for signal transduction	HAMP domain	Methyl-accepting chemotaxis protein (MCP) signalling domain		2

			FrzCD domain			
349521	Hchejuensis	YP_431844	Four helix bundle sensory module for signal transduction	HAMP domain	Methyl-accepting chemotaxis protein (MCP) signalling domain	1
349521	Hchejuensis	YP_435001	Four helix bundle sensory module for signal transduction	HAMP domain	Methyl-accepting chemotaxis protein (MCP) signalling domain	2
521011	Mpalustris	YP_002466384	Four helix bundle sensory module for signal transduction	HAMP domain	Methyl-accepting chemotaxis protein (MCP) signalling domain	2
323259	Mhungatei	YP_502459	Four helix bundle sensory module for signal transduction	HAMP domain	Methyl-accepting chemotaxis protein (MCP) signalling domain	2
379066	Gaurantiaca	YP_002760849	Four helix bundle sensory module for signal transduction	Methyl-accepting chemotaxis protein (MCP) signalling domain		2

FrzCD domain

502025	Hochraceum	YP_003267649	Four helix bundle sensory module for signal transduction	HAMP domain	Methyl-accepting chemotaxis protein (MCP) signalling domain	2
545694	Tprimitia	YP_004531339	HAMP domain	Methyl-accepting chemotaxis protein (MCP) signalling domain		2
572477	Avinosum	YP_003442577	HAMP domain	Methyl-accepting chemotaxis protein (MCP) signalling domain		2
156889	Mmarinus	YP_865394	HAMP domain	Methyl-accepting chemotaxis protein (MCP) signalling domain		2
1150469	Rphotometricum	YP_005416578	HAMP domain	Methyl-accepting chemotaxis protein (MCP) signalling domain		2

				FrzCD domain	
342108	Mmagneticum	YP_422364	HAMP domain	Methyl-accepting chemotaxis protein (MCP) signalling domain	1
1278073	Mstipitatus	YP_007362606	HAMP domain	Methyl-accepting chemotaxis protein (MCP) signalling domain	6
246197	Mxanthus	YP_633301	HAMP domain	Methyl-accepting chemotaxis protein (MCP) signalling domain	6
483219	Mfulvus	YP_004669701	HAMP domain	Methyl-accepting chemotaxis protein (MCP) signalling domain	7
378806	Saurantiaca	YP_003955505	HAMP domain	Methyl-accepting chemotaxis protein (MCP) signalling domain	5

				FrzCD domain	
1242864	Cfuscus	ZP_21229930	HAMP domain	Methyl-accepting chemotaxis protein (MCP) signalling domain	6
1144275	Ccoralloides	YP_005368337	HAMP domain	Methyl-accepting chemotaxis protein (MCP) signalling domain	6
1144275	Ccoralloides	YP_005368336	HAMP domain	Methyl-accepting chemotaxis protein (MCP) signalling domain	6
1278073	Mstipitatus	YP_007362607	HAMP domain	Methyl-accepting chemotaxis protein (MCP) signalling domain	5
1192034	Capiculatus	ZP_11023349	HAMP domain	Methyl-accepting chemotaxis protein (MCP) signalling domain	6

				FrzCD domain	
246197	Mxanthus	YP_633302	HAMP domain	Methyl-accepting chemotaxis protein (MCP) signalling domain	6
483219	Mfulvus	YP_004669702	HAMP domain	Methyl-accepting chemotaxis protein (MCP) signalling domain	6
378806	Saurantiaca	YP_003955506	HAMP domain	Methyl-accepting chemotaxis protein (MCP) signalling domain	5
1242864	Cfuscus	ZP_21229931	HAMP domain	Methyl-accepting chemotaxis protein (MCP) signalling domain	5
448385	Scellulosum	YP_001611415	HAMP domain	Methyl-accepting chemotaxis protein (MCP) signalling domain	1

			FrzCD domain		
246197	Mxanthus	YP_630952	HAMP domain	Methyl-accepting chemotaxis protein (MCP) signalling domain	1
483219	Mfulvus	YP_004667253	HAMP domain	Methyl-accepting chemotaxis protein (MCP) signalling domain	2
1192034	Capiculatus	ZP_11024060	HAMP domain	Methyl-accepting chemotaxis protein (MCP) signalling domain	2
1278073	Mstipitatus	YP_007360139	HAMP domain	Methyl-accepting chemotaxis protein (MCP) signalling domain	2
1144275	Ccoralloides	YP_005371258	HAMP domain	Methyl-accepting chemotaxis protein (MCP) signalling domain	2

378806	Saurantiaca	YP_003953104	HAMP domain	Methyl-accepting chemotaxis protein (MCP) signalling domain	2
1242864	Cfuscus	ZP_21231947	HAMP domain	Methyl-accepting chemotaxis protein (MCP) signalling domain	3
404380	Gbemidjiensis	YP_002139189	HAMP domain	Methyl-accepting chemotaxis protein (MCP) signalling domain	2
443144	Gsp	YP_003021648	HAMP domain	Methyl-accepting chemotaxis protein (MCP) signalling domain	2
351605	Guraniireducens	YP_001232886	HAMP domain	Methyl-accepting chemotaxis protein (MCP) signalling domain	2

				FrzCD domain			
316067	Gdaltonii	YP_002536023	HAMP domain	Methyl-accepting chemotaxis protein (MCP) signalling domain			2
661367	Llongbeachae	YP_003456745	HAMP domain	Methyl-accepting chemotaxis protein (MCP) signalling domain			2
383372	Rcastenholzii	YP_001433502	HAMP domain	HAMP domain	Methyl-accepting chemotaxis protein (MCP) signalling domain		2
357808	Rsp	YP_001275854	HAMP domain	Methyl-accepting chemotaxis protein (MCP) signalling domain			2
324602	Caurantiacus	YP_001634474	HAMP domain	Methyl-accepting chemotaxis protein (MCP) signalling domain			1

FrzCD domain

706587	Dtiedjei	YP_006445301	HAMP domain	Methyl-accepting chemotaxis protein (MCP) signalling domain	2
706587	Dtiedjei	YP_006449120	HAMP domain	Methyl-accepting chemotaxis protein (MCP) signalling domain	2
526222	Dsalexigens	YP_002992533	HAMP domain	Methyl-accepting chemotaxis protein (MCP) signalling domain	2
439235	Dalkenivorans	YP_002433813	HAMP domain	Methyl-accepting chemotaxis protein (MCP) signalling domain	2
439235	Dalkenivorans	YP_002431563	HAMP domain	Methyl-accepting chemotaxis protein (MCP) signalling domain	2

			FrzCD domain		
439235	Dalkenivorans	YP_002430843	HAMP domain	Methyl-accepting chemotaxis protein (MCP) signalling domain	2
439235	Dalkenivorans	YP_002430739	HAMP domain	Methyl-accepting chemotaxis protein (MCP) signalling domain	2
439235	Dalkenivorans	YP_002430111	HAMP domain	Methyl-accepting chemotaxis protein (MCP) signalling domain	2
439235	Dalkenivorans	YP_002433864	HAMP domain	Methyl-accepting chemotaxis protein (MCP) signalling domain	2
439235	Dalkenivorans	YP_002430249	HAMP domain	Methyl-accepting chemotaxis protein (MCP) signalling domain	2

296591	Psp	YP_549280	HAMP domain	Methyl-accepting chemotaxis protein (MCP) signalling domain	2
748280	Psp	YP_004848389	HAMP domain	Methyl-accepting chemotaxis protein (MCP) signalling domain	2
651182	Dtoluolica	YP_006760765	HAMP domain	Methyl-accepting chemotaxis protein (MCP) signalling domain	2
394	Sfredii	YP_002822595	HAMP domain	Methyl-accepting chemotaxis protein (MCP) signalling domain	2
1185652	Sfredii	YP_006398343	HAMP domain	Methyl-accepting chemotaxis protein (MCP) signalling domain	2

				FrzCD domain	
1117943	Sfredii	YP_005192767	HAMP domain	Methyl-accepting chemotaxis protein (MCP) signalling domain	2
1230587	Smeliloti	YP_006814673	HAMP domain	Methyl-accepting chemotaxis protein (MCP) signalling domain	2
693982	Smeliloti	YP_004551680	HAMP domain	Methyl-accepting chemotaxis protein (MCP) signalling domain	2
698936	Smeliloti	YP_005718244	HAMP domain	Methyl-accepting chemotaxis protein (MCP) signalling domain	2
1286640	Smeliloti	YP_007572808	HAMP domain	Methyl-accepting chemotaxis protein (MCP) signalling domain	2

				FrzCD domain	
707241	Smeliloti	YP_0057 24684	HAMP domain	Methyl-accepting chemotax is protein (MCP) signalling domain	1
1235461	Smeliloti	YP_0071 92846	HAMP domain	Methyl-accepting chemotax is protein (MCP) signalling domain	2
266834	Smeliloti	NP_4360 93	HAMP domain	Methyl-accepting chemotax is protein (MCP) signalling domain	1
366394	Smedicae	YP_0013 13827	HAMP domain	Methyl-accepting chemotax is protein (MCP) signalling domain	2
404589	Asp	YP_0013 79482	HAMP domain	Methyl-accepting chemotax is protein (MCP) signalling domain	2

				FrzCD domain	
447217	Asp	YP_0021 35031	HAMP domain	Methyl-accepting chemotax is protein (MCP) signalling domain	2
455488	Adehalog enans	YP_0024 93175	HAMP domain	Methyl-accepting chemotax is protein (MCP) signalling domain	2
290397	Adehalog enans	YP_4644 05	HAMP domain	Methyl-accepting chemotax is protein (MCP) signalling domain	2
137722	Asp	YP_0034 50388	HAMP domain	Methyl-accepting chemotax is protein (MCP) signalling domain	2
862719	Alipoferu m	YP_0049 74655	HAMP domain	Methyl-accepting chemotax is protein (MCP) signalling domain	2

				FrzCD domain	
634177	Gxylinus	YP_0048 67742	HAMP domain	Methyl-accepting chemotax is protein (MCP) signalling domain	1
272568	Gdiazotro phicus	YP_0016 03521	HAMP domain	Methyl-accepting chemotax is protein (MCP) signalling domain	1
272568	Gdiazotro phicus	YP_0022 77422	HAMP domain	Methyl-accepting chemotax is protein (MCP) signalling domain	1
450851	Pzucineu m	YP_0021 29536	HAMP domain	Methyl-accepting chemotax is protein (MCP) signalling domain	2
426355	Mradiotol erans	YP_0017 54863	HAMP domain	Methyl-accepting chemotax is protein (MCP) signalling domain	2

FrzCD domain

460265	Mnodulans	YP_002500599	HAMP domain	Methyl-accepting chemotaxis protein (MCP) signalling domain	1
426117	Msp	YP_001767248	HAMP domain	Methyl-accepting chemotaxis protein (MCP) signalling domain	1
414684	Rcentenum	YP_002298330	HAMP domain	Methyl-accepting chemotaxis protein (MCP) signalling domain	2
404589	Asp	YP_001380701	HAMP domain	Methyl-accepting chemotaxis protein (MCP) signalling domain	2
404589	Asp	YP_001379585	HAMP domain	Methyl-accepting chemotaxis protein (MCP) signalling domain	2

1278073	Mstipitatus	YP_007360083	HAMP domain	Methyl-accepting chemotaxis protein (MCP) signalling domain	2
1144275	Ccoralloides	YP_005371314	HAMP domain	Methyl-accepting chemotaxis protein (MCP) signalling domain	2
483219	Mfulvus	YP_004667200	HAMP domain	Methyl-accepting chemotaxis protein (MCP) signalling domain	2
1192034	Capiculatus	ZP_11024004	HAMP domain	Methyl-accepting chemotaxis protein (MCP) signalling domain	2
246197	Mxanthus	YP_630900	HAMP domain	Methyl-accepting chemotaxis protein (MCP) signalling domain	2

378806	Saurantiaca	YP_003953037	HAMP domain	Methyl-accepting chemotaxis protein (MCP) signalling domain	2
1242864	Cfuscus	ZP_21235648	HAMP domain	Methyl-accepting chemotaxis protein (MCP) signalling domain	2
1278073	Mstipitatus	YP_007357212	HAMP domain	Methyl-accepting chemotaxis protein (MCP) signalling domain	2
1192034	Capiculatus	ZP_11026759	HAMP domain	Methyl-accepting chemotaxis protein (MCP) signalling domain	2
246197	Mxanthus	YP_628457	HAMP domain	Methyl-accepting chemotaxis protein (MCP) signalling domain	2

				FrzCD domain	
483219	Mfulvus	YP_004664567	HAMP domain	Methyl-accepting chemotaxis protein (MCP) signalling domain	2
1144275	Ccoralloides	YP_005373808	HAMP domain	Methyl-accepting chemotaxis protein (MCP) signalling domain	2
378806	Saurantiaca	YP_003951496	HAMP domain	Methyl-accepting chemotaxis protein (MCP) signalling domain	6
1278073	Mstipitatus	YP_007361249	HAMP domain	Methyl-accepting chemotaxis protein (MCP) signalling domain	3
246197	Mxanthus	YP_631855	HAMP domain	Methyl-accepting chemotaxis protein (MCP) signalling domain	2

				FrzCD domain	
483219	Mfulvus	YP_004668261	HAMP domain	Methyl-accepting chemotaxis protein (MCP) signalling domain	1
1242864	Cfuscus	ZP_21231501	HAMP domain	Methyl-accepting chemotaxis protein (MCP) signalling domain	2
378806	Saurantiaca	YP_003956288	HAMP domain	Methyl-accepting chemotaxis protein (MCP) signalling domain	2
1144275	Ccoralloides	YP_005372514	HAMP domain	Methyl-accepting chemotaxis protein (MCP) signalling domain	2
483219	Mfulvus	YP_004666214	HAMP domain	Methyl-accepting chemotaxis protein (MCP) signalling domain	2

				FrzCD domain	
246197	Mxanthus	YP_634162	HAMP domain	Methyl-accepting chemotaxis protein (MCP) signalling domain	2
1192034	Capiculatus	ZP_11024978	HAMP domain	Methyl-accepting chemotaxis protein (MCP) signalling domain	2
1278073	Mstipitatus	YP_007363611	HAMP domain	Methyl-accepting chemotaxis protein (MCP) signalling domain	2
1278073	Mstipitatus	YP_007361071	HAMP domain	Methyl-accepting chemotaxis protein (MCP) signalling domain	2
1192034	Capiculatus	ZP_11025458	HAMP domain	Methyl-accepting chemotaxis protein (MCP) signalling domain	2

			FrzCD domain		
483219	Mfulvus	YP_0046 66749	HAMP domain	Methyl-accepting chemotax is protein (MCP) signalling domain	1
1144275	Ccoralloid es	YP_0053 72983	HAMP domain	Methyl-accepting chemotax is protein (MCP) signalling domain	2
1242864	Cfuscus	ZP_2123 6587	HAMP domain	Methyl-accepting chemotax is protein (MCP) signalling domain	2
246197	Mxanthus	YP_6291 39	HAMP domain	Methyl-accepting chemotax is protein (MCP) signalling domain	2
1192034	Capiculat us	ZP_1102 4751	HAMP domain	Methyl-accepting chemotax is protein (MCP) signalling domain	2

				FrzCD domain	
483219	Mfulvus	YP_004663866	HAMP domain	Methyl-accepting chemotaxis protein (MCP) signalling domain	2
1144275	Ccoralloides	YP_005366771	HAMP domain	Methyl-accepting chemotaxis protein (MCP) signalling domain	2
378806	Saurantiaca	YP_003956926	HAMP domain	Methyl-accepting chemotaxis protein (MCP) signalling domain	2
378806	Saurantiaca	YP_003951826	HAMP domain	Methyl-accepting chemotaxis protein (MCP) signalling domain	2
502025	Hochraceum	YP_003271143	Methyl-accepting chemotaxis protein (MCP) signalling domain		2

404589	Asp	YP_001377853	Methyl-accepting chemotaxis protein (MCP) signalling domain		0
290397	Adehalogenans	YP_463825	Methyl-accepting chemotaxis protein (MCP) signalling domain		0
447217	Asp	YP_002133014	Methyl-accepting chemotaxis protein (MCP) signalling domain		0
455488	Adehalogenans	YP_002491060	Methyl-accepting chemotaxis protein (MCP) signalling domain		0
378806	Saurantiaca	YP_003954195	Methyl-accepting chemotaxis protein (MCP) signalling domain		0

FrzCD domain

246197	Mxanthus	YP_632316	Methyl-accepting chemotaxis protein (MCP) signalling domain			0
483219	Mfulvus	YP_004668731	Methyl-accepting chemotaxis protein (MCP) signalling domain			0
1192034	Capiculatus	ZP_11025709	Methyl-accepting chemotaxis protein (MCP) signalling domain			0
1144275	Ccoralloides	YP_005369807	Methyl-accepting chemotaxis protein (MCP) signalling domain			0
1242864	Cfuscus	ZP_21239145	Methyl-accepting chemotaxis protein (MCP) signalling domain			0

1278073	Mstipitatus	YP_007360801	Methyl-accepting chemotaxis protein (MCP) signalling domain	0
351607	Acellulolyticus	YP_873630	Methyl-accepting chemotaxis protein (MCP) signalling domain	5
649831	Asp	YP_007952951	Methyl-accepting chemotaxis protein (MCP) signalling domain	2
512565	Amissouriensis	YP_005464979	Methyl-accepting chemotaxis protein (MCP) signalling domain	2
134676	Asp	YP_006268094	Methyl-accepting chemotaxis protein (MCP) signalling domain	2

FrzCD domain

404589	Asp	YP_001379554	Methyl-accepting chemotaxis protein (MCP) signalling domain	2
316274	Haurantiacus	YP_001543500	Methyl-accepting chemotaxis protein (MCP) signalling domain	6
326427	Caggregans	YP_002463715	Methyl-accepting chemotaxis protein (MCP) signalling domain	5
480224	Csp	YP_002569541	Methyl-accepting chemotaxis protein (MCP) signalling domain	5
324602	Caurantiacus	YP_001635273	Methyl-accepting chemotaxis protein (MCP) signalling domain	5

383372	Rcastenholzii	YP_001434250	Methyl-accepting chemotaxis protein (MCP) signalling domain	5
357808	Rsp	YP_001274430	Methyl-accepting chemotaxis protein (MCP) signalling domain	6
111780	Scyanosphaera	YP_007131581	Methyl-accepting chemotaxis protein (MCP) signalling domain	2
395962	Csp	YP_003136563	Methyl-accepting chemotaxis protein (MCP) signalling domain	2
41431	Csp	YP_002371001	Methyl-accepting chemotaxis protein (MCP) signalling domain	2

497965	Csp	YP_003889539	Methyl-accepting chemotaxis protein (MCP) signalling domain	2
497965	Csp	YP_003889540	Methyl-accepting chemotaxis protein (MCP) signalling domain	1
1173022	Cepipsammum	YP_007140536	Methyl-accepting chemotaxis protein (MCP) signalling domain	2
56110	Oacuminata	YP_007085836	Methyl-accepting chemotaxis protein (MCP) signalling domain	2
748280	Psp	YP_004847108	Methyl-accepting chemotaxis protein (MCP) signalling domain	8

			FrzCD domain	
443143	Gsp	YP_004200706	Methyl-accepting chemotaxis protein (MCP) signalling domain	8
1144275	Ccoralloides	YP_005373463	Methyl-accepting chemotaxis protein (MCP) signalling domain	5
1278073	Mstipitatus	YP_007364504	Methyl-accepting chemotaxis protein (MCP) signalling domain	5
246197	Mxanthus	YP_635052	Methyl-accepting chemotaxis protein (MCP) signalling domain	5
1192034	Capiculatus	ZP_11024430	Methyl-accepting chemotaxis protein (MCP) signalling domain	6

FrzCD domain

1144275	Ccoralloides	YP_005367165	Methyl-accepting chemotaxis protein (MCP) signalling domain				6
246197	Mxanthus	YP_629507	Methyl-accepting chemotaxis protein (MCP) signalling domain				5
1242864	Cfuscus	ZP_21230692	Methyl-accepting chemotaxis protein (MCP) signalling domain				6
443143	Gsp	YP_004198944	6xPAS domain	Methyl-accepting chemotaxis protein (MCP) signalling domain			0
349521	Hchejuensis	YP_432606	PAS domain	PAS domain	Methyl-accepting chemotaxis protein (MCP) signalling domain		0

			FrzCD domain		
156889	Mmarinus	YP_865747	Protoglobin	Methyl-accepting chemotaxis protein (MCP) signalling domain	0
1242864	Cfuscus	ZP_21237045	Protoglobin	Methyl-accepting chemotaxis protein (MCP) signalling domain	0
56110	Oacuminata	YP_007084155	Type IV pili methyl-accepting chemotaxis transducer N-term	Methyl-accepting chemotaxis protein (MCP) signalling domain	1
111780	Scyanosphaera	YP_007131582	Type IV pili methyl-accepting chemotaxis transducer N-term	Methyl-accepting chemotaxis protein (MCP) signalling domain	2
1173027	Msp	YP_007123429	Type IV pili methyl-accepting chemotaxis transducer N-term	Methyl-accepting chemotaxis protein (MCP) signalling domain	2

			FrzCD domain			
342108	Mmagneticum	YP_421328	Type IV pili methyl-accepting chemotaxis transducer N-term	HAMP domain	Methyl-accepting chemotaxis protein (MCP) signalling domain	2

FrzE domain

GI	Organis	Refseq	pfam					
765952	Pacantha moebae	YP_0046 52530	Hpt domain	Signal transduci ng histidine kinase, homodim eric [illegible]	Histidine kinase-, DNA gyrase B-, and HSP90- like [illegible]	CheW-like domain	Response regulator receiver domain	
498761	Hmodesti caldum	YP_0016 80594	Hpt domain	Signal transduci ng histidine kinase, homodim eric [illegible]	Histidine kinase-, DNA gyrase B-, and HSP90- like [illegible]	CheW-like domain	Response regulator receiver domain	
1173025	Gsp	YP_0071 11208	Hpt domain	Signal transduci ng histidine kinase, homodim eric [illegible]	Histidine kinase-, DNA gyrase B-, and HSP90- like [illegible]	CheW-like domain	Response regulator receiver domain	
1173027	Msp	YP_0071 23430	3xHpt domain	Signal transduci ng histidine kinase, homodim eric [illegible]	Histidine kinase-, DNA gyrase B-, and HSP90- like [illegible]	CheW-like domain	Response regulator receiver domain	
497965	Csp	YP_0038 89538	Hpt domain	Signal transduci ng histidine kinase, homodim eric [illegible]	Histidine kinase-, DNA gyrase B-, and HSP90- like [illegible]	CheW-like domain	Response regulator receiver domain	
395962	Csp	YP_0031 36564	Hpt domain	Histidine kinase-, DNA gyrase B-, and HSP90- like [illegible]	CheW-like domain	Response regulator receiver domain		

FrzE domain

41431	Csp	YP_002371002	Hpt domain	Histidine kinase-, DNA gyrase B-, and HSP90-like ATPase	CheW-like domain	Response regulator receiver domain	
65093	Hsp	YP_007167429	2xHpt domain	Signal transducing histidine kinase, homodimeric domain	Histidine kinase-, DNA gyrase B-, and HSP90-like ATPase	CheW-like domain	Response regulator receiver domain
179408	Onigro	YP_007114804	Hpt domain	Signal transducing histidine kinase, homodimeric domain	Histidine kinase-, DNA gyrase B-, and HSP90-like ATPase	CheW-like domain	Response regulator receiver domain
56110	Oacuminata	YP_007085911	Hpt domain	Signal transducing histidine kinase, homodimeric domain	Histidine kinase-, DNA gyrase B-, and HSP90-like ATPase	CheW-like domain	Response regulator receiver domain
696747	Aplatensis	YP_005069488	Hpt domain	Signal transducing histidine kinase, homodimeric domain	Histidine kinase-, DNA gyrase B-, and HSP90-like ATPase	CheW-like domain	Response regulator receiver domain
1173022	Cepipsammum	YP_007140535	Hpt domain	Signal transducing histidine kinase, homodimeric domain	Histidine kinase-, DNA gyrase B-, and HSP90-like ATPase	CheW-like domain	Response regulator receiver domain

FrzE domain

1173022	Cepipsammum	YP_007142621	Hpt domain	Signal transducing histidine kinase, homodimeric domain	Histidine kinase-, DNA gyrase B-, and HSP90-like ATPase	CheW-like domain	Response regulator receiver domain
329726	Amarina	YP_001519361	Hpt domain	Signal transducing histidine kinase, homodimeric domain	Histidine kinase-, DNA gyrase B-, and HSP90-like ATPase	CheW-like domain	Response regulator receiver domain
1173027	Msp	YP_007121899	Hpt domain	Signal transducing histidine kinase, homodimeric domain	Histidine kinase-, DNA gyrase B-, and HSP90-like ATPase	CheW-like domain	Response regulator receiver domain
1173263	Ssp	YP_007106310	Hpt domain	Signal transducing histidine kinase, homodimeric domain	Histidine kinase-, DNA gyrase B-, and HSP90-like ATPase	CheW-like domain	Response regulator receiver domain
111780	Scyanosphaera	YP_007131580	Hpt domain	Signal transducing histidine kinase, homodimeric domain	Histidine kinase-, DNA gyrase B-, and HSP90-like ATPase	CheW-like domain	Response regulator receiver domain
1173020	Cminutus	YP_007098865	Hpt domain	Histidine kinase-, DNA gyrase B-, and HSP90-like ATPase	CheW-like domain	Response regulator receiver domain	

				FrzE domain			
251229	Cthermalis	YP_007089442	Hpt domain	Signal transducing histidine kinase, homodimeric domain	Histidine kinase-, DNA gyrase B-, and HSP90-like ATPase	CheW-like domain	Response regulator receiver domain
443144	Gsp	YP_003022165	Hpt domain	Signal transducing histidine kinase, homodimeric domain	Histidine kinase-, DNA gyrase B-, and HSP90-like ATPase	CheW-like domain	Response regulator receiver domain
443143	Gsp	YP_004196843	Hpt domain	Signal transducing histidine kinase, homodimeric domain	Histidine kinase-, DNA gyrase B-, and HSP90-like ATPase	CheW-like domain	Response regulator receiver domain
632348	Ckronotskyensis	YP_004023135	Hpt domain	Signal transducing histidine kinase, homodimeric domain	Histidine kinase-, DNA gyrase B-, and HSP90-like ATPase	CheW-like domain	
639282	Ddesulfuricans	YP_003497215	Hpt domain	Histidine kinase-, DNA gyrase B-, and HSP90-like ATPase	Response regulator receiver domain		
484019	Tafricanus	YP_002335236	Hpt domain	Histidine kinase-, DNA gyrase B-, and HSP90-like ATPase	Response regulator receiver domain		

FrzE domain

289376	Tyellowstonii	YP_002248238	Hpt domain	Histidine kinase-, DNA gyrase B-, and HSP90-like [illegible]	CheW-like domain	Response regulator receiver domain	
768670	Cnitroreducens	YP_004050730	Hpt domain	Histidine kinase-, DNA gyrase B-, and HSP90-like [illegible]	CheW-like domain	Response regulator receiver domain	
717231	Fsinusarabici	YP_004602373	Hpt domain	Signal transducing histidine kinase, homodimeric [illegible]	Histidine kinase-, DNA gyrase B-, and HSP90-like [illegible]	CheW-like domain	Response regulator receiver domain
639282	Ddesulfuricans	YP_003495432	Hpt domain	Signal transducing histidine kinase, homodimeric [illegible]	Histidine kinase-, DNA gyrase B-, and HSP90-like [illegible]	CheW-like domain	Response regulator receiver domain
545694	Tprimitia	YP_004531341	Hpt domain	Histidine kinase-, DNA gyrase B-, and HSP90-like [illegible]	CheW-like domain	Response regulator receiver domain	
906968	Tbrennaborense	YP_004440046	Hpt domain	Histidine kinase-, DNA gyrase B-, and HSP90-like [illegible]	CheW-like domain	Response regulator receiver domain	

			FrzE domain			
1144275	Ccoralloides	YP_005371311	Hpt domain	Histidine kinase-, DNA gyrase B-, and HSP90-like [illegible]	CheW-like domain	Response regulator receiver domain
1278073	Mstipitatus	YP_007360086	Hpt domain	Histidine kinase-, DNA gyrase B-, and HSP90-like [illegible]	CheW-like domain	Response regulator receiver domain
1192034	Capiculatus	ZP_11024007	Hpt domain	Histidine kinase-, DNA gyrase B-, and HSP90-like [illegible]	CheW-like domain	Response regulator receiver domain
246197	Mxanthus	YP_630903	Hpt domain	Histidine kinase-, DNA gyrase B-, and HSP90-like [illegible]	CheW-like domain	Response regulator receiver domain
483219	Mfulvus	YP_004667203	Hpt domain	Histidine kinase-, DNA gyrase B-, and HSP90-like [illegible]	CheW-like domain	Response regulator receiver domain
378806	Saurantiaca	YP_003953040	Hpt domain	Histidine kinase-, DNA gyrase B-, and HSP90-like [illegible]	CheW-like domain	Response regulator receiver domain

FrzE domain

1242864	Cfuscus	ZP_21235645	Hpt domain	Histidine kinase-, DNA gyrase B-, and HSP90-like [illegible]	CheW-like domain	Response regulator receiver domain
404589	Asp	YP_001377668	Hpt domain	Histidine kinase-, DNA gyrase B-, and HSP90-like [illegible]	CheW-like domain	Response regulator receiver domain
404589	Asp	YP_001380698	Hpt domain	Histidine kinase-, DNA gyrase B-, and HSP90-like [illegible]	CheW-like domain	Response regulator receiver domain
1242864	Cfuscus	ZP_21233566	Hpt domain	Histidine kinase-, DNA gyrase B-, and HSP90-like [illegible]	CheW-like domain	Response regulator receiver domain
378806	Saurantiaca	YP_003950120	Hpt domain	Histidine kinase-, DNA gyrase B-, and HSP90-like [illegible]	CheW-like domain	Response regulator receiver domain
404589	Asp	YP_001379553	Hpt domain	Histidine kinase-, DNA gyrase B-, and HSP90-like [illegible]	CheW-like domain	Response regulator receiver domain

				FrzE domain		
512565	Amissouriensis	YP_005464980	Hpt domain	Histidine kinase-, DNA gyrase B-, and HSP90-like [illegible]	CheW-like domain	Response regulator receiver domain
134676	Asp	YP_006268095	Hpt domain	Histidine kinase-, DNA gyrase B-, and HSP90-like [illegible]	CheW-like domain	Response regulator receiver domain
414684	Rcentenum	YP_002298328	Hpt domain	Histidine kinase-, DNA gyrase B-, and HSP90-like [illegible]	CheW-like domain	Response regulator receiver domain
862719	Alipoferum	YP_004974653	Hpt domain	Histidine kinase-, DNA gyrase B-, and HSP90-like [illegible]	CheW-like domain	Response regulator receiver domain
137722	Asp	YP_003450387	Hpt domain	Histidine kinase-, DNA gyrase B-, and HSP90-like [illegible]	CheW-like domain	Response regulator receiver domain
450851	Pzucineum	YP_002129537	Hpt domain	Histidine kinase-, DNA gyrase B-, and HSP90-like [illegible]	CheW-like domain	Response regulator receiver domain

				FrzE domain		
426117	Msp	YP_001767249	Hpt domain	Histidine kinase-, DNA gyrase B-, and HSP90-like [illegible]	CheW-like domain	Response regulator receiver domain
460265	Mnodulans	YP_002500600	Hpt domain	Histidine kinase-, DNA gyrase B-, and HSP90-like [illegible]	CheW-like domain	Response regulator receiver domain
426355	Mradiotolerans	YP_001754864	Hpt domain	Histidine kinase-, DNA gyrase B-, and HSP90-like [illegible]	CheW-like domain	Response regulator receiver domain
441620	Mpopuli	YP_001925647	Hpt domain	Histidine kinase-, DNA gyrase B-, and HSP90-like [illegible]	CheW-like domain	Response regulator receiver domain
419610	Mextorquens	YP_001640300	Hpt domain	Histidine kinase-, DNA gyrase B-, and HSP90-like [illegible]	CheW-like domain	Response regulator receiver domain
272630	Mextorquens	YP_002964066	Hpt domain	Histidine kinase-, DNA gyrase B-, and HSP90-like [illegible]	CheW-like domain	Response regulator receiver domain

661410	Mextorquens	YP_003069099	Hpt domain	Histidine kinase-, DNA gyrase B-, and HSP90-like [illegible]	CheW-like domain	Response regulator receiver domain
440085	Mextorquens	YP_002421831	Hpt domain	Histidine kinase-, DNA gyrase B-, and HSP90-like [illegible]	CheW-like domain	Response regulator receiver domain
404589	Asp	YP_001379483	Hpt domain	Histidine kinase-, DNA gyrase B-, and HSP90-like [illegible]	CheW-like domain	Response regulator receiver domain
447217	Asp	YP_002135032	Hpt domain	Histidine kinase-, DNA gyrase B-, and HSP90-like [illegible]	CheW-like domain	Response regulator receiver domain
455488	Adehalogenans	YP_002493176	Hpt domain	Histidine kinase-, DNA gyrase B-, and HSP90-like [illegible]	CheW-like domain	Response regulator receiver domain
290397	Adehalogenans	YP_464404	Hpt domain	Histidine kinase-, DNA gyrase B-, and HSP90-like [illegible]	CheW-like domain	Response regulator receiver domain

FrzE domain

378806	Saurantiaca	YP_003950502	Hpt domain	Histidine kinase-, DNA gyrase B-, and HSP90-like [illegible]	CheW-like domain	Response regulator receiver domain	
1242864	Cfuscus	ZP_21236412	Hpt domain	Histidine kinase-, DNA gyrase B-, and HSP90-like [illegible]	CheW-like domain	Response regulator receiver domain	
1144275	Ccoralloides	YP_005373472	Hpt domain	Histidine kinase-, DNA gyrase B-, and HSP90-like [illegible]	CheW-like domain	Response regulator receiver domain	
1278073	Mstipitatus	YP_007364519	Hpt domain	Signal transducing histidine kinase, homodimeric [illegible]	Histidine kinase-, DNA gyrase B-, and HSP90-like [illegible]	CheW-like domain	Response regulator receiver domain
1192034	Capiculatus	ZP_11022998	Hpt domain	Histidine kinase-, DNA gyrase B-, and HSP90-like [illegible]	CheW-like domain	Response regulator receiver domain	
246197	Mxanthus	YP_635066	Hpt domain	Histidine kinase-, DNA gyrase B-, and HSP90-like [illegible]	CheW-like domain	Response regulator receiver domain	

FrzE domain

483219	Mfulvus	YP_004665317	Hpt domain	Histidine kinase-, DNA gyrase B-, and HSP90-like ATPase	CheW-like domain	Response regulator receiver domain
266779	Csp	YP_673165	Hpt domain	Histidine kinase-, DNA gyrase B-, and HSP90-like ATPase	CheW-like domain	Response regulator receiver domain
394	Sfredii	YP_002822594	Hpt domain	Histidine kinase-, DNA gyrase B-, and HSP90-like ATPase	CheW-like domain	Response regulator receiver domain
1185652	Sfredii	YP_006398344	Hpt domain	Histidine kinase-, DNA gyrase B-, and HSP90-like ATPase	CheW-like domain	Response regulator receiver domain
1117943	Sfredii	YP_005192766	Hpt domain	Histidine kinase-, DNA gyrase B-, and HSP90-like ATPase	CheW-like domain	Response regulator receiver domain
366394	Smedicae	YP_001313828	Hpt domain	Histidine kinase-, DNA gyrase B-, and HSP90-like ATPase	CheW-like domain	Response regulator receiver domain

FrzE domain

1235461	Smeliloti	YP_007192845	Hpt domain	Histidine kinase-, DNA gyrase B-, and HSP90-like ATPase	CheW-like domain	Response regulator receiver domain
693982	Smeliloti	YP_004551681	Hpt domain	Histidine kinase-, DNA gyrase B-, and HSP90-like ATPase	CheW-like domain	Response regulator receiver domain
707241	Smeliloti	YP_005724683	Hpt domain	Histidine kinase-, DNA gyrase B-, and HSP90-like ATPase	CheW-like domain	Response regulator receiver domain
1230587	Smeliloti	YP_006814674	Hpt domain	Histidine kinase-, DNA gyrase B-, and HSP90-like ATPase	CheW-like domain	Response regulator receiver domain
1286640	Smeliloti	YP_007572809	Hpt domain	Histidine kinase-, DNA gyrase B-, and HSP90-like ATPase	CheW-like domain	Response regulator receiver domain
266834	Smeliloti	NP_436094	Hpt domain	Histidine kinase-, DNA gyrase B-, and HSP90-like ATPase	CheW-like domain	Response regulator receiver domain

FrzE domain

698936	Smeliloti	YP_005718245	Hpt domain	Histidine kinase-, DNA gyrase B-, and HSP90-like ATPase	CheW-like domain	Response regulator receiver domain	
379066	Gaurantiaca	YP_002760850	Hpt domain	Histidine kinase-, DNA gyrase B-, and HSP90-like ATPase	CheW-like domain	Response regulator receiver domain	
480224	Csp	YP_002568674	Hpt domain	Signal transducing histidine kinase, homodimeric domain	Histidine kinase-, DNA gyrase B-, and HSP90-like ATPase	CheW-like domain	Response regulator receiver domain
324602	Caurantiacus	YP_001634473	Hpt domain	Signal transducing histidine kinase, homodimeric domain	Histidine kinase-, DNA gyrase B-, and HSP90-like ATPase	CheW-like domain	Response regulator receiver domain
357808	Rsp	YP_001275357	Hpt domain	Histidine kinase-, DNA gyrase B-, and HSP90-like ATPase	CheW-like domain	Response regulator receiver domain	
383372	Rcastenholzii	YP_001433272	Hpt domain	Histidine kinase-, DNA gyrase B-, and HSP90-like ATPase	CheW-like domain	Response regulator receiver domain	

FrzE domain

1242864	Cfuscus	ZP_21237044	Hpt domain	Histidine kinase-, DNA gyrase B-, and HSP90-like ATPase	CheW-like domain	Response regulator receiver domain
639283	Snovella	YP_003693908	Hpt domain	Histidine kinase-, DNA gyrase B-, and HSP90-like ATPase	CheW-like domain	Response regulator receiver domain
690850	Dafricanus	YP_005051439	Hpt domain	Histidine kinase-, DNA gyrase B-, and HSP90-like ATPase	CheW-like domain	Response regulator receiver domain
1121451	Dhydrothermalis	YP_007327257	Hpt domain	Histidine kinase-, DNA gyrase B-, and HSP90-like ATPase	CheW-like domain	Response regulator receiver domain
526222	Dsalexigens	YP_002990891	Hpt domain	Histidine kinase-, DNA gyrase B-, and HSP90-like ATPase	CheW-like domain	Response regulator receiver domain
443143	Gsp	YP_004198914	Hpt domain	Histidine kinase-, DNA gyrase B-, and HSP90-like ATPase	CheW-like domain	Response regulator receiver domain

404380	Gbemidjiensis	YP_002138836	Hpt domain	Histidine kinase-, DNA gyrase B-, and HSP90-like [illegible]	CheW-like domain	Response regulator receiver domain
443144	Gsp	YP_003022002	Hpt domain	Histidine kinase-, DNA gyrase B-, and HSP90-like [illegible]	CheW-like domain	Response regulator receiver domain
349521	Hchejuensis	YP_435000	Hpt domain	Histidine kinase-, DNA gyrase B-, and HSP90-like [illegible]	CheW-like domain	Response regulator receiver domain
748280	Psp	YP_004848388	Hpt domain	Histidine kinase-, DNA gyrase B-, and HSP90-like [illegible]	CheW-like domain	Response regulator receiver domain
323259	Mhungatei	YP_502458	Hpt domain	Histidine kinase-, DNA gyrase B-, and HSP90-like [illegible]	CheW-like domain	Response regulator receiver domain
323259	Mhungatei	YP_501975	Hpt domain	Histidine kinase-, DNA gyrase B-, and HSP90-like [illegible]	CheW-like domain	Response regulator receiver domain

FrzE domain

368407	Mmarisnigri	YP_001046154	Hpt domain	Histidine kinase-, DNA gyrase B-, and HSP90-like ATPase	CheW-like domain	Response regulator receiver domain
593750	Mformicicum	YP_007249413	Hpt domain	Histidine kinase-, DNA gyrase B-, and HSP90-like ATPase	CheW-like domain	Response regulator receiver domain
521011	Mpalustris	YP_002466385	Hpt domain	Histidine kinase-, DNA gyrase B-, and HSP90-like ATPase	CheW-like domain	Response regulator receiver domain
269799	Gmetallireducen	YP_006721665	Hpt domain	Histidine kinase-, DNA gyrase B-, and HSP90-like ATPase	CheW-like domain	Response regulator receiver domain
443143	Gsp	YP_004198943	Hpt domain	Histidine kinase-, DNA gyrase B-, and HSP90-like ATPase	CheW-like domain	Response regulator receiver domain
443143	Gsp	YP_004200705	Hpt domain	Histidine kinase-, DNA gyrase B-, and HSP90-like ATPase	CheW-like domain	Response regulator receiver domain

FrzE domain

706587	Dtiedjei	YP_006445054	Hpt domain	Signal transducing histidine kinase, homodimeric domain	Histidine kinase-, DNA gyrase B-, and HSP90-like ATPase	CheW-like domain	Response regulator receiver domain
651182	Dtoluolica	YP_006760764	Hpt domain	Histidine kinase-, DNA gyrase B-, and HSP90-like ATPase	CheW-like domain	Response regulator receiver domain	
296591	Psp	YP_549283	Hpt domain	Histidine kinase-, DNA gyrase B-, and HSP90-like ATPase	CheW-like domain	Response regulator receiver domain	
439235	Dalkenivorans	YP_002433812	Hpt domain	Histidine kinase-, DNA gyrase B-, and HSP90-like ATPase	CheW-like domain	Response regulator receiver domain	
323848	Nmultiformis	YP_411033	Hpt domain	Histidine kinase-, DNA gyrase B-, and HSP90-like ATPase	CheW-like domain	Response regulator receiver domain	
56780	Saciditrophicus	YP_460739	Hpt domain	Histidine kinase-, DNA gyrase B-, and HSP90-like ATPase	CheW-like domain	Response regulator receiver domain	

FrzE domain

502025	Hochraceum	YP_003267648	Hpt domain	Histidine kinase-, DNA gyrase B-, and HSP90-like [illegible]	CheW-like domain	Response regulator receiver domain	
525903	Tacidaminovoran	YP_003316876	Hpt domain	Histidine kinase-, DNA gyrase B-, and HSP90-like [illegible]	CheW-like domain	Response regulator receiver domain	
156889	Mmarinus	YP_865748	Hpt domain	Histidine kinase-, DNA gyrase B-, and HSP90-like [illegible]	CheW-like domain	Response regulator receiver domain	
156889	Mmarinus	YP_865397	Hpt domain	Signal transducing histidine kinase, homodimeric [illegible]	Histidine kinase-, DNA gyrase B-, and HSP90-like [illegible]	CheW-like domain	Response regulator receiver domain
572477	Avinosum	YP_003442574	Hpt domain	Signal transducing histidine kinase, homodimeric [illegible]	Histidine kinase-, DNA gyrase B-, and HSP90-like [illegible]	CheW-like domain	Response regulator receiver domain
342108	Mmagneticum	YP_422367	Hpt domain	Signal transducing histidine kinase, homodimeric [illegible]	Histidine kinase-, DNA gyrase B-, and HSP90-like [illegible]	CheW-like domain	Response regulator receiver domain

FrzE domain

1150469	Rphotometricum	YP_005416581	Hpt domain	Signal transducing histidine kinase, homodimeric domain	Histidine kinase-, DNA gyrase B-, and HSP90-like ATPase	CheW-like domain	Response regulator receiver domain
404589	Asp	YP_001377854	Hpt domain	Histidine kinase-, DNA gyrase B-, and HSP90-like ATPase	CheW-like domain	Response regulator receiver domain	
290397	Adehalogenans	YP_463826	Hpt domain	Histidine kinase-, DNA gyrase B-, and HSP90-like ATPase	CheW-like domain	Response regulator receiver domain	
447217	Asp	YP_002133015	Hpt domain	Histidine kinase-, DNA gyrase B-, and HSP90-like ATPase	CheW-like domain	Response regulator receiver domain	
455488	Adehalogenans	YP_002491061	Hpt domain	Histidine kinase-, DNA gyrase B-, and HSP90-like ATPase	CheW-like domain	Response regulator receiver domain	
1242864	Cfuscus	ZP_21239144	Hpt domain	Histidine kinase-, DNA gyrase B-, and HSP90-like ATPase	CheW-like domain	Response regulator receiver domain	

FrzE domain

378806	Saurantiaca	YP_003954194	Hpt domain	Histidine kinase-, DNA gyrase B-, and HSP90-like [illegible]	CheW-like domain	Response regulator receiver domain
1144275	Ccoralloides	YP_005369808	Hpt domain	Histidine kinase-, DNA gyrase B-, and HSP90-like [illegible]	CheW-like domain	Response regulator receiver domain
1278073	Mstipitatus	YP_007360802	Hpt domain	Histidine kinase-, DNA gyrase B-, and HSP90-like [illegible]	CheW-like domain	Response regulator receiver domain
1192034	Capiculatus	ZP_11025708	Hpt domain	Histidine kinase-, DNA gyrase B-, and HSP90-like [illegible]	CheW-like domain	Response regulator receiver domain
246197	Mxanthus	YP_632315	Hpt domain	Histidine kinase-, DNA gyrase B-, and HSP90-like [illegible]	CheW-like domain	Response regulator receiver domain
483219	Mfulvus	YP_004668730	Hpt domain	Histidine kinase-, DNA gyrase B-, and HSP90-like [illegible]	CheW-like domain	Response regulator receiver domain

FrzE domain

378806	Saurantiaca	YP_003956290	Hpt domain	Histidine kinase-, DNA gyrase B-, and HSP90-like ATPase	CheW-like domain	Response regulator receiver domain
1242864	Cfuscus	ZP_21231499	Hpt domain	Histidine kinase-, DNA gyrase B-, and HSP90-like ATPase	CheW-like domain	Response regulator receiver domain
1144275	Ccoralloides	YP_005372516	Hpt domain	Histidine kinase-, DNA gyrase B-, and HSP90-like ATPase	CheW-like domain	Response regulator receiver domain
1278073	Mstipitatus	YP_007363613	Hpt domain	Histidine kinase-, DNA gyrase B-, and HSP90-like ATPase	CheW-like domain	Response regulator receiver domain
483219	Mfulvus	YP_004666212	Hpt domain	Histidine kinase-, DNA gyrase B-, and HSP90-like ATPase	CheW-like domain	Response regulator receiver domain
1192034	Capiculatus	ZP_11024980	Histidine kinase-, DNA gyrase B-, and HSP90-like ATPase	CheW-like domain	Response regulator receiver domain	

			FrzE domain			
246197	Mxanthus	YP_634164	Hpt domain	Histidine kinase-, DNA gyrase B-, and HSP90-like [illegible]	CheW-like domain	Response regulator receiver domain
1242864	Cfuscus	ZP_21229929	Hpt domain	Histidine kinase-, DNA gyrase B-, and HSP90-like [illegible]	CheW-like domain	Response regulator receiver domain
378806	Saurantiaca	YP_003955504	Hpt domain	Histidine kinase-, DNA gyrase B-, and HSP90-like [illegible]	CheW-like domain	Response regulator receiver domain
1144275	Ccoralloides	YP_005368338	Hpt domain	Histidine kinase-, DNA gyrase B-, and HSP90-like [illegible]	CheW-like domain	Response regulator receiver domain
1278073	Mstipitatus	YP_007362605	Hpt domain	Histidine kinase-, DNA gyrase B-, and HSP90-like [illegible]	CheW-like domain	Response regulator receiver domain
1192034	Capiculatus	ZP_11023350	Hpt domain	Histidine kinase-, DNA gyrase B-, and HSP90-like [illegible]	CheW-like domain	Response regulator receiver domain

FrzE domain

483219	Mfulvus	YP_004669700	Hpt domain	Histidine kinase-, DNA gyrase B-, and HSP90-like ATPase	CheW-like domain	Response regulator receiver domain		
246197	Mxanthus	YP_633300	Hpt domain	Histidine kinase-, DNA gyrase B-, and HSP90-like ATPase	CheW-like domain	Response regulator receiver domain		
309801	Troseum	YP_002523497	Hpt domain	Signal transducing histidine kinase, homodimeric domain	Histidine kinase-, DNA gyrase B-, and HSP90-like ATPase	CheW-like domain	CheW-like domain	Response regulator receiver domain
886293	Sacidiphila	YP_007203385	Hpt domain	Histidine kinase-, DNA gyrase B-, and HSP90-like ATPase	CheW-like domain	Response regulator receiver domain		
575540	Ipallida	YP_004178972	2XHpt domain	Histidine kinase-, DNA gyrase B-, and HSP90-like ATPase	CheW-like domain	Response regulator receiver domain		
862719	Alipoferum	YP_005039177	Hpt domain	Histidine kinase-, DNA gyrase B-, and HSP90-like ATPase	CheW-like domain			

FrzE domain

FrzE domain

FrzE domain

FrzE domain

FrzE domain

CheW-like domain

GI	Organis	Refseq	pfam	
404589	Asp	YP_001379556	CheR methyltransferase, SAM binding domain	Tetratricopeptide repeat
1242864	Cfuscus	ZP_21233564	CheR methyltransferase, SAM binding domain	Tetratricopeptide repeat
378806	Saurantiaca	YP_003950117	CheR methyltransferase, SAM binding domain	
649831	Asp	YP_007952949	CheR methyltransferase, SAM binding domain	
1150469	Rphotometricum	YP_005416580	CheR methyltransferase, SAM binding domain	
572477	Avinosum	YP_003442575	CheR methyltransferase, SAM binding domain	Tetratricopeptide repeat
498761	Hmodesticaldum	YP_001680591	CheR methyltransferase, SAM binding domain	Tetratricopeptide repeat
111781	Lsp	YP_007070655	CheR methyltransferase, SAM binding domain	

			FrzF domain		
251229	Cthermalis	YP_007089438	CheR methyltransferase, SAM binding domain	Tetratricopeptide repeat	
1173263	Ssp	YP_007106314	CheR methyltransferase, SAM binding domain	Tetratricopeptide repeat	Tetratricopeptide repeat
1173022	Cepipsammum	YP_007140546	CheR methyltransferase, SAM binding domain	Tetratricopeptide repeat	
497965	Csp	YP_003889542	CheR methyltransferase, SAM binding domain	Tetratricopeptide repeat	
1173027	Msp	YP_007123432	CheR methyltransferase, SAM binding domain		
65093	Hsp	YP_007167425	CheR methyltransferase, all-alpha domain	CheR methyltransferase, SAM binding domain	Tetratricopeptide repeat
41431	Csp	YP_002371004	CheR methyltransferase, SAM binding domain	Tetratricopeptide repeat	
395962	Csp	YP_003136566	CheR methyltransferase, SAM binding domain	Tetratricopeptide repeat	

111780	Scyanosphaera	YP_007131578	CheR methyltransferase, SAM binding domain			
443143	Gsp	YP_004196846	CheR methyltransferase, SAM binding domain	Tetratricopeptide repeat		
443144	Gsp	YP_003022168	CheR methyltransferase, SAM binding domain			
56110	Oacuminata	YP_007087660	CheR methyltransferase, all-alpha domain	CheR methyltransferase, SAM binding domain	Tetratricopeptide repeat	Tetratricopeptide repeat
179408	Onigro	YP_007114800	CheR methyltransferase, SAM binding domain	Tetratricopeptide repeat		
696747	Aplatensis	YP_005069492	CheR methyltransferase, SAM binding domain	Tetratricopeptide repeat	Tetratricopeptide repeat	
1173025	Gsp	YP_007111205	CheR methyltransferase, SAM binding domain			
357808	Rsp	YP_001275352	CheR methyltransferase, all-alpha domain	CheR methyltransferase, SAM binding domain	Tetratricopeptide repeat	

FrzF domain

383372	Rcastenholzii	YP_001433267	CheR methyltransferase, SAM binding domain	Tetratricopeptide repeat	Tetratricopeptide repeat
324602	Caurantiacus	YP_001634469	CheR methyltransferase, all-alpha domain	CheR methyltransferase, SAM binding domain	Tetratricopeptide repeat
480224	Csp	YP_002568670	CheR methyltransferase, all-alpha domain	CheR methyltransferase, SAM binding domain	Tetratricopeptide repeat
326427	Caggregans	YP_002463956	CheR methyltransferase, SAM binding domain	Tetratricopeptide repeat	
450851	Pzucineum	YP_002129534	CheR methyltransferase, all-alpha domain	CheR methyltransferase, SAM binding domain	Tetratricopeptide repeat
426355	Mradiotolerans	YP_001754861	CheR methyltransferase, SAM binding domain		
440085	Mextorquens	YP_002421828	CheR methyltransferase, all-alpha domain	CheR methyltransferase, SAM binding domain	
272630	Mextorquens	YP_002964063	CheR methyltransferase, all-alpha domain	CheR methyltransferase, SAM binding domain	

				FrzF domain
661410	Mextorquens	YP_003069096	CheR methyltransferase, all-alpha domain	CheR methyltransferase, SAM binding domain
419610	Mextorquens	YP_001640297	CheR methyltransferase, all-alpha domain	CheR methyltransferase, SAM binding domain
441620	Mpopuli	YP_001925644	CheR methyltransferase, all-alpha domain	CheR methyltransferase, SAM binding domain
272568	Gdiazotrophicus	YP_002277420	CheR methyltransferase, SAM binding domain	
272568	Gdiazotrophicus	YP_001603523	CheR methyltransferase, SAM binding domain	
634177	Gxylinus	YP_004867744	CheR methyltransferase, SAM binding domain	
426117	Msp	YP_001767247	CheR methyltransferase, all-alpha domain	CheR methyltransferase, SAM binding domain
460265	Mnodulans	YP_002500597	CheR methyltransferase, all-alpha domain	CheR methyltransferase, SAM binding domain

				FrzF domain
1064539	Abrasilense	YP_004985229	CheR methyltransferase, SAM binding domain	
137722	Asp	YP_003450390	CheR methyltransferase, SAM binding domain	
862719	Alipoferum	YP_004974657	CheR methyltransferase, SAM binding domain	
342108	Mmagneticum	YP_421326	CheR methyltransferase, SAM binding domain	Tetratricopeptide repeat
414684	Rcentenum	YP_003475888	CheR methyltransferase, all-alpha domain	CheR methyltransferase, SAM binding domain
661367	Llongbeachae	YP_003456743	CheR methyltransferase, SAM binding domain	
404589	Asp	YP_001379480	CheR methyltransferase, SAM binding domain	Tetratricopeptide repeat
447217	Asp	YP_002135029	CheR methyltransferase, SAM binding domain	Tetratricopeptide repeat

				FrzF domain
455488	Adehalogenans	YP_002493173	CheR methyltransferase, SAM binding domain	Tetratricopeptide repeat
378806	Saurantiaca	YP_003950505	CheR methyltransferase, SAM binding domain	
1242864	Cfuscus	ZP_21236415	CheR methyltransferase, SAM binding domain	
1144275	Ccoralloides	YP_005373469	CheR methyltransferase, SAM binding domain	
246197	Mxanthus	YP_635063	CheR methyltransferase, SAM binding domain	
1192034	Capiculatus	ZP_11022995	CheR methyltransferase, SAM binding domain	
483219	Mfulvus	YP_004665320	CheR methyltransferase, SAM binding domain	Tetratricopeptide repeat
266779	Csp	YP_673162	CheR methyltransferase, SAM binding domain	

				FrzF domain
394	Sfredii	YP_002822597	CheR methyltransferase, SAM binding domain	
1117943	Sfredii	YP_005192769	CheR methyltransferase, SAM binding domain	
1185652	Sfredii	YP_006398341	CheR methyltransferase, SAM binding domain	Tetratricopeptide repeat
1230587	Smeliloti	YP_006814671	CheR methyltransferase, SAM binding domain	
693982	Smeliloti	YP_004551678	CheR methyltransferase, SAM binding domain	
707241	Smeliloti	YP_005724686	CheR methyltransferase, SAM binding domain	
1235461	Smeliloti	YP_007192848	CheR methyltransferase, SAM binding domain	
698936	Smeliloti	YP_005718242	CheR methyltransferase, SAM binding domain	

266834	Smeliloti	NP_436091	CheR methyltransferase, SAM binding domain		
1286640	Smeliloti	YP_007572806	CheR methyltransferase, SAM binding domain		
526222	Dsalexigens	YP_002990889	CheR methyltransferase, SAM binding domain	Tetratricopeptide repeat	
439235	Dalkenivorans	YP_002433815	CheR methyltransferase, SAM binding domain	Tetratricopeptide repeat	
690850	Dafricanus	YP_005051436	CheR methyltransferase, SAM binding domain	Tetratricopeptide repeat	
56780	Saciditrophicus	YP_460736	CheR methyltransferase, SAM binding domain	Tetratricopeptide repeat	
443143	Gsp	YP_004200708	CheR methyltransferase, SAM binding domain		
323848	Nmultiformis	YP_411030	CheR methyltransferase, SAM binding domain	Tetratricopeptide repeat	Tetratricopeptide repeat

				FrzF domain	
269799	Gmetallireducen	YP_006721662	CheR methyltransferase, SAM binding domain		
443143	Gsp	YP_004198911	CheR methyltransferase, SAM binding domain	Tetratricopeptide repeat	Tetratricopeptide repeat
443144	Gsp	YP_003021999	CheR methyltransferase, SAM binding domain	Tetratricopeptide repeat	
404380	Gbemidjiensis	YP_002138839	CheR methyltransferase, SAM binding domain	Tetratricopeptide repeat	
443143	Gsp	YP_004198946	CheR methyltransferase, SAM binding domain	Tetratricopeptide repeat	
296591	Psp	YP_549276	CheR methyltransferase, SAM binding domain		
651182	Dtoluolica	YP_006760767	CheR methyltransferase, SAM binding domain	Tetratricopeptide repeat	
521011	Mpalustris	YP_002466382	CheR methyltransferase, SAM binding domain	Tetratricopeptide repeat	

				FrzF domain
323259	Mhungatei	YP_502461	CheR methyltransferase, SAM binding domain	
706587	Dtiedjei	YP_006445052	CheR methyltransferase, SAM binding domain	Tetratricopeptide repeat
316274	Haurantiacus	YP_001543504	CheR methyltransferase, SAM binding domain	Tetratricopeptide repeat
480224	Csp	YP_002569545	CheR methyltransferase, SAM binding domain	Tetratricopeptide repeat
324602	Caurantiacus	YP_001635277	CheR methyltransferase, SAM binding domain	Tetratricopeptide repeat
326427	Caggregans	YP_002463711	CheR methyltransferase, SAM binding domain	Tetratricopeptide repeat
357808	Rsp	YP_001274426	CheR methyltransferase, SAM binding domain	Tetratricopeptide repeat
383372	Rcastenholzii	YP_001434254	CheR methyltransferase, SAM binding domain	Tetratricopeptide repeat

FrzF domain

351605	Guraniireducens	YP_001232884	Response regulator receiver domain	CheR methyltransferase, SAM binding domain	Tetratricopeptide repeat	Tetratricopeptide repeat	
316067	Gdaltonii	YP_002536025	Response regulator receiver domain	CheR methyltransferase, all-alpha domain	CheR methyltransferase, SAM binding domain	Tetratricopeptide repeat	
443143	Gsp	YP_004198529	Response regulator receiver domain	CheR methyltransferase, all-alpha domain	CheR methyltransferase, SAM binding domain	Tetratricopeptide repeat	
404380	Gbemidjiensis	YP_002139187	Response regulator receiver domain	CheR methyltransferase, all-alpha domain	CheR methyltransferase, SAM binding domain	Tetratricopeptide repeat	Tetratricopeptide repeat
443144	Gsp	YP_003021650	Response regulator receiver domain	CheR methyltransferase, SAM binding domain	Tetratricopeptide repeat	Tetratricopeptide repeat	
378806	Saurantiaca	YP_003954192	CheR methyltransferase, all-alpha domain	CheR methyltransferase, SAM binding domain	Tetratricopeptide repeat		
1278073	Mstipitatus	YP_007360804	CheR methyltransferase, all-alpha domain	CheR methyltransferase, SAM binding domain			
1144275	Ccoralloides	YP_005369810	CheR methyltransferase, SAM binding domain	Tetratricopeptide repeat			

FrzF domain

1242864	Cfuscus	ZP_21239142	CheR methyltransferase, all-alpha domain	CheR methyltransferase, SAM binding domain	Tetratricopeptide repeat	Tetratricopeptide repeat
1192034	Capiculatus	ZP_11025706	CheR methyltransferase, all-alpha domain	CheR methyltransferase, SAM binding domain	Tetratricopeptide repeat	
246197	Mxanthus	YP_632313	CheR methyltransferase, all-alpha domain	CheR methyltransferase, SAM binding domain	Tetratricopeptide repeat	Tetratricopeptide repeat
483219	Mfulvus	YP_004668728	CheR methyltransferase, all-alpha domain	CheR methyltransferase, SAM binding domain		
404589	Asp	YP_001377856	CheR methyltransferase, SAM binding domain	Tetratricopeptide repeat	Tetratricopeptide repeat	
455488	Adehalogenans	YP_002491063	CheR methyltransferase, SAM binding domain	Tetratricopeptide repeat		
447217	Asp	YP_002133017	CheR methyltransferase, SAM binding domain	Tetratricopeptide repeat		
290397	Adehalogenans	YP_463828	CheR methyltransferase, SAM binding domain	Tetratricopeptide repeat		

GI	Organis	Refseq	pfam	FrzG domain
351607	Acellulolyt	YP_87198	Response	CheB methylesterase
404589	Asp	YP_00137	Response	CheB methylesterase
455488	Adehaloge	YP_00249	Response	CheB methylesterase
447217	Asp	YP_00213	Response	CheB methylesterase
290397	Adehaloge	YP_46382	Response	CheB methylesterase
1242864	Cfuscus	ZP_21239	CheB methylesterase	
378806	Saurantiac	YP_00395	CheB methylesterase	
1144275	Ccoralloide	YP_00536	CheB methylesterase	
1278073	Mstipitatu	YP_00736	CheB methylesterase	
1192034	Capiculatu	ZP_11025	CheB methylesterase	
246197	Mxanthus	YP_63231	CheB methylesterase	
483219	Mfulvus	YP_00466	CheB methylesterase	
1242864	Cfuscus	ZP_21237	Response	CheB methylesterase
498761	Hmodestic	YP_00168	Response	CheB methylesterase
443143	Gsp	YP_00419	Response	CheB methylesterase
443144	Gsp	YP_00302	Response	CheB methylesterase
1173020	Cminutus	YP_00709	Response	CheB methylesterase

				FrzG domain
1173027	Msp	YP_00712	Response	CheB methylesterase
111780	Scyanosph	YP_00713	Response	CheB methylesterase
251229	Cthermalis	YP_00708	Response	CheB methylesterase
1173022	Cepipsamr	YP_00714	Response	CheB methylesterase
1173025	Gsp	YP_00711	Response	CheB methylesterase
56110	Oacuminat	YP_00708	Response	CheB methylesterase
179408	Onigro	YP_00711	Response	CheB methylesterase
1173027	Msp	YP_00712	Response	CheB methylesterase
65093	Hsp	YP_00716	Response	CheB methylesterase
395962	Csp	YP_00313	Response	CheB methylesterase
41431	Csp	YP_00237	Response	CheB methylesterase
497965	Csp	YP_00388	Response	CheB methylesterase
696747	Aplatensis	YP_00506	Response	CheB methylesterase
526222	Dsalexigen	YP_00299	Response	CheB methylesterase
1121451	Dhydrothe	YP_00732	Response	CheB methylesterase
443143	Gsp	YP_00419	Response	CheB methylesterase
349521	Hchejuensi	YP_43499	Response	CheB methylesterase

				FrzG domain
748280	Psp	YP_00484	Response	CheB methylesterase
651182	Dtoluolica	YP_00676	Response	CheB methylesterase
439235	Dalkenivor	YP_00243	Response	CheB methylesterase
639283	Snovella	YP_00369	Response	CheB methylesterase
296591	Psp	YP_54928	Response	CheB methylesterase
323848	Nmultiforn	YP_41103	Response	CheB methylesterase
269799	Gmetallire	YP_00672	Response	CheB methylesterase
323259	Mhungatei	YP_50245	Response	CheB methylesterase
521011	Mpalustris	YP_00246	Response	CheB methylesterase
443143	Gsp	YP_00420	Response	CheB methylesterase
706587	Dtiedjei	YP_00644	Response	CheB methylesterase
443143	Gsp	YP_00419	Response	CheB methylesterase
443144	Gsp	YP_00302	Response	CheB methylesterase
404380	Gbemidjier	YP_00213	Response	CheB methylesterase
690850	Dafricanus	YP_00505	Response	CheB methylesterase
480224	Csp	YP_00256	Response	CheB methylesterase
324602	Caurantiac	YP_00163	Response	CheB methylesterase

				FrzG domain
383372	Rcastenho	YP_00143	Response	CheB methylesterase
357808	Rsp	YP_00127	Response	CheB methylesterase
661367	Llongbeacl	YP_00345	Response	CheB methylesterase
156889	Mmarinus	YP_86574	Response	CheB methylesterase
404589	Asp	YP_00137	Response	CheB methylesterase
455488	Adehaloge	YP_00249	Response	CheB methylesterase
290397	Adehaloge	YP_46440	Response	CheB methylesterase
447217	Asp	YP_00213	Response	CheB methylesterase
1242864	Cfuscus	ZP_21236	Response	CheB methylesterase
1144275	Ccoralloide	YP_00537	Response	CheB methylesterase
1278073	Mstipitatus	YP_00736	Response	CheB methylesterase
1192034	Capiculatu	ZP_11022	Response	CheB methylesterase
246197	Mxanthus	YP_63506	Response	CheB methylesterase
378806	Saurantiac	YP_00395	Response	CheB methylesterase
379066	Gaurantiaca	YP_002760851	Response regulator receiver domain	Type II/IV secretion system protein
414684	Rcentenun	YP_00229	Response	CheB methylesterase
342108	Mmagnetic	YP_42132	Response	CheB methylesterase

				FrzG domain
272568	Gdiazotrop	YP_00227	Response	CheB methylesterase
272568	Gdiazotrop	YP_00160	Response	CheB methylesterase
634177	Gxylinus	YP_00486	Response	CheB methylesterase
1064539	Abrasilens	YP_00498	Response	CheB methylesterase
862719	Alipoferum	YP_00497	Response	CheB methylesterase
137722	Asp	YP_00345	Response	CheB methylesterase
450851	Pzucineum	YP_00212	Response	CheB methylesterase
426117	Msp	YP_00176	Response	CheB methylesterase
460265	Mnodulans	YP_00250	Response	CheB methylesterase
426355	Mradiotole	YP_00175	Response	CheB methylesterase
661410	Mextorque	YP_00306	Response	CheB methylesterase
272630	Mextorque	YP_00296	Response	CheB methylesterase
419610	Mextorque	YP_00164	Response	CheB methylesterase
440085	Mextorque	YP_00242	Response	CheB methylesterase
441620	Mpopuli	YP_00192	Response	CheB methylesterase
469383	Cwoesei	YP_00339	Response	CheB methylesterase
1048260	Lferriphilu	YP_00676	Response	CheB methylesterase

				FrzG domain
502025	Hochraceu	YP_00326	Response	CheB methylesterase
314225	Elitoralis	YP_45913	Response	CheB methylesterase
317655	Salaskensi	YP_61678	Response	CheB methylesterase
745310	Ssp	YP_00761	Response	CheB methylesterase
392499	Swittichii	YP_00126	Response	CheB methylesterase
452662	Sjaponicun	YP_00354	Response	CheB methylesterase
690566	Schlorophe	YP_00455	Response	CheB methylesterase
627192	Ssp	YP_00483	Response	CheB methylesterase
378806	Saurantiac	YP_00395	Response	CheB methylesterase
1242864	Cfuscus	ZP_21231	Response	CheB methylesterase
378806	Saurantiac	YP_00395	Response	CheB methylesterase
483219	Mfulvus	YP_00466	Response	CheB methylesterase
1192034	Capiculatu	ZP_11024	Response	CheB methylesterase
246197	Mxanthus	YP_63416	Response	CheB methylesterase
1278073	Mstipitatus	YP_00736	Response	CheB methylesterase
1144275	Ccoralloide	YP_00537	Response	CheB methylesterase
404589	Asp	YP_00137	Response	CheB methylesterase

				FrzG domain
404589	Asp	YP_00138	Response	CheB methylesterase
502025	Hochraceu	YP_00327	Response	CheB methylesterase
649831	Asp	YP_00795	Response	CheB methylesterase
134676	Asp	YP_00626	Response	CheB methylesterase
512565	Amissourie	YP_00546	Response	CheB methylesterase
404589	Asp	YP_00137	Response	CheB methylesterase
378806	Saurantiac	YP_00395	Response	CheB methylesterase
1242864	Cfuscus	ZP_21233	Response	CheB methylesterase
1144275	Ccoralloide	YP_00536	Response	CheB methylesterase
378806	Saurantiac	YP_00395	Response	CheB methylesterase
1242864	Cfuscus	ZP_21229	Response	CheB methylesterase
1278073	Mstipitatus	YP_00736	Response	CheB methylesterase
1192034	Capiculatu	ZP_11023	Response	CheB methylesterase
483219	Mfulvus	YP_00466	Response	CheB methylesterase
246197	Mxanthus	YP_63329	Response	CheB methylesterase
316274	Haurantiac	YP_00154	Response	CheB methylesterase
326427	Caggregan	YP_00246	Response	CheB methylesterase

				FrzG domain
480224	Csp	YP_00256	Response	CheB methylesterase
324602	Caurantiac	YP_00163	Response	CheB methylesterase
357808	Rsp	YP_00127	Response	CheB methylesterase
383372	Rcastenho	YP_00143	Response	CheB methylesterase
572477	Avinosum	YP_00344	Response	CheB methylesterase
342108	Mmagnetic	YP_42236	Response	CheB methylesterase
1150469	Rphotome	YP_00541	Response	CheB methylesterase
156889	Mmarinus	YP_86539	Response	CheB methylesterase
316067	Gdaltonii	YP_00253	Response	CheB methylesterase
351605	Guraniired	YP_00123	Response	CheB methylesterase
443143	Gsp	YP_00419	Response	CheB methylesterase
443144	Gsp	YP_00302	Response	CheB methylesterase
404380	Gbemidjier	YP_00213	Response	CheB methylesterase

GI	Organis	Refseq	lengh	InterPro		
				**Response**	**Coiled-coil**	
455488	Adehaloge	YP_0024ɛ	858	2-121	255-859	
447217	Asp	YP_00213	860	2-121	257-861	
404589	Asp	YP_00137	851	2-121	276-852	
404589	Asp	YP_00137	1370	2-131	282-1320	
447217	Asp	YP_00213	1359	3-133	281-1326	
290397	Adehaloge	YP_46591	1363	3-133	286-1331	
455488	Adehaloge	YP_0024ɛ	1359	3-133	288-1326	
246197	Mxanthus	YP_6312C	1395	2-118	216-1292	**AglZ**
1192034	Capiculatu	ZP_11028	1521	2-120	213-1422	
483219	Mfulvus	YP_0046ɛ	1629	2-101	375-1529	
1278073	Mstipitatu:	YP_0073ɛ	1451	2-117	362-1349	
1144275	Ccoralloide	YP_00537	1531	2-144	359-1440	
378806	Saurantiac	YP_00395	1443	2-122	221-1335	
391625	Ppacifica	ZP_01907	737	7-96	332-715	
1144275	Ccoralloide	YP_0053ɛ	373	3-124	?	
1278073	Mstipitatu:	YP_0073ɛ	592	2-121	300-549	
378806	Saurantiac	YP_00395	593	3-127	286-560	
483219	Mfulvus	YP_0046ɛ	563	2-123	284-519	
1192034	Capiculatu	ZP_11025	577	3-124	283-532	
246197	Mxanthus	YP_63232	562	2-122	283-518	**FrzS**
1242864	Cfuscus	ZP_21234	560	2-121	293-517	
447217	Asp	YP_00213	527	3-126	308-483	
455488	Adehaloge	YP_0024ɛ	527	3-126	308-406	
290397	Adehaloge	YP_4655ɛ	528	3-124	309-484	
404589	Asp	YP_00137	499	3-126	311-448	
448385	Scellulosur	YP_00161	1099	12-133	390-1097	
