# Supplementary material for: Evolution and Design Governing Signal Precision and Amplification in a Bacterial Chemosensory Pathway
Source: PLoS Genet. 2015 Aug 20;11(8):e1005460. doi: 10.1371/journal.pgen.1005460 (PMC4546325; doi:10.1371/journal.pgen.1005460)
Supplement: S5 Table — (DOCX) [file pgen.1005460.s017.docx]

**Table S5: Genomes used to construct the tree of the Deltaproteobacteria**

| Table S5. Genomes from Deltaproteobacteria |  |  |  |  |
| --- | --- | --- | --- | --- |
| Oraganism | .faa genome | .faa plasmid1 | .faa plasmid2 | .faa plasmid3 |
| *Anaeromyxobacter dehalogenans* 2CP-1 | [NC_011891.faa](ftp://ftp.ncbi.nlm.nih.gov/genomes/Bacteria/Anaeromyxobacter_dehalogenans_2CP_1_uid58989/NC_011891.faa) |  |  |  |
| *Anaeromyxobacter dehalogenans* 2CP-C | [NC_007760.faa](ftp://ftp.ncbi.nlm.nih.gov/genomes/Bacteria/Anaeromyxobacter_dehalogenans_2CP_C_uid58135/NC_007760.faa) |  |  |  |
| *Anaeromyxobacter* sp. Fw109-5 | [NC_009675.faa](ftp://ftp.ncbi.nlm.nih.gov/genomes/Bacteria/Anaeromyxobacter_Fw109_5_uid58755/NC_009675.faa) |  |  |  |
| *Anaeromyxobacter* sp. K | [NC_011145.faa](ftp://ftp.ncbi.nlm.nih.gov/genomes/Bacteria/Anaeromyxobacter_K_uid58953/NC_011145.faa) |  |  |  |
| *Bacteriovorax marinus* SJ | [NC_016620.faa](ftp://ftp.ncbi.nlm.nih.gov/genomes/Bacteria/Bacteriovorax_marinus_SJ_uid82341/NC_016620.faa) | [NC_019100.faa](ftp://ftp.ncbi.nlm.nih.gov/genomes/Bacteria/Bacteriovorax_marinus_SJ_uid82341/NC_019100.faa) |  |  |
| *Bdellovibrio bacteriovorus* HD100 | [NC_005363.faa](ftp://ftp.ncbi.nlm.nih.gov/genomes/Bacteria/Bdellovibrio_bacteriovorus_HD100_uid61595/NC_005363.faa) |  |  |  |
| *Bdellovibrio bacteriovorus* str. Tiberius | [NC_019567.faa](ftp://ftp.ncbi.nlm.nih.gov/genomes/Bacteria/Bdellovibrio_bacteriovorus_Tiberius_uid182482/NC_019567.faa) |  |  |  |
| *Bdellovibrio exovorus* JSS | [NC_020813.faa](ftp://ftp.ncbi.nlm.nih.gov/genomes/Bacteria/Bdellovibrio_exovorus_JSS_uid194119/NC_020813.faa) |  |  |  |
| *Bilophila* sp. 4_1_30 | [NZ_ADCO00000000.scaffold.faa.tgz](ftp://ftp.ncbi.nlm.nih.gov/genomes/Bacteria_DRAFT/Bilophila_4_1_30_uid72973/NZ_ADCO00000000.scaffold.faa.tgz) |  |  |  |
| *Bilophila wadsworthia* 3_1_6 | [NZ_ADCP00000000.scaffold.faa.tgz](ftp://ftp.ncbi.nlm.nih.gov/genomes/Bacteria_DRAFT/Bilophila_wadsworthia_3_1_6_uid61875/NZ_ADCP00000000.scaffold.faa.tgz) |  |  |  |
| *Chondromyces apiculatus* DSM 436 | [NZ_AKXT00000000.scaffold.faa.tgz](ftp://ftp.ncbi.nlm.nih.gov/genomes/Bacteria_DRAFT/Chondromyces_apiculatus_DSM_436_uid175317/NZ_AKXT00000000.scaffold.faa.tgz) |  |  |  |
| *Corallococcus coralloides* DSM 2259 | [NC_017030.faa](ftp://ftp.ncbi.nlm.nih.gov/genomes/Bacteria/Corallococcus_coralloides_DSM_2259_uid157997/NC_017030.faa) |  |  |  |
| *Cystobacter fuscus* DSM 2262 | [NZ_ANAH00000000.scaffold.faa.tgz](ftp://ftp.ncbi.nlm.nih.gov/genomes/Bacteria_DRAFT/Cystobacter_fuscus_DSM_2262_uid188352/NZ_ANAH00000000.scaffold.faa.tgz) |  |  |  |
| *delta proteobacterium* MLMS-1 | [NZ_AAQF00000000.scaffold.faa.tgz](ftp://ftp.ncbi.nlm.nih.gov/genomes/Bacteria_DRAFT/delta_proteobacterium_MLMS_1_uid54339/NZ_AAQF00000000.scaffold.faa.tgz) |  |  |  |
| *delta proteobacterium* NaphS2 | [NZ_ADZZ00000000.scaffold.faa.tgz](ftp://ftp.ncbi.nlm.nih.gov/genomes/Bacteria_DRAFT/delta_proteobacterium_NaphS2_uid50675/NZ_ADZZ00000000.scaffold.faa.tgz) |  |  |  |
| *Desulfarculus baarsii* DSM 2075 | [NC_014365.faa](ftp://ftp.ncbi.nlm.nih.gov/genomes/Bacteria/Desulfarculus_baarsii_DSM_2075_uid51371/NC_014365.faa) |  |  |  |
| *Desulfatibacillum alkenivorans* AK-01 | [NC_011768.faa](ftp://ftp.ncbi.nlm.nih.gov/genomes/Bacteria/Desulfatibacillum_alkenivorans_AK_01_uid58913/NC_011768.faa) |  |  |  |
| *Desulfobacca acetoxidans* DSM 11109 | [NC_015388.faa](ftp://ftp.ncbi.nlm.nih.gov/genomes/Bacteria/Desulfobacca_acetoxidans_DSM_11109_uid65785/NC_015388.faa) |  |  |  |
| *Desulfobacter postgatei* 2ac9 | [NZ_AGJR00000000.scaffold.faa.tgz](ftp://ftp.ncbi.nlm.nih.gov/genomes/Bacteria_DRAFT/Desulfobacter_postgatei_2ac9_uid76943/NZ_AGJR00000000.scaffold.faa.tgz) |  |  |  |
| *Desulfobacula toluolica* Tol2 | [NC_018645.faa](ftp://ftp.ncbi.nlm.nih.gov/genomes/Bacteria/Desulfobacula_toluolica_Tol2_uid175777/NC_018645.faa) |  |  |  |
| *Desulfobulbus propionicus* DSM 2032 | [NC_014972.faa](ftp://ftp.ncbi.nlm.nih.gov/genomes/Bacteria/Desulfobulbus_propionicus_DSM_2032_uid62265/NC_014972.faa) |  |  |  |
| *Desulfocapsa sulfexigens* DSM 10523 | [NC_020304.faa](ftp://ftp.ncbi.nlm.nih.gov/genomes/Bacteria/Desulfocapsa_sulfexigens_DSM_10523_uid189952/NC_020304.faa) | [NC_020305.faa](ftp://ftp.ncbi.nlm.nih.gov/genomes/Bacteria/Desulfocapsa_sulfexigens_DSM_10523_uid189952/NC_020305.faa) |  |  |
| *Desulfococcus oleovorans* Hxd3 | [NC_009943.faa](ftp://ftp.ncbi.nlm.nih.gov/genomes/Bacteria/Desulfococcus_oleovorans_Hxd3_uid58777/NC_009943.faa) |  |  |  |
| *Desulfohalobium retbaense* DSM 5692 | [NC_013223.faa](ftp://ftp.ncbi.nlm.nih.gov/genomes/Bacteria/Desulfohalobium_retbaense_DSM_5692_uid59183/NC_013223.faa) | [NC_013224.faa](ftp://ftp.ncbi.nlm.nih.gov/genomes/Bacteria/Desulfohalobium_retbaense_DSM_5692_uid59183/NC_013224.faa) |  |  |
| *Desulfomicrobium baculatum* DSM 4028 | [NC_013173.faa](ftp://ftp.ncbi.nlm.nih.gov/genomes/Bacteria/Desulfomicrobium_baculatum_DSM_4028_uid59217/NC_013173.faa) |  |  |  |
| *Desulfomonile tiedjei* DSM 6799 | [NC_018025.faa](ftp://ftp.ncbi.nlm.nih.gov/genomes/Bacteria/Desulfomonile_tiedjei_DSM_6799_uid168320/NC_018025.faa) | [NC_018026.faa](ftp://ftp.ncbi.nlm.nih.gov/genomes/Bacteria/Desulfomonile_tiedjei_DSM_6799_uid168320/NC_018026.faa) |  |  |
| *Desulfonatronospira thiodismutans* ASO3-1 | [NZ_ACJN00000000.scaffold.faa.tgz](ftp://ftp.ncbi.nlm.nih.gov/genomes/Bacteria_DRAFT/Desulfonatronospira_thiodismutans_ASO3_1_uid55423/NZ_ACJN00000000.scaffold.faa.tgz) |  |  |  |
| *Desulfotalea psychrophila* LSv54 | [NC_006138.faa](ftp://ftp.ncbi.nlm.nih.gov/genomes/Bacteria/Desulfotalea_psychrophila_LSv54_uid58153/NC_006138.faa) | [NC_006139.faa](ftp://ftp.ncbi.nlm.nih.gov/genomes/Bacteria/Desulfotalea_psychrophila_LSv54_uid58153/NC_006139.faa) | [NC_006140.faa](ftp://ftp.ncbi.nlm.nih.gov/genomes/Bacteria/Desulfotalea_psychrophila_LSv54_uid58153/NC_006140.faa) |  |
| *Desulfotignum phosphitoxidans* DSM 13687 | [NZ_APJX00000000.scaffold.faa.tgz](ftp://ftp.ncbi.nlm.nih.gov/genomes/Bacteria_DRAFT/Desulfotignum_phosphitoxidans_DSM_13687_uid196478/NZ_APJX00000000.scaffold.faa.tgz) |  |  |  |
| *Desulfovibrio aespoeensis* Aspo-2 | [NC_014844.faa](ftp://ftp.ncbi.nlm.nih.gov/genomes/Bacteria/Desulfovibrio_aespoeensis_Aspo_2_uid42613/NC_014844.faa) |  |  |  |
| *Desulfovibrio africanus* PCS | [NZ_AOSV00000000.scaffold.faa.tgz](ftp://ftp.ncbi.nlm.nih.gov/genomes/Bacteria_DRAFT/Desulfovibrio_africanus_PCS_uid192736/NZ_AOSV00000000.scaffold.faa.tgz) |  |  |  |
| *Desulfovibrio africanus* str. Walvis Bay | [NC_016629.faa](ftp://ftp.ncbi.nlm.nih.gov/genomes/Bacteria/Desulfovibrio_africanus_Walvis_Bay_uid66847/NC_016629.faa) |  |  |  |
| *Desulfovibrio alaskensis* G20 | [NC_007519.faa](ftp://ftp.ncbi.nlm.nih.gov/genomes/Bacteria/Desulfovibrio_alaskensis_G20_uid57941/NC_007519.faa) |  |  |  |
| *Desulfovibrio desulfuricans* ND132 | [NC_016803.faa](ftp://ftp.ncbi.nlm.nih.gov/genomes/Bacteria/Desulfovibrio_desulfuricans_ND132_uid63159/NC_016803.faa) |  |  |  |
| *Desulfovibrio desulfuricans* ATCC 27774 | [NC_011883.faa](ftp://ftp.ncbi.nlm.nih.gov/genomes/Bacteria/Desulfovibrio_desulfuricans_ATCC_27774_uid59213/NC_011883.faa) |  |  |  |
| *Desulfovibrio fructosovorans* JJ | [NZ_AECZ00000000.scaffold.faa.tgz](ftp://ftp.ncbi.nlm.nih.gov/genomes/Bacteria_DRAFT/Desulfovibrio_fructosovorans_JJ_uid51537/NZ_AECZ00000000.scaffold.faa.tgz) |  |  |  |
| *Desulfovibrio hydrothermalis* AM13 | [NC_020055.faa](ftp://ftp.ncbi.nlm.nih.gov/genomes/Bacteria/Desulfovibrio_hydrothermalis_AM13___DSM_14728_uid184831/NC_020055.faa) | [NC_019953.faa](ftp://ftp.ncbi.nlm.nih.gov/genomes/Bacteria/Desulfovibrio_hydrothermalis_AM13___DSM_14728_uid184831/NC_019953.faa) |  |  |
| *Desulfovibrio magneticus* RS-1 | [NC_012796.faa](ftp://ftp.ncbi.nlm.nih.gov/genomes/Bacteria/Desulfovibrio_magneticus_RS_1_uid59309/NC_012796.faa) | [NC_012795.faa](ftp://ftp.ncbi.nlm.nih.gov/genomes/Bacteria/Desulfovibrio_magneticus_RS_1_uid59309/NC_012795.faa) | [NC_012797.faa](ftp://ftp.ncbi.nlm.nih.gov/genomes/Bacteria/Desulfovibrio_magneticus_RS_1_uid59309/NC_012797.faa) |  |
| *Desulfovibrio magneticus* str. Maddingley MBC34 | [NZ_ALAO00000000.scaffold.faa.tgz](ftp://ftp.ncbi.nlm.nih.gov/genomes/Bacteria_DRAFT/Desulfovibrio_magneticus_Maddingley_MBC34_uid178559/NZ_ALAO00000000.scaffold.faa.tgz) |  |  |  |
| *Desulfovibrio piezophilus* C1TLV30 | [NC_020409.faa](ftp://ftp.ncbi.nlm.nih.gov/genomes/Bacteria/Desulfovibrio_piezophilus_C1TLV30_uid190704/NC_020409.faa) |  |  |  |
| *Desulfovibrio piger* ATCC 29098 | [NZ_ABXU00000000.scaffold.faa.tgz](ftp://ftp.ncbi.nlm.nih.gov/genomes/Bacteria_DRAFT/Desulfovibrio_piger_ATCC_29098_uid54519/NZ_ABXU00000000.scaffold.faa.tgz) |  |  |  |
| *Desulfovibrio salexigens* DSM 2638 | [NC_012881.faa](ftp://ftp.ncbi.nlm.nih.gov/genomes/Bacteria/Desulfovibrio_salexigens_DSM_2638_uid59223/NC_012881.faa) |  |  |  |
| *Desulfovibrio* sp. 3_1_syn3 | [NZ_ADDR00000000.scaffold.faa.tgz](ftp://ftp.ncbi.nlm.nih.gov/genomes/Bacteria_DRAFT/Desulfovibrio_3_1_syn3_uid51597/NZ_ADDR00000000.scaffold.faa.tgz) |  |  |  |
| *Desulfovibrio* sp. 6_1_46AFAA | [NZ_ACWM00000000.scaffold.faa.tgz](ftp://ftp.ncbi.nlm.nih.gov/genomes/Bacteria_DRAFT/Desulfovibrio_6_1_46AFAA_uid72975/NZ_ACWM00000000.scaffold.faa.tgz) |  |  |  |
| *Desulfovibrio* sp. A2 | [NZ_AGFG00000000.scaffold.faa.tgz](ftp://ftp.ncbi.nlm.nih.gov/genomes/Bacteria_DRAFT/Desulfovibrio_A2_uid73581/NZ_AGFG00000000.scaffold.faa.tgz) |  |  |  |
| *Desulfovibrio* sp. FW1012B | [NZ_ADFE00000000.scaffold.faa.tgz](ftp://ftp.ncbi.nlm.nih.gov/genomes/Bacteria_DRAFT/Desulfovibrio_FW1012B_uid43335/NZ_ADFE00000000.scaffold.faa.tgz) |  |  |  |
| *Desulfovibrio* sp. U5L | [NZ_AHMC00000000.scaffold.faa.tgz](ftp://ftp.ncbi.nlm.nih.gov/genomes/Bacteria_DRAFT/Desulfovibrio_U5L_uid162937/NZ_AHMC00000000.scaffold.faa.tgz) |  |  |  |
| *Desulfovibrio vulgaris* DP4 | [NC_008751.faa](ftp://ftp.ncbi.nlm.nih.gov/genomes/Bacteria/Desulfovibrio_vulgaris_DP4_uid58679/NC_008751.faa) | [NC_008741.faa](ftp://ftp.ncbi.nlm.nih.gov/genomes/Bacteria/Desulfovibrio_vulgaris_DP4_uid58679/NC_008741.faa) |  |  |
| *Desulfovibrio vulgaris* RCH1 | [NC_017310.faa](ftp://ftp.ncbi.nlm.nih.gov/genomes/Bacteria/Desulfovibrio_vulgaris_RCH1_uid161961/NC_017310.faa) | [NC_017311.faa](ftp://ftp.ncbi.nlm.nih.gov/genomes/Bacteria/Desulfovibrio_vulgaris_RCH1_uid161961/NC_017311.faa) |  |  |
| *Desulfovibrio vulgaris* str. 'Miyazaki F' | [NC_011769.faa](ftp://ftp.ncbi.nlm.nih.gov/genomes/Bacteria/Desulfovibrio_vulgaris__Miyazaki_F__uid59089/NC_011769.faa) |  |  |  |
| *Desulfovibrio vulgaris* str. Hildenborough | [NC_002937.faa](ftp://ftp.ncbi.nlm.nih.gov/genomes/Bacteria/Desulfovibrio_vulgaris_Hildenborough_uid57645/NC_002937.faa) | [NC_005863.faa](ftp://ftp.ncbi.nlm.nih.gov/genomes/Bacteria/Desulfovibrio_vulgaris_Hildenborough_uid57645/NC_005863.faa) |  |  |
| *Desulfurivibrio alkaliphilus* AHT2 | [NC_014216.faa](ftp://ftp.ncbi.nlm.nih.gov/genomes/Bacteria/Desulfurivibrio_alkaliphilus_AHT2_uid49487/NC_014216.faa) |  |  |  |
| *Desulfuromonas acetoxidans* DSM 684 | [NZ_AAEW00000000.scaffold.faa.tgz](ftp://ftp.ncbi.nlm.nih.gov/genomes/Bacteria_DRAFT/Desulfuromonas_acetoxidans_DSM_684_uid54145/NZ_AAEW00000000.scaffold.faa.tgz) |  |  |  |
| *Geobacter bemidjiensis* Bem | [NC_011146.faa](ftp://ftp.ncbi.nlm.nih.gov/genomes/Bacteria/Geobacter_bemidjiensis_Bem_uid58749/NC_011146.faa) |  |  |  |
| *Geobacter daltonii* FRC-32 | [NC_011979.faa](ftp://ftp.ncbi.nlm.nih.gov/genomes/Bacteria/Geobacter_FRC_32_uid58543/NC_011979.faa) |  |  |  |
| *Geobacter lovleyi* SZ | [NC_010814.faa](ftp://ftp.ncbi.nlm.nih.gov/genomes/Bacteria/Geobacter_lovleyi_SZ_uid58713/NC_010814.faa) |  |  |  |
| *Geobacter metallireducens* GS-15 | [NC_007517.faa](ftp://ftp.ncbi.nlm.nih.gov/genomes/Bacteria/Geobacter_metallireducens_GS_15_uid57731/NC_007517.faa) | [NC_007515.faa](ftp://ftp.ncbi.nlm.nih.gov/genomes/Bacteria/Geobacter_metallireducens_GS_15_uid57731/NC_007515.faa) |  |  |
| *Geobacter metallireducens* RCH3 | [NZ_AGJM00000000.scaffold.faa.tgz](ftp://ftp.ncbi.nlm.nih.gov/genomes/Bacteria_DRAFT/Geobacter_metallireducens_RCH3_uid179906/NZ_AGJM00000000.scaffold.faa.tgz) |  |  |  |
| *Geobacter* sp. M18 | [NC_014973.faa](ftp://ftp.ncbi.nlm.nih.gov/genomes/Bacteria/Geobacter_M18_uid55771/NC_014973.faa) |  |  |  |
| *Geobacter* sp. M21 | [NC_012918.faa](ftp://ftp.ncbi.nlm.nih.gov/genomes/Bacteria/Geobacter_M21_uid59037/NC_012918.faa) |  |  |  |
| *Geobacter sulfurreducens* KN400 | [NC_017454.faa](ftp://ftp.ncbi.nlm.nih.gov/genomes/Bacteria/Geobacter_sulfurreducens_KN400_uid161977/NC_017454.faa) |  |  |  |
| *Geobacter sulfurreducens* PCA | [NC_002939.faa](ftp://ftp.ncbi.nlm.nih.gov/genomes/Bacteria/Geobacter_sulfurreducens_PCA_uid57743/NC_002939.faa) |  |  |  |
| *Geobacter uraniireducens* Rf4 | [NC_009483.faa](ftp://ftp.ncbi.nlm.nih.gov/genomes/Bacteria/Geobacter_uraniireducens_Rf4_uid58475/NC_009483.faa) |  |  |  |
| *Haliangium ochraceum* DSM 14365 | [NC_013440.faa](ftp://ftp.ncbi.nlm.nih.gov/genomes/Bacteria/Haliangium_ochraceum_DSM_14365_uid41425/NC_013440.faa) |  |  |  |
| *Hippea maritima* DSM 10411 | [NC_015318.faa](ftp://ftp.ncbi.nlm.nih.gov/genomes/Bacteria/Hippea_maritima_DSM_10411_uid65267/NC_015318.faa) |  |  |  |
| *Lawsonia intracellularis* N343 | [NC_020127.faa](ftp://ftp.ncbi.nlm.nih.gov/genomes/Bacteria/Lawsonia_intracellularis_N343_uid186598/NC_020127.faa) | [NC_020128.faa](ftp://ftp.ncbi.nlm.nih.gov/genomes/Bacteria/Lawsonia_intracellularis_N343_uid186598/NC_020128.faa) | [NC_020129.faa](ftp://ftp.ncbi.nlm.nih.gov/genomes/Bacteria/Lawsonia_intracellularis_N343_uid186598/NC_020129.faa) | [NC_020130.faa](ftp://ftp.ncbi.nlm.nih.gov/genomes/Bacteria/Lawsonia_intracellularis_N343_uid186598/NC_020130.faa) |
| *Lawsonia intracellularis* PHE/MN1-00 | [NC_008011.faa](ftp://ftp.ncbi.nlm.nih.gov/genomes/Bacteria/Lawsonia_intracellularis_PHE_MN1_00_uid61575/NC_008011.faa) | [NC_008012.faa](ftp://ftp.ncbi.nlm.nih.gov/genomes/Bacteria/Lawsonia_intracellularis_PHE_MN1_00_uid61575/NC_008012.faa) | [NC_008013.faa](ftp://ftp.ncbi.nlm.nih.gov/genomes/Bacteria/Lawsonia_intracellularis_PHE_MN1_00_uid61575/NC_008013.faa) | [NC_008014.faa](ftp://ftp.ncbi.nlm.nih.gov/genomes/Bacteria/Lawsonia_intracellularis_PHE_MN1_00_uid61575/NC_008014.faa) |
| *Myxococcus fulvus* HW-1 | [NC_015711.faa](ftp://ftp.ncbi.nlm.nih.gov/genomes/Bacteria/Myxococcus_fulvus_HW_1_uid68443/NC_015711.faa) |  |  |  |
| *Myxococcus stipitatus* DSM 14675 | [NC_020126.faa](ftp://ftp.ncbi.nlm.nih.gov/genomes/Bacteria/Myxococcus_stipitatus_DSM_14675_uid186549/NC_020126.faa) |  |  |  |
| *Myxococcus xanthus* DK 1622 | [NC_008095.faa](ftp://ftp.ncbi.nlm.nih.gov/genomes/Bacteria/Myxococcus_xanthus_DK_1622_uid58003/NC_008095.faa) |  |  |  |
| *Pelobacter carbinolicus* DSM 2380 | [NC_007498.faa](ftp://ftp.ncbi.nlm.nih.gov/genomes/Bacteria/Pelobacter_carbinolicus_DSM_2380_uid58241/NC_007498.faa) |  |  |  |
| *Pelobacter propionicus* DSM 2379 | [NC_008609.faa](ftp://ftp.ncbi.nlm.nih.gov/genomes/Bacteria/Pelobacter_propionicus_DSM_2379_uid58255/NC_008609.faa) | [NC_008608.faa](ftp://ftp.ncbi.nlm.nih.gov/genomes/Bacteria/Pelobacter_propionicus_DSM_2379_uid58255/NC_008608.faa) | [NC_008607.faa](ftp://ftp.ncbi.nlm.nih.gov/genomes/Bacteria/Pelobacter_propionicus_DSM_2379_uid58255/NC_008607.faa) |  |
| *Plesiocystis pacifica* SIR-1 | [NZ_ABCS00000000.scaffold.faa.tgz](ftp://ftp.ncbi.nlm.nih.gov/genomes/Bacteria_DRAFT/Plesiocystis_pacifica_SIR_1_uid54707/NZ_ABCS00000000.scaffold.faa.tgz) |  |  |  |
| *SAR324 cluster bacterium* JCVI-SC AAA005 | [NZ_AGAU00000000.scaffold.faa.tgz](ftp://ftp.ncbi.nlm.nih.gov/genomes/Bacteria_DRAFT/SAR324_cluster_bacterium_JCVI_SC_AAA005_uid86871/NZ_AGAU00000000.scaffold.faa.tgz) |  |  |  |
| *SAR324 cluster bacterium* SCGC AAA001-C10 | [NZ_AFIB00000000.scaffold.faa.tgz](ftp://ftp.ncbi.nlm.nih.gov/genomes/Bacteria_DRAFT/SAR324_cluster_bacterium_SCGC_AAA001_C10_uid171686/NZ_AFIB00000000.scaffold.faa.tgz) |  |  |  |
| *Sorangium cellulosum* So ce56 | [NC_010162.faa](ftp://ftp.ncbi.nlm.nih.gov/genomes/Bacteria/Sorangium_cellulosum__So_ce_56__uid61629/NC_010162.faa) |  |  |  |
| *Stigmatella aurantiaca* DW4/3-1 | [NC_014623.faa](ftp://ftp.ncbi.nlm.nih.gov/genomes/Bacteria/Stigmatella_aurantiaca_DW4_3_1_uid158509/NC_014623.faa) |  |  |  |
| *Syntrophobacter fumaroxidans* MPOB | [NC_008554.faa](ftp://ftp.ncbi.nlm.nih.gov/genomes/Bacteria/Syntrophobacter_fumaroxidans_MPOB_uid58177/NC_008554.faa) |  |  |  |
| *Syntrophus aciditrophicus* SB | [NC_007759.faa](ftp://ftp.ncbi.nlm.nih.gov/genomes/Bacteria/Syntrophus_aciditrophicus_SB_uid58539/NC_007759.faa) |  |  |  |
