# Supplementary material for: Evolution and Design Governing Signal Precision and Amplification in a Bacterial Chemosensory Pathway
Source: PLoS Genet. 2015 Aug 20;11(8):e1005460. doi: 10.1371/journal.pgen.1005460 (PMC4546325; doi:10.1371/journal.pgen.1005460)
Supplement: S1 Text — (DOCX) [file pgen.1005460.s018.docx]

**S1 Text: Supplemental References**

1. Bustamante VH, Martinez-Flores I, Vlamakis HC, Zusman DR. Analysis of the Frz signal transduction system of Myxococcus xanthus shows the importance of the conserved C-terminal region of the cytoplasmic chemoreceptor FrzCD in sensing signals. Mol Microbiol. 2004;53: 1501–13.

2. Li YN, Bustamante VH, Lux R, Zusman D, Shi WY. Divergent regulatory pathways control A and S motility in Myxococcus xanthus through FrzE, a CheA-CheY fusion protein. Journal of Bacteriology. 2005;187: 1716–1723.

3. Inclan YF, Laurent S, Zusman DR. The receiver domain of FrzE, a CheA-CheY fusion protein, regulates the CheA histidine kinase activity and downstream signalling to the A- and S-motility systems of Myxococcus xanthus. Mol Microbiol. 2008;68: 1328–39.

4. Inclan YF, Vlamakis HC, Zusman DR. FrzZ, a dual CheY-like response regulator, functions as an output for the Frz chemosensory pathway of Myxococcus xanthus. Mol Microbiol. 2007;65: 90–102.

5. Mauriello EM, Nan B, Zusman DR. AglZ regulates adventurous (A-) motility in Myxococcus xanthus through its interaction with the cytoplasmic receptor, FrzCD. Mol Microbiol. 2009;72: 964–77.

6. Mignot T, Merlie JP, Zusman DR. Regulated pole-to-pole oscillations of a bacterial gliding motility protein. Science. 2005;310: 855–857.

7. Sun M, Wartel M, Cascales E, Shaevitz JW, Mignot T. Motor-driven intracellular transport powers bacterial gliding motility. Proc Natl Acad Sci USA. 2011;108: 7559–7564. doi:10.1073/pnas.1101101108

8. Zhang Y, Franco M, Ducret A, Mignot T. A bacterial Ras-like small GTP-binding protein and its cognate GAP establish a dynamic spatial polarity axis to control directed motility. PLoS Biol. 2010;8: e1000430. doi:10.1371/journal.pbio.1000430

9. Zhang Y, Guzzo M, Ducret A, Li Y-Z, Mignot T. A dynamic response regulator protein modulates G-protein-dependent polarity in the bacterium Myxococcus xanthus. PLoS Genet. 2012;8: e1002872. doi:10.1371/journal.pgen.1002872

10. Molle V, Kremer L, Girard-Blanc C, Besra GS, Cozzone AJ, Prost J-F. An FHA phosphoprotein recognition domain mediates protein EmbR phosphorylation by PknH, a Ser/Thr protein kinase from Mycobacterium tuberculosis. Biochemistry. 2003;42: 15300–15309. doi:10.1021/bi035150b
